# The Neuroscience of Religious and Spiritual Practices: A Systematic Review of Neurotheological Evidence

**DOI:** 10.1002/brb3.71733

**Published:** 2026-08-30

**Authors:** Waqar Husain, Achraf Ammar, Urwa‐Tul Wusqa, Maha Farooq, Zehra Tariq, Seithikurippu R. Pandi‐Perumal, Ahmed S. BaHammam, Khaled Trabelsi, Mark D. Griffiths, Amir Pakpour, Haitham Jahrami

**Affiliations:** ^1^ Department of Humanities COMSATS University Islamabad, Islamabad Campus Islamabad Pakistan; ^2^ Department of Training and Movement Science, Institute of Sport Science Johannes Gutenberg‐University Mainz Mainz Germany; ^3^ Research Laboratory, Molecular Bases of Human Pathology, LR19ES13, Faculty of Medicine of Sfax University of Sfax Sfax Tunisia; ^4^ Nutrition and Food Technology Department, Agriculture School The University of Jordan Amman Jordan; ^5^ Centre For Research and Development Chandigarh University Mohali Punjab India; ^6^ Central Research Facility (CRF), Dr D.Y. Patil Medical College Hospital and Research Centre Dr D.Y. Patil Vidyapeeth, Pimpri, 411018, Pune India; ^7^ Department of Medicine, University Sleep Disorders Center, College of Medicine King Saud University Riyadh Saudi Arabia; ^8^ The Strategic Technologies Program of the National Plan For Sciences and Technology and Innovation in the Kingdom of Saudi Arabia Riyadh Saudi Arabia; ^9^ Research Laboratory Education, Motricité, Sport et Santé, EM2S, LR19JS01, High Institute of Sport and Physical Education of Sfax University of Sfax Sfax Tunisia; ^10^ Department of Movement Sciences and Sports Training, School of Sport Science The University of Jordan Amman Jordan; ^11^ Nottingham Insitute of Psychology Nottingham Trent University Nottingham UK; ^12^ Department of Nursing, School of Health and Welfare Jönköping University Jönköping Sweden; ^13^ Division of Anesthesia, Operation, and Intensive Care Södertälje Hospital Södertälje Sweden; ^14^ Government Hospitals Manama Bahrain; ^15^ Department of Psychiatry, College of Medicine and Health Sciences Arabian Gulf University Manama Bahrain

**Keywords:** default mode network, meditation, mental health, neural activity, neuroimaging, neuroplasticity, neurotheology, prayer, religious practices, spiritual practices

## Abstract

**Background:**

Religious and spiritual practices are among the most ancient and universal human behaviors, yet their neurobiological substrates remain incompletely characterized. Neurotheology—the scientific study of the relationship between spiritual experience and brain function—has produced growing evidence that such practices generate measurable, systematic, and neuroplastic changes in brain structure and function. However, prior reviews have been limited to single practice types, narrow evidence bases, or narrative methods, with none synthesizing the full spectrum of traditions, practices, and neuroimaging modalities in a single review.

**Methods:**

Adhering to the PRISMA 2020 guidelines and registered on the Open Science Framework, *Scopus, PubMed/MEDLINE*, and *ProQuest* (23 sub‐databases) were searched from inception to June 30, 2026. Eligible studies comprised those using objective neuroimaging or neurophysiological techniques. After multi‐stage screening, 105 studies (1998–2026) were synthesized narratively using the synthesis without meta‐analysis (SWiM) framework, given substantial heterogeneity.

**Results:**

Studies spanned Buddhist, Christian, Islamic, Hindu, Santo Daime, Spiritist, Sant Mat, and mixed traditions, examining meditation, prayer, chanting, recitation, worship, retreat, mystical experience, mediumistic trance, and ritual altered states. Consistent engagement appeared across the prefrontal cortex, anterior cingulate, default mode network (DMN), insula, amygdala, hippocampus, temporoparietal junction, and dopaminergic/serotonergic systems. Practice‐specific signatures emerged: meditation (DMN suppression, prefrontal augmentation), prayer (social‐cognition networks), chanting/recitation (limbic deactivation, gamma/delta enhancement), and mystical states (most distributed profiles). Long‐term practitioners showed structural neuroplasticity, and clinical‐population studies linked these circuits to depression, anxiety, and stress.

**Conclusions:**

Religious and spiritual practices produce consistent, practice‐specific, and neuroplastic modifications to brain systems governing attention, emotion regulation, self‐referential processing, and reward—supporting a plausible mechanistic link between spiritual engagement and mental health, and positioning neurotheology as an empirically grounded dialogue between contemplative tradition and clinical neuroscience.

## Graphical abstract

Systematic synthesis of 105 neuroimaging and neurophysiological studies demonstrates that religious and spiritual practices, including meditation, prayer, chanting, worship, and mystical experiences, produce practice‐specific neuroplastic changes in brain networks governing attention, self‐referential processing, emotion regulation, connectivity, reward, and mental health.

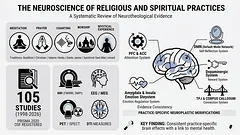

**Abbreviations**
ACC: anterior cingulate cortex
ALE: activation likelihood estimation
BOLD: blood oxygenation level‐dependent
DMN: default mode network
DTI: diffusion tensor imaging
ECG: electrocardiography
EEG: electroencephalography
ERP: event‐related potential
fMRI: functional magnetic resonance imaging
GSR: Galvanic skin response
HPA: hypothalamic‐pituitary‐adrenal
HRV: heart rate variability
LC/MS/MS: liquid chromatography with tandem mass spectrometry
MEG: magnetoencephalography
MRI: magnetic Resonance imaging
OSF: open science framework
PCC: posterior cingulate cortex
PET: positron emission tomography
PFC: prefrontal cortex
PRISMA: Preferred Reporting Items for Systematic Reviews and Meta‐Analyses
PTSD: post‐traumatic stress disorder
qPCR: quantitative polymerase chain reaction
sLORETA
: standardized low‐resolution brain electromagnetic tomography/ standardized low‐resolution electromagnetic tomography
SPECT: single‐photon emission computed tomography
SWiM: synthesis without meta‐analysis
TM: transcendental meditation

## 1 Introduction

### 1.1 Background and Significance

Religion and spirituality represent two of the most pervasive and enduring dimensions of human experience. Approximately 84% of the global population self‐identifies with a religious tradition (Hackett et al. 2015). Despite this large proportion, the scientific study of how such practices affect the brain remained largely outside mainstream neuroscience until the closing decades of the 20th century. The emergence of non‐invasive neuroimaging technologies, particularly functional magnetic resonance imaging (fMRI), positron emission tomography (PET), and high‐density electroencephalography (EEG), transformed the scientific landscape by making it possible to observe the living human brain during states of prayer, meditation, and spiritual experience. The resulting body of research has generated substantial evidence that religious and spiritual practices are neurologically active behaviors that engage, modify, and in some cases permanently reshape brain structure and function. This recognition gave rise to neurotheology, defined broadly as the scientific investigation of the relationship between brain function and religious or spiritual experience (Newberg 2014). Its findings carry implications for mental health, cognitive function, and human well‐being that science cannot responsibly ignore.

### 1.2 Theoretical Foundations

The theoretical basis of neurotheology rests on several interconnected propositions. First, all human experience, including spiritual experience, is mediated by the brain. This is not a reductionist claim but the methodological commitment that neural correlates of spiritual states are legitimate objects of scientific investigation (Newberg and Waldman 2009). Second, neuroplasticity, the brain's capacity to reorganize structurally and functionally in response to experience (Dzeladini et al. 2014), makes it scientifically reasonable to hypothesize that sustained religious and spiritual practice induces neuroplastic change. Third, different aspects of religious experience are mediated by distinguishable neural networks rather than a single “God module” (Legare and Visala 2011). It has been found that (i) prefrontal cortex (PFC) and anterior cingulate cortex (ACC) subserve voluntary attention; (ii) the default mode network (DMN) mediates self‐referential processing; (iii) the temporoparietal junction (TPJ) supports social cognition; the limbic system processes emotional salience; and (v) subcortical dopaminergic and serotonergic circuits modulate reward and transcendence (McNamara and Grafman 2024). Fourth, a distinction between state effects (transient changes during practice) and trait effects (durable neuroplastic modifications from sustained practice) is conceptually important for interpreting findings across studies.

### 1.3 Historical Development

Early EEG studies in the 1960s and 1970s identified characteristic increases in frontal alpha and theta activity during Transcendental Meditation (TM), establishing that the meditating brain is neurophysiologically distinctive (Banquet 1973; Wallace 1970). Pioneering single‐photon emission computed tomography (SPECT) neuroimaging by Newberg and colleagues documented increased prefrontal activity and altered parietal perfusion during Tibetan Buddhist meditation and Franciscan centering prayer, establishing neurotheology as a recognized field (Newberg et al. 2001, 2003). Subsequent proliferation of fMRI expanded investigation to large‐scale functional connectivity, DMN dynamics, and specific experiential dimensions of practice. By the 2010s, structural neuroimaging using voxel‐based morphometry and diffusion tensor imaging (DTI) documented lasting morphological differences between long‐term practitioners and non‐practitioners, including increased gray matter in the hippocampus, PFC, and cingulate regions following mindfulness training (Hölzel et al. 2011), altered corpus callosum morphology in habitual prayer practitioners (Baykara et al. 2023), and neuroprotective cortical thickness patterns associated with altruistic spiritual phenotypes (Miller et al. 2021).

### 1.4 Knowledge Gap and Rationale

Despite growing scientific interest in neurotheology, the existing review literature remains fragmented along three lines: (i) restriction to a single practice type, (ii) restriction in the breadth or recency of included evidence, and (iii) a narrative rather than systematic methodology. The most methodologically rigorous prior work has focused almost exclusively on meditation. Fox et al. (2014) conducted a systematic review and meta‐analysis of structural neuroimaging among meditation practitioners, synthesizing 21 studies with approximately 300 practitioners (Fox et al. 2014). A companion meta‐analysis by the same group reviewed 78 functional neuroimaging studies and used activation likelihood estimation (ALE) to meta‐analyze 257 peak foci from 31 of these experiments to characterize four common meditation styles (Fox et al. 2016). This meditation‐only focus has also persisted into more recent literature. Calderone et al. (2024) conducted a systematic review of neurobiological changes associated with mindfulness and meditation, again confined to a single practice category rather than extending across religious and spiritual traditions (Calderone et al. 2024). These three prior reviews were explicitly restricted to meditation and did not extend to prayer, chanting, recitation, worship, or mystical experience.

A parallel, single‐practice pattern has continued into the most recent literature. Perry et al. (2025) published a systematic review of chanting, specifically synthesizing 24 neuroimaging studies drawn from only two databases (*PsycINFO* and *PubMed*), explicitly noting in the abstract that *“while previous reviews have considered the neurophysiological impact of meditation and spirituality, chanting has received limited systematic investigation”* (Perry et al. 2025; p., 1). Similarly, Haverkamp et al. (2025) conducted a systematic review that was restricted to Christian prayer and its convergence with attachment neuroscience, synthesizing 44 studies across eight databases but excluding all other traditions and practice types by design (Haverkamp et al. 2025). A coordinate‐based ALE meta‐analysis by Yeung et al. (2025) similarly restricted its scope to neuroimaging studies of Christian religious behavior, including prayer and Bible recitation, pooling only 11 eligible studies and likewise excluding all other traditions (Yeung et al. 2025). Together, these reviews demonstrate that even the most current systematic literature in this field remains organized around single practices or single traditions rather than a unified, cross‐traditional account.

The two reviews that have attempted broader tradition coverage are each limited in scope or currency. Rim et al. (2019) conducted a systematic review of the neurobiological correlates of religion and spirituality but identified only 25 eligible studies, restricted their search to studies published between 1990 and 2017, and did not organize findings by discrete practice category or map them onto specific mental health circuitry (Rim et al. 2019). Rosmarin et al. (2022) subsequently reviewed the intersection of spirituality/religion, mental health, and neurobiology but applied a narrow triple‐intersection inclusion criterion that yielded only 18 studies, explicitly excluding studies of secular mindfulness, and organized findings around psychiatric diagnoses (depression, anxiety, substance misuse, and psychosis) rather than around practice type or cross‐traditional neural convergence (Rosmarin et al. 2022). Neither review incorporated the substantial expansion of neurotheological neuroimaging published after 2017, which the present review demonstrates to be considerable: 84 of the 105 studies identified in the present review were published after 2009 (80%), and 60 studies were published in 2017 or later (57%).

The remaining influential contributions to this literature, including Newberg's (2014) foundational synthesis, Cahn and Polich's (2006) review of meditation EEG, event‐related potential (ERP), and neuroimaging studies, and van Elk and Aleman's (2017) predictive‐processing account of religious experience, were narrative rather than systematic, lack a pre‐specified search strategy or Preferred Reporting Items for Systematic Reviews and Meta‐Analyses (PRISMA)‐compliant flow, and were not designed to support reproducible, criterion‐based inclusion decisions.

Taken together, no prior review has applied a systematic, pre‐registered, PRISMA‐compliant methodology to the full breadth of neurotheological evidence spanning multiple religious and spiritual traditions, multiple neuroimaging and neurophysiological modalities, and multiple practice categories within a single unified framework. This represents the first gap that the present review addresses. A second gap is the absence of cross‐traditional comparative synthesis. Because existing reviews are each confined to a single practice (e.g., meditation, chanting, Christian prayer) or a narrow proportion of the literature, the field lacks a clear account of which neural effects are universal across religious and spiritual practice and which are practice‐specific or tradition‐specific. A third gap is the absence of explicit mapping from practice‐induced neural changes onto the circuit‐level pathologies of depression, anxiety, post‐traumatic stress disorder (PTSD), and addiction. Prior broad‐scope reviews (Rim et al. 2019; Rosmarin et al. 2022) catalogued affected brain regions or organized findings by diagnosis, respectively, but neither systematically linked practice‐specific neural signatures across traditions to the specific circuits implicated in these conditions. The present review addresses all three gaps by synthesizing 105 studies across eight traditions and seven modalities within a pre‐registered PRISMA 2020‐compliant framework, organized simultaneously by practice category and by convergence onto neural systems of established mental health relevance.

## 2 Methods

### 2.1 Study Design and Registration

The present systematic review was conducted in accordance with the PRISMA 2020 guidelines (Page et al. 2022). The PRISMA Checklist has been provided as Supporting Information 1. To enhance transparency, reproducibility, and methodological accountability, the review protocol and analytical framework were registered on the open science framework (OSF; https://osf.io/wm6at/overview). Due to substantial heterogeneity across religious and spiritual practices, participant populations, neuroimaging modalities, study designs, comparator conditions, and reported neural outcomes, a narrative synthesis approach, guided by the Synthesis Without Meta‐analysis (SWiM) framework (Campbell et al. 2020), was adopted in lieu of quantitative meta‐analysis.

### 2.2 Conceptual Definitions and Classification of Practices

For the purposes of the present review, religious practices were defined as intentional behaviors, rituals, contemplative acts, or devotional experiences embedded within, prescribed by, or explicitly interpreted through an organized religious tradition, scripture, doctrine, community, ritual system, or theistic framework. Examples included prayer, Salat, Quranic recitation, biblical recitation, Christian worship, glossolalia, devotional chanting, and religiously framed mystical experience. Spiritual practices were defined more broadly as intentional contemplative, meditative, or ritualized practices directed toward self‐transcendence, ultimate meaning, sacredness, compassion, equanimity, altered self‐processing, or perceived connection with a reality beyond ordinary ego‐centered experience, whether or not the practice was embedded within an institutional religion. This distinction follows the common conceptual separation between religion as tradition‐based beliefs, practices, and rituals related to the transcendent, and spirituality as a broader, more personal, and sometimes non‐institutional orientation toward meaning, transcendence, or the sacred.

Because many contemplative practices have both religious origins and secular contemporary implementations (Landau and Jones 2021), classifying mindfulness and meditation studies required a nuanced approach rather than automatic designation as religious. During study selection and data extraction, practices were categorized based on their contextual framing and operational characteristics. Studies were designated as religious if the practice was explicitly embedded within a named religious tradition, scripture, doctrine, ritual system, deity‐related practice, or faith community. Studies were classified as spiritual/contemplative when the practice involved meditation, self‐transcendence, compassion cultivation, altered self‐processing, or contemplative awareness, independent of affiliation with an organized religion, or when the study explicitly examined meditative, transcendence‐oriented, self‐regulatory, devotional, or spiritually meaningful practice using objective neural or neurophysiological measures.

Studies were categorized as mixed or secular‐derived when the practice, despite identifiable religious or spiritual origins, was presented in a contemporary secular, clinical, or experimental context (e.g., mindfulness‐based stress reduction or secular mindfulness meditation). Their inclusion was justified because such interventions represent contemporary adaptations of historically spiritual practices and target neural processes central to contemplative experience, including attention regulation, self‐referential processing, interoception, emotion regulation, and neuroplasticity. This classification framework allowed the review to examine a continuum of practices ranging from explicitly religious rituals to secularized contemplative interventions, while avoiding the assumption that all mindfulness research is inherently religious.

Exclusion criteria included studies where religious or spiritual content was incidental, tasks involved only passive exposure to religious stimuli without active practice engagement, or altered states were pharmacologically induced without explicit religious or spiritual framing.

### 2.3 Eligibility Criteria

Studies were eligible for inclusion if they investigated clearly defined religious, spiritual, contemplative, devotional, or ritual practices or experiences among human participants using at least one objective neuroimaging or neurophysiological measurement technique. Eligible modalities encompassed fMRI, structural magnetic resonance imaging (MRI), resting‐state fMRI, EEG, magnetoencephalography (MEG), PET, SPECT, DTI, functional connectivity analysis, cerebral blood flow imaging, blood oxygenation level‐dependent (BOLD) signal assessment, gray or white matter structural measures, cortical thickness, monoaminergic transporter imaging, electrocardiography (ECG), heart rate variability (HRV), galvanic skin response (GSR), ERPs, quantitative polymerase chain reaction (qPCR), liquid chromatography with tandem mass spectrometry (LC/MS/MS), and standardized low‐resolution electromagnetic tomography (sLORETA) source analysis. Participants of any age, sex, nationality, religious background, health status, or practitioner‐expertise level were eligible.

During study selection and data extraction, practices were categorized according to their contextual framing and operational characteristics. Studies were classified as religious when the practice was explicitly situated within a named religious tradition, scripture, ritual system, deity‐related practice, or faith community. Studies were classified as spiritual/contemplative when the practice involved meditation, self‐transcendence, compassion cultivation, altered self‐processing, or contemplative awareness without necessarily being tied to an organized religion. Studies were classified as mixed or secular‐derived when the practice had identifiable religious or spiritual origins but was delivered in a contemporary secular, clinical, or experimental context, such as mindfulness‐based stress reduction or secular mindfulness meditation. This approach was intended to preserve conceptual clarity while acknowledging that religious, spiritual, and secular contemplative practices often overlap historically, phenomenologically, and neurobiologically.

Studies were excluded if they reported only psychological, behavioral, phenomenological, or self‐report outcomes without objective neural or neurophysiological measurement. Reviews, systematic reviews, meta‐analyses, commentaries, theoretical papers, editorials, dissertations, theses, books, book chapters, magazine articles, newspaper articles, videos, archival non‐journal materials, animal studies, and pharmacologically altered‐state studies without explicit religious or spiritual framing were also excluded. Studies were further excluded if religious or spiritual content was merely incidental (including cases where religious behavior was incidental to neurological or psychiatric pathology rather than the focus of investigation) or if the paradigm involved only passive exposure to religious images, words, music, or symbols without active religious, spiritual, or contemplative engagement.

### 2.4 Search Strategy

A systematic literature search was conducted across three electronic databases: *Scopus, PubMed/MEDLINE*, and *ProQuest*. The search spanned from each database's inception to June 30, 2026. Within *ProQuest*, the search encompassed 23 institutional sub‐databases and collections, including *Academic Video Online, American Periodicals, British Periodicals, Coronavirus Research Database, Ebook Central, Education Magazine Archive, Education Research Index* (1966–current), *Health & Medical Collection, MEDLINE* (1946–current), *Periodicals Archive Online, ProQuest Dissertations & Theses Global, ProQuest One Health & Nursing* (1946–current), *British Nursing Collection* (1994–current), *Health & Medical Research Collection* (1946–current), *ComDisDome* (1950–current), *Consumer Health Database, Healthcare Administration Database, Medical Database, Public Health Database, Sports Medicine & Education Index* (1970–current*), Nursing & Allied Health Collection, Publicly Available Content Database*, and *The Vogue Archive*.

Search strategies were tailored to the syntax of each database and combined terms related to religious and spiritual practices or experiences with terms related to objective neuroimaging and neurophysiological methods. Religious and spiritual terms included but were not limited to “neurotheology” “neuroscience of religion” “spiritual neuroscience” “religious experience” “spiritual experience” “mystical experience” “religious practice” “spiritual practice” “prayer” “personal prayer” “formal prayer” “Christian prayer” “Islamic prayer” “Muslim prayer” “Salat” “Salah” “dhikr” “zikr” “Quran recitation” “Quranic recitation” “scripture recitation” “religious chanting” “devotional chanting” “mantra” “OM chanting” “glossolalia” “speaking in tongues” “spiritual retreat” “worship” “religious coping” “self‐transcendence” and “transcendent experience.”

Neuroimaging and neurophysiological terms included, but were not limited to, “fMRI” “functional magnetic resonance imaging,” “MRI” “structural MRI” “resting‐state fMRI” “EEG” “electroencephalography” “MEG” “magnetoencephalography” “PET” “positron emission tomography” “SPECT” “single‐photon emission computed tomography” “DTI” “diffusion tensor imaging” “functional connectivity” “BOLD” “cerebral blood flow” “grey matter” “gray matter” “white matter” “cortical thickness” “brain volume” “regional brain volume” “dopamine transporter” “serotonin transporter” “neural correlates” “brain activity” and “brain activation.” During screening, searches were limited to English‐language, peer‐reviewed empirical human studies. The complete database‐specific search strategies are provided in Supporting Information 1. *ProQuest* databases were searched as part of the institutional package, but non‐peer‐reviewed and non‐journal materials were excluded during screening. Overlap between *PubMed/MEDLINE* and *ProQuest MEDLINE* was expected and was resolved through deduplication before screening. The complete database‐specific search strategies are provided in Supporting Information 2.

### 2.5 Record Management and Deduplication

Search results from all databases were exported and combined for record management. Duplicate records were identified using a structured deduplication procedure based on digital object identifier, *PubMed* identifier (where available), title, author names, year of publication, journal name, and bibliographic metadata. Automated duplicate detection was supplemented by manual verification to identify records with minor differences in punctuation, capitalization, author formatting, or database indexing. When duplicate reports of the same study were identified, the most complete peer‐reviewed publication was retained. If multiple publications reported distinct outcomes from the same cohort or experiment, they were retained only when they contributed non‐overlapping neural, neurophysiological, or methodological information relevant to the review question. This was managed using *EndNote 2025*.

### 2.6 Study Selection

**FIGURE 1:**
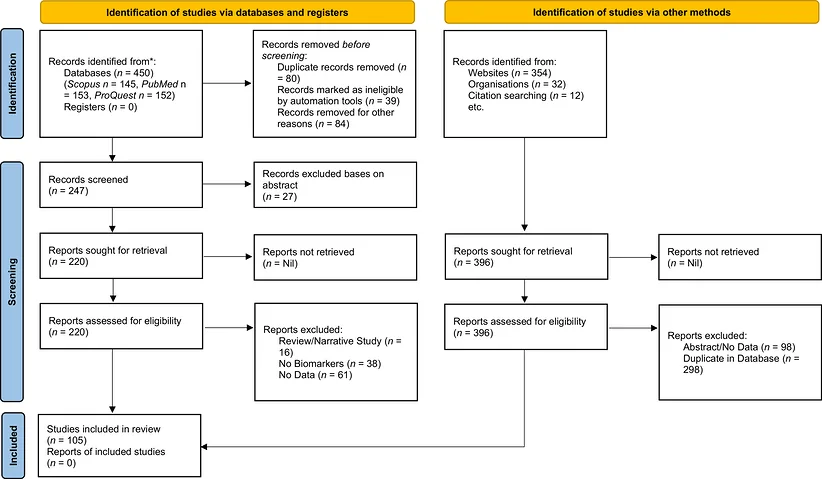
PRISMA flow diagram. Flow diagram depicting the identification, screening, eligibility assessment, and inclusion of studies in accordance with the Preferred Reporting Items for Systematic Reviews and Meta‐Analyses (PRISMA) 2020 guidelines. Records were identified through database searching (*n* = 450; *Scopus n* = 145, *PubMed/MEDLINE n* = 153, *ProQuest n* = 152) and via other methods (websites *n* = 354, organizations *n* = 32, citation searching *n* = 12). Prior to screening, duplicate records (*n* = 80), records flagged as ineligible by automation tools (*n* = 39), and records removed for other reasons (*n* = 84) were excluded. Following title and abstract screening and full‐text eligibility assessment, reports were excluded for the following reasons: review/narrative design (*n* = 16), absence of biomarkers (*n* = 38), and no extractable data (*n* = 61). A total of 105 studies met all eligibility criteria and were included in the final narrative synthesis. PRISMA, Preferred Reporting Items for Systematic Reviews and Meta‐Analyses.

Study selection followed a structured, multi‐stage screening process. The systematic search, combining terms related to religious and spiritual practices or experiences with terms related to objective neuroimaging and neurophysiological methods as described in Section 2.4, retrieved a total of 450 records: 145 from *Scopus*, 152 from *ProQuest*, and 153 from *PubMed/MEDLINE*. First, duplicate records were removed. Second, titles and abstracts were screened against the eligibility criteria. Third, full texts of potentially eligible studies were retrieved and assessed for final inclusion. Title and abstract screening, full‐text eligibility assessment, and final inclusion decisions were performed independently by two reviewers. Disagreements were resolved through discussion and consensus, and unresolved disagreements were adjudicated by a third reviewer. Reasons for exclusion at the full‐text stage were documented. The PRISMA flow diagram, summarizing record identification, screening, eligibility assessment, and final inclusion, is presented in Figure 1.

### 2.7 Data Extraction

Data extraction was conducted using a standardized and refined framework developed for the present review. Extracted variables included publication details, country or setting, study design, sample size, participant characteristics, religious or spiritual tradition, practice type, contextual classification of the practice, practitioner expertise or prior exposure (where reported), comparator condition, neuroimaging or neurophysiological modality, acquisition paradigm, analytical approach, brain regions or neural systems examined, reported neural effects, direction of effect (where available), and authors’ primary interpretation. For intervention or longitudinal studies, intervention duration, follow‐up period, and pre‐post neural or clinical outcomes were extracted (where available). For molecular imaging studies, the imaging tracer or transporter target was recorded (when reported). For EEG and MEG studies, frequency bands, source‐localization methods, and oscillatory outcomes were extracted (where available).

Data extraction was performed independently by reviewers using a standardized framework, with cross‐checking to enhance accuracy and completeness. Discrepancies in extracted data were resolved through discussion among reviewers. Information presented in tables, figures, or supporting information was extracted and noted as applicable. When studies reported insufficient statistical detail for effect‐size calculation or precise inferential interpretation, this limitation was recorded and considered during synthesis.

### 2.8 Outcomes

The primary outcome of interest was the neural or neurophysiological correlates of religious, spiritual, contemplative, devotional, or ritual practice. These included regional brain activation or deactivation, functional connectivity, resting‐state network changes, EEG or MEG oscillatory activity, cerebral blood flow, structural brain measures, white matter microstructure, cortical thickness, gray matter volume or density, and monoaminergic transporter binding. Secondary outcomes encompassed practice‐specific neural patterns, differences between novice and experienced practitioners, state versus trait effects, and reported associations between neural outcomes and psychological, spiritual, clinical, or phenomenological measures.

### 2.9 Data Synthesis

Given the substantial heterogeneity among included studies concerning religious tradition, practice type, participant population, imaging modality, comparator condition, and outcome reporting, a meta‐analysis was deemed inappropriate. Consequently, findings were synthesized narratively and organized thematically according to practice category and neural system. Practice categories encompassed meditation and contemplative practice, prayer and devotional practice, chanting and recitation, ritual and worship, mystical experience, altered states, and long‐term spiritual or religious practice. Within each category, findings were further interpreted based on modality, direction of neural effect, practitioner expertise, and comparator condition, where such information was available.

The synthesis prioritized identifying convergent and divergent patterns across studies while diligently avoiding overinterpretation of isolated findings. Specific attention was directed toward differentiating activation from deactivation, state effects from trait effects, structural from functional findings, and explicitly religious practices from secular‐derived contemplative interventions. A cross‐traditional synthesis approach was employed to identify neural systems consistently implicated across various practices, including prefrontal, cingulate, default mode, insular, temporoparietal, limbic, hippocampal, reward‐related, and monoaminergic systems. However, conclusions were interpreted with caution because the repeated involvement of a brain region across studies does not necessarily imply functional equivalence across practices.

### 2.10 Assessment of Methodological Limitations

Given the heterogeneity of included studies and the diversity of imaging modalities, a single quantitative risk‐of‐bias tool was not applied across all designs. Instead, methodological limitations were assessed narratively during extraction and synthesis. Considered domains included sample size, study design, comparator condition, blinding where relevant, practitioner expertise characterization, clarity of religious or spiritual framing, control for expectancy and cultural familiarity, imaging modality, statistical reporting, correction for multiple comparisons, and whether conclusions were supported by the design. Particular caution was applied to uncontrolled pre‐post studies, small pilot neuroimaging studies, cross‐sectional comparisons, and studies in which neural outcomes were reported without sufficient inferential statistics or effect‐size information.

### 2.11 Ethics Statement

Because the present review synthesized data from previously published studies and did not involve direct interaction with human participants, the collection of identifiable information, or the generation of new clinical data, ethical approval and informed consent were not required. The review was conducted in accordance with established principles of research integrity, transparency, and responsible reporting.

The authors declare that Antidote Grammar Checker (version 12; https://www.antidote.info) was utilized in the preparation of this manuscript for the following purposes: enhancing clarity of expression, refining grammar and syntax, and improving overall academic writing quality. The AI tool was used iteratively during the revision process to ensure consistency and readability. All substantive contributions to this work—including research design, data analysis, interpretation of results, and scientific conclusions—remain the exclusive responsibility of the authors. The authors reviewed and verified all AI‐generated suggestions and accept full accountability for the final manuscript content. No AI‐generated text was used without author review and approval.

**FIGURE 2:**
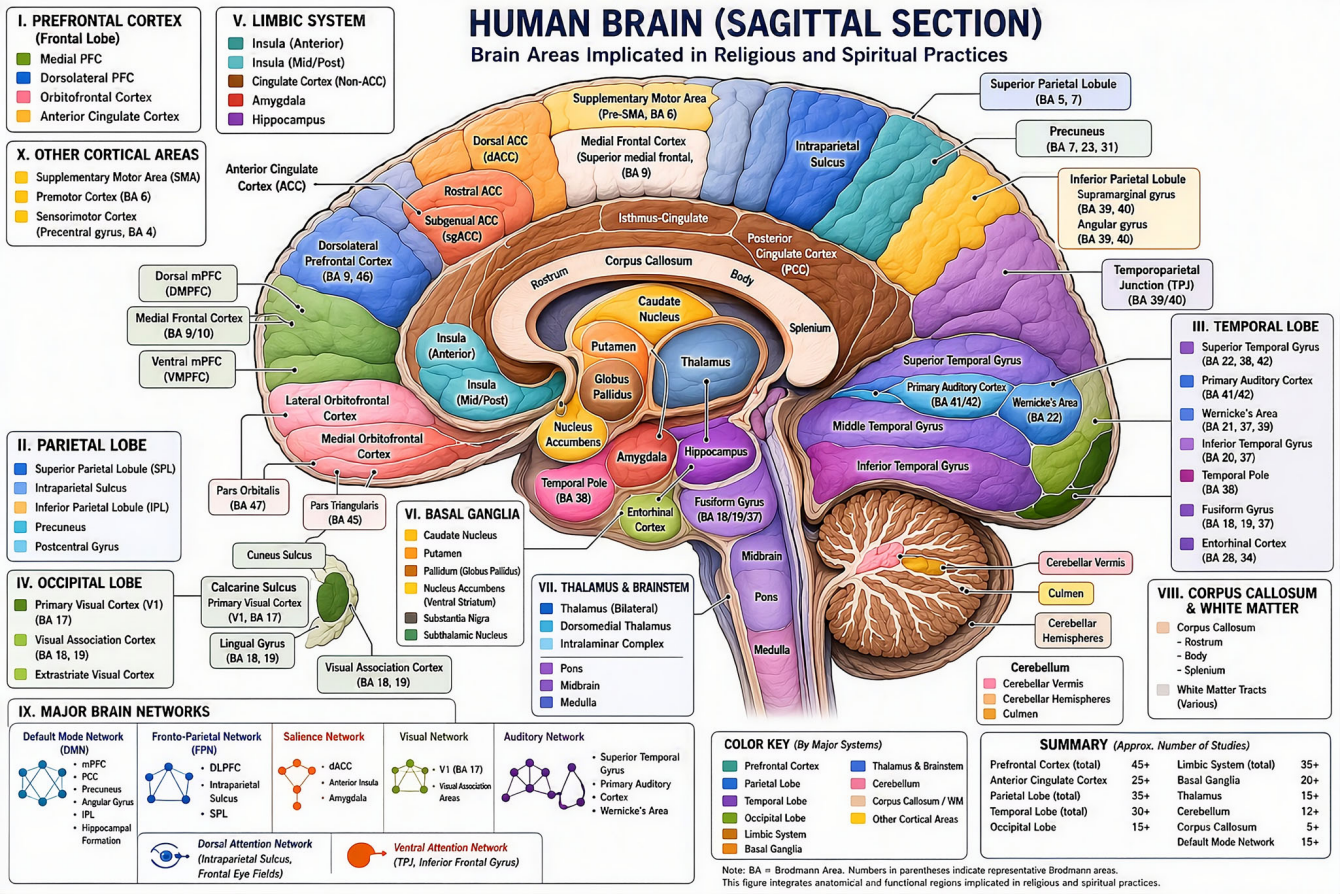
Brain diagram. Schematic mid‐sagittal section of the human brain summarizing the principal cortical, subcortical, and network‐level regions implicated in religious and spiritual practices synthesized across the 105 included studies. Regions are color‐coded by major functional system (prefrontal cortex, parietal lobe, temporal lobe, occipital lobe, limbic system, basal ganglia, thalamus/brainstem, corpus callosum/white matter, and other cortical areas), with representative Brodmann areas (BA) in parentheses. Inset panels depict major functional networks (Default Mode, Fronto‐Parietal, Salience, Visual, Auditory, and Dorsal/Ventral Attention) and an approximate study‐count summary per system. The figure integrates anatomical and functional regions based on neuroimaging evidence and is illustrative rather than a precise anatomical atlas.

Figure 2 was generated using a generative artificial intelligence image‐synthesis model (Claude Opus 4.8), accessed through the institutional account of Dr. Haitham Jahrami, based on the author‐supplied descriptive prompt (reproduced below). The figure is a schematic, illustrative summary and does not constitute a precise anatomical atlas; all region labels, Brodmann‐area assignments, network compositions, and study counts were specified, reviewed, and verified by the authors, who accept full responsibility for its accuracy and content. Exact prompt used is: “A high‐resolution, journal‐quality scientific infographic titled ‘HUMAN BRAIN (SAGITTAL SECTION)’ with subtitle ‘Brain Areas Implicated in Religious and Spiritual Practices’. Central medical illustration of a detailed mid‐sagittal cross‐section of the human brain, anatomically accurate, with color‐coded regions by functional system: PFC, limbic system, temporal lobe, parietal lobe, occipital lobe, basal ganglia, thalamus and brainstem, cerebellum, corpus callosum. Label major structures (DLPFC BA 9/46, medial frontal cortex, pre‐SMA BA 6, ACC subregions [dorsal, rostral, subgenual], corpus callosum [rostrum, body, splenium], caudate nucleus, putamen, globus pallidus, nucleus accumbens, thalamus, anterior and mid/posterior insula, amygdala, hippocampus, entorhinal cortex, fusiform gyrus, temporal pole BA 38, superior/middle/inferior temporal gyri, primary auditory cortex BA 41/42, Wernicke's area BA 22, superior parietal lobule BA 5/7, precuneus BA 7/23/31, inferior parietal lobule [supramarginal and angular gyri BA 39/40], TPJ, posterior cingulate cortex, primary visual cortex V1 BA 17, visual association cortex, midbrain, pons, medulla, cerebellar vermis, cerebellar hemispheres, culmen) with thin leader lines and large legible sans‐serif black text. Include organized legend boxes grouped I–X by system, a bottom ‘Major Brain Networks’ panel (DMN, FPN, Salience, Visual, Auditory, Dorsal Attention, Ventral Attention) as node‐and‐edge diagrams, a color key by major system, and a summary box of approximate study counts per region. Footnote: ‘BA = Brodmann Area. Numbers in parentheses indicate representative Brodmann areas.’ Clean white background, vector‐style medical textbook aesthetic, extra‐large readable typography for print publication.”

## 3 Results

### 3.1 Study Characteristics

The 105 included studies (Supporting Information 3) were published between 1998 and 2026, with 80.0% published after 2009. Represented traditions were Buddhism (24.8%), Christianity (19.0%), Islam (16.2%), mixed or multiple traditions (11.4%), Hinduism (8.6%), Santo Daime (2.9%), Spiritism (1.9%), Sant Mat (1.0%), and unspecified theistic belief (1.0%). The specific religion or denomination of participants was not reported in 13.3% of studies. Median sample size was approximately 23 participants (range 1–211). EEG was the most frequently used modality (49.5% of studies), followed by fMRI (39.0%), structural MRI (10.5%), SPECT (5.7%), MEG (3.8%), PET (1.9%), and DTI (1.0%); a subset of studies additionally incorporated ECG, HRV, GSR, ERP, qPCR, LC/MS/MS, or standardized low‐resolution brain electromagnetic tomography (sLORETA) source analysis alongside a primary modality. A schematic summary of the principal brain regions and functional systems implicated across religious and spiritual practices, based on the synthesized neurotheological evidence, is presented in Figure 2.

### 3.2 Meditation and Contemplative Practice

Thirty‐five studies examined meditation and related contemplative practices, documenting five recurring patterns: (i) modulation of the DMN (the set of brain regions active during mind‐wandering and self‐referential thought, typically quieter during focused tasks); (ii) prefrontal engagement and neural efficiency (changes in the brain's frontal regions responsible for attention and self‐control, which become more efficient with practice); (iii) style‐specific and cross‐traditional oscillatory dynamics (differences in the brain's electrical rhythms that vary by meditation style and appear across different religious traditions); (iv) structural neuroplasticity (physical changes in brain tissue that build up over months or years of sustained practice); and (v) a distinct cluster of findings specific to TM, a mantra‐based technique practiced with eyes closed for fixed periods twice daily.

#### 3.2.1 DMN Modulation and Self‐referential Processing

Experienced mindfulness meditators showed reduced activation in medial PFC and posterior cingulate cortex (PCC) nodes during all meditation types, alongside enhanced functional connectivity between the PCC and dorsolateral PFC, reflecting a trained capacity to detect and disengage from self‐referential mind‐wandering (Brewer et al. 2011). Real‐time fMRI neurofeedback showed that effortless, undistracted awareness consistently produced PCC deactivation, while effortful control produced PCC activation (Garrison et al. 2013). Zen practitioners demonstrated significantly reduced duration of BOLD response to semantic stimuli in default network regions, consistent with cultivated non‐conceptual awareness (Pagnoni et al. 2008). Mindfulness training decoupled interoceptive insular activity from medial prefrontal narrative self‐processing, such that trained participants processed self‐experiences as transient phenomenal events rather than core self‐defining constructs (Farb et al. 2007).

A hierarchical MEG study of three Buddhist selfless‐awareness states found that transitioning to minimal selfhood was marked by high‐gamma suppression in medial PFC, while transitioning to a fully selfless state involved beta‐band suppression across a distributed network including the inferior parietal lobule, thalamus, and PCC/precuneus, with the most experienced meditators showing additional suppression tied to loss of bodily ownership (Dor‐Ziderman et al. 2013). Among experienced Zen meditators, a cognitive‐conflict fMRI paradigm found reduced activation across both DMN and central executive network regions relative to meditation‐naive controls, with inhibitory coupling among PCC, posterior parietal, and lateral parietal regions during meditation, suggesting these networks share attentional resources rather than acting strictly antagonistically (Osaka et al. 2026).

#### 3.2.2 Prefrontal Engagement, Expertise, and Neural Efficiency

Focused attention and open monitoring meditation produced dissociable prefrontal activation patterns among experienced Theravada Buddhist monks, with the ACC and dorsolateral PFC serving antagonistic regulatory roles across styles (Manna et al. 2010). Long‐term expertise was associated with an inverted U‐shaped trajectory. Meditators with approximately 19,000 practice hours showed greater attention‐network activation than novices, whereas those with approximately 44,000 h showed reduced activation, indicating progressive neural efficiency (Brefczynski‐Lewis et al. 2007). Regular meditators showed reduced BOLD activation during a cognitive conflict task relative to non‐meditating controls despite equivalent behavioral performance (Kozasa et al. 2012).

Prayer frequency predicted superior volitional neural self‐regulation, with high‐frequency prayer participants relying on the medial orbitofrontal cortex for effortless attentional gating, paralleling the neural efficiency profile of experienced meditators (Kober et al. 2017). An EEG‐neurofeedback‐derived alpha transformation index, indexing flexible cortical shifting between alpha enhancement and suppression, was significantly greater among Buddhist practitioners than non‐practitioners and correlated with self‐esteem, life satisfaction, and mindfulness scores (Lu et al. 2025). Long‐term compassion meditation strengthened neurovisceral coupling between cardiac activity and the left middle/posterior insula relative to novices, indicating expertise‐dependent refinement of interoceptive‐bodily integration (Lutz et al. 2009).

#### 3.2.3 Style‐Specific and Cross‐Traditional Oscillatory Dynamics

Long‐term Buddhist meditation training induced high‐amplitude gamma oscillations (25–42 Hz) and large‐scale neural phase synchrony during practice, with gamma‐to‐slow‐oscillation ratios correlating positively with cumulative practice hours (*r* = 0.79) and remaining elevated at resting baseline (Lutz et al. 2004). Vipassana practice produced parieto‐occipital gamma augmentation and frontal delta suppression as a trait‐level marker of contemplative expertise (Cahn et al. 2010). Three months of intensive Vipassana reduced the attentional blink and decreased brain‐resource allocation to initial stimuli, indicating more economical attentional distribution (Slagter et al. 2007). Spectral Granger causality analysis of focused attention, open monitoring, and loving‐kindness meditation in monastic Theravada practitioners showed dissociable directed‐connectivity signatures for each style, including a distinctive symmetric bi‐hemispheric beta‐band signature during loving‐kindness meditation associated with compassion and equanimity (Kolev et al. 2026).

Source‐space MEG combined with machine learning showed that both Samatha and Vipassana meditation flattened the aperiodic spectral slope and increased signal complexity relative to rest, although a classifier did not reliably distinguish the two styles except on a criticality‐related measure (Pascarella et al. 2025). Comparing six meditation styles (shamatha, vipassana, zazen, dzogchen, tonglen, and deity visualization) against mind‐wandering, entropy was significantly reduced during meditation regardless of style, while spectral power differences were less consistent, with tonglen and zazen showing the largest alpha increases (Young et al. 2021). Contrasting Theravada (Shamatha, Vipassana) and Vajrayana (Deity and Rigpa) practices, Theravada meditations produced a parasympathetic relaxation response, while Vajrayana meditations produced a sympathetic arousal response with immediate gains in visuospatial performance, challenging the traditional focused‐attention‐versus‐open‐monitoring classification of meditation (Amihai and Kozhevnikov 2014).

A cognitively demanding intuitive‐inquiry practice (Can‐Hua‐Tou) produced stable, trait‐like elevations in beta and gamma power among experienced monks regardless of task condition, contrasting with the alpha‐theta dominance typical of relaxation‐based styles (Lee et al. 2025). A wearable EEG study in a monastic setting found that experienced Zen monks showed higher frontal alpha and theta power than controls during meditation, with no significant difference between meditation and mind‐wandering states, suggesting high continuity between meditative and non‐meditative states in advanced practitioners (Kurek et al. 2025). Zen meditators showed increased EEG signal complexity and occipital beta power relative to controls, with complexity positively correlated with beta and alpha power (Huang and Lo 2009).

Decades‐long Satyananda Yoga teachers showed markedly greater right‐lateralized gamma‐band cortical activity than intermediate students, who instead showed greater low‐frequency activity, consistent with a developmental shift from early sensory withdrawal to later concentrative absorption (Thomas et al. 2014). A related PET study of Yoga Nidra meditation found a 7.9% decrease in striatal D2 (Dopamine D2 receptor) receptor binding, corresponding to an estimated 65% increase in endogenous dopamine release, correlated with increased EEG theta activity (Kjaer et al. 2002). fMRI analysis of the transition into and sustained continuation of meditation identified a cortico‐striato‐thalamo‐cortical “neural switch” mechanism, with onset marked by bilateral putamen and supplementary motor activation and sustained meditation marked by caudate activation and widespread right‐hemisphere white matter deactivation (Baerentsen et al. 2010).

In Juingong meditation, the theta‐to‐alpha ratio was significantly reduced in temporoparietal channels relative to instructed mind‐wandering, with no corresponding change in anterior frontal channels, suggesting a receptive rather than executive attentional mode (Kim et al. 2022). Machine‐learning decoding of self‐reported meditative depth among expert Vipassana practitioners found that combined theta, alpha, and gamma spectral and connectivity patterns achieved above‐chance decoding of subjective depth (Reggente et al. 2025). A direct comparison of Buddhist meditation and Christian prayer found greater central theta amplitude during meditation relative to relaxation, with parietal theta additionally moderated by trait spiritual openness (Dobrakowski et al. 2020).

#### 3.2.4 TM: Neurophysiological Correlates

Using simultaneous MEG and EEG, TM‐induced frontal alpha activity was localized to the medial PFC and ACC, consistent with a state of alert, effortless internal awareness (Yamamoto et al. 2006). Comparing TM to eyes‐closed rest, TM produced significantly lower breath rate and skin conductance, and higher respiratory sinus arrhythmia and EEG alpha coherence within the first minute of practice, supporting a model of TM as a rapidly induced “restfully alert” physiological state (Travis and Wallace 1999). Combining Temporal Experience Tracing with theoretically motivated EEG markers, TM practitioners reported greater intensity and variability of pure awareness than controls, with a double dissociation in which entropy and aperiodic dynamics best distinguished TM from ordinary cognition, while slow‐frequency connectivity best distinguished TM from its own resting baseline (Chandia‐Jorquera et al. 2026).

Long‐term TM practitioners showed 40%–50% fewer pain‐responsive voxels in the thalamus and total brain than controls, and controls who subsequently learned TM showed an equivalent reduction after five months, despite no change in subjective pain intensity, suggesting TM reduces the affective‐motivational rather than sensory‐discriminative dimension of pain (Orme‐Johnson et al. 2006). A small pilot fMRI study comparing brief TM‐style meditation on universal connectedness against digital art and nature videos found greater activation in visual, sensory‐integration, and interoceptive regions during meditation (Krause‐Sorio et al. 2024).

#### 3.2.5 Structural Neuroplasticity

Eight weeks of mindfulness‐based stress reduction produced significant increases in gray matter density in the left hippocampus, PCC, left temporo‐parietal junction, and cerebellum relative to a waitlist control group (Hölzel et al. 2011). Catholic nuns demonstrated increased gray matter volume in the right lingual cortex, left isthmus‐cingulate, posterior cingulate, and several frontal and temporal regions relative to age‐matched lay controls (Chung et al. 2022).

### 3.3 Prayer and Devotional Practice

A total of 21 studies characterized prayer as a neurologically active behavior engaging social cognition networks, prefrontal executive systems, limbic‐reward pathways, autonomic regulation, and, in Islamic prayer, structural white matter organization.

#### 3.3.1 Prayer as Social Cognition

Improvised personal prayer selectively activates the TPJ, temporopolar region, medial PFC, and precuneus (the brain's Theory of Mind network), indicating that sincere devotional address to a perceived divine agent is processed as genuine interpersonal communication (Schjoedt et al. 2009). Formalized prayer, by contrast, activated the dorsolateral PFC and cerebellum, consistent with verbal rehearsal and procedural retrieval. Christian practice consolidated a direct semantic representation of the divine that bypasses episodic retrieval, with connectivity strength inversely correlated with depth of conviction (Ge et al. 2009). Christian belief shifted self‐referential processing from ventral to dorsal medial PFC, reflecting evaluative self‐assessment from a transcendent, God's‐perspective orientation (Han et al. 2008). In contrast, prayer within Sahaja Yoga meditation (drawing on multiple traditions) was associated with widespread deactivation of the bilateral thalamus and left dorsolateral and ventrolateral PFC relative to matched secular speech for both formalized and improvised prayer, suggesting this form of prayer may be neurophenomenologically distinct from prayer relying more heavily on active social‐cognitive processing (Perez‐Diaz et al. 2023).

#### 3.3.2 Belief Modulation, Reward, and Analgesia

Perceived charismatic authority produced significant deactivation of frontal executive networks when Christians listened to speakers believed to possess healing abilities, a belief‐congruent suspension of critical evaluation paralleling hypnotic susceptibility (Schjoedt et al. 2011). Religious prayer reduced pain intensity by 11% and unpleasantness by 26% relative to secular prayer through non‐opioid mechanisms, with reduced BOLD signal in parietofrontal attentional networks (Elmholdt et al. 2017). A comparable mindfulness meditation paradigm produced 40% reductions in pain intensity and 57% reductions in pain unpleasantness, mediated by somatosensory cortex deactivation, anterior cingulate and insula‐mediated reappraisal, and thalamic gating of ascending nociceptive signals (Zeidan et al. 2011). Across four independent task paradigms, devout Mormons’ self‐reported peak spiritual feelings (“feeling the Spirit”) were reproducibly associated with nucleus accumbens activation preceding the subjective peak by one to three seconds, alongside right‐lateralized frontal attentional engagement, implicating classical reward circuitry in the motivational power of religious experience (Ferguson et al. 2018).

#### 3.3.3 Craving and Clinical Recovery Contexts

Prayer within Alcoholics Anonymous’ members engaged self‐referential and DMN areas, with craving ratings inversely correlated with activation in attentional and cognitive control circuitry (Galanter et al. 2017). Healing prayer among participants with major depressive disorder produced increased medial PFC activation and decreased precuneus activity during traumatic memory processing (Baldwin et al. 2016).

#### 3.3.4 Contemplative and Centering Prayer

Contemplative prayer among elderly Catholic sisters was associated with greater posterior‐occipital alpha power relative to rest, with frontal alpha asymmetry becoming more approach‐oriented with age, paralleling signatures reported in mindfulness research (Barcelona et al. 2020). An EEG‐derived Transcendence Index, the balance of theta‐alpha versus beta activity, was significantly higher during prayer than relaxation, correlating positively with theta and low alpha and negatively with high beta and several personality and anxiety measures (Manea et al. 2026). SPECT imaging during centering prayer among Franciscan nuns showed increased regional cerebral blood flow in the PFC and inferior parietal and frontal lobes, with an inverse correlation between prefrontal and ipsilateral superior parietal perfusion, comparable to patterns reported for Tibetan Buddhist visualization meditation (Newberg et al. 2003).

#### 3.3.5 Islamic Prayer (Salat and Dhikr)

Alpha augmentation in parieto‐occipital regions during prostration was equivalent between actual prayer and mimicked conditions, indicating that posture rather than concurrent recitation principally drives EEG relaxation responses (Doufesh et al. 2012). Actual Salat incorporating verbal recitation produced significantly higher gamma power than mimic Salat, with left‐hemispheric dominance consistent with enhanced language network engagement (Doufesh et al. 2016). A multimodal EEG and ECG study demonstrated that Salat increased parieto‐occipital alpha power and parasympathetic HRV while decreasing sympathetic activity (Doufesh et al. 2014). Alpha relative power was significantly higher during Salat than during eyes‐open or eyes‐closed rest, particularly in frontal and parietal regions, increasing progressively over the course of prayer (Khanam et al. 2018).

Statistical shape analysis of the corpus callosum among habitual Islamic prayer practitioners showed significant region‐specific morphological differences from non‐praying controls, concentrated in sub‐regions connecting motor, somatosensory, auditory, and cognitive areas, suggesting use‐dependent white matter remodeling (Baykara et al. 2023). SPECT case‐series data comparing Sufi Dhikr and salat practitioners found both surrender‐based prayer forms decreased cerebral blood flow in prefrontal, parietal, and visual cortices relative to baseline, distinct from the increased frontal activation typical of concentrative meditation, while the more intensely surrendered salat additionally activated the caudate, insula, and thalamus (Newberg et al. 2015).

#### 3.3.6 Musical and Christian Worship

EEG microstate analysis during Christian musical worship identified that the salience network showed the strongest negative association with subjective religious experience, with the auditory network positively associated and the DMN also negatively associated, suggesting salience‐monitoring suppression underlies self‐transcendent absorption (Walter and Koenig 2022). A companion spectral analysis found that religious songs produced greater neurophysiological activation than secular songs, an effect strengthened by self‐selection, with higher self‐reported religious experience correlating with occipital theta and alpha activity resembling meditative states, pointing toward a novel ‘occipital involvement hypothesis’ for worship (Walter 2024).

### 3.4 Chanting, Recitation, and Ritual Practice

A total of 25 studies examined chanting, recitation, and structured ritual practice across Buddhist, Hindu, Islamic, and Christian traditions.

#### 3.4.1 Buddhist Devotional Chanting

Buddhist devotional chanting selectively reduced the late positive potential amplitude during fear‐inducing stimuli, attenuating late‐stage emotional elaboration without affecting early perceptual processing (Gao et al. 2017). A multimodal fMRI and structural MRI study showed that chanting activated the fusiform gyrus, PFC, thalamus, amygdala, midbrain, and cerebellum, with long‐term practitioners showing rightward subcortical structural asymmetry (Gao et al. 2020). Religious chanting of Amitabha Buddha's name produced the largest eigenvector centrality decrease in the PCC alongside increased delta‐band power localized to the same region (Gao et al. 2019), and religious chanting more broadly produced stronger PCC centrality decreases than non‐religious chanting, alongside higher delta power and reduced HRV (Sik et al. 2024).

#### 3.4.2 OM and Mantra Chanting

OM chanting produced bilateral deactivation of the orbitofrontal co rtex, ACC, parahippocampal gyri, thalami, hippocampi, and right amygdala, analogous to vagus nerve stimulation (Kalyani et al. 2011). Re‐analysis of this dataset using Granger causality confirmed that OM chanting specifically reduced outgoing connectivity from the insula, ACC, and orbitofrontal cortex to the amygdala, implicating targeted modulation of emotion‐regulation circuitry (Rao et al. 2018). A single 30‐min session of loud OM chanting among naive practitioners was sufficient to produce a significant whole‐brain increase in relative theta power (Harne and Hiwale 2018). High‐density qEEG with sLORETA source localization found that both verbally chanted and passively heard OM activated overlapping nodes of the frontoparietal control, attentional, and DMNs, with listening additionally engaging orbitofrontal and limbic regions not seen during active chanting (Saini et al. 2024).

Thirty minutes of OM chanting produced a 34.2% increase in relative alpha power, a 42.3% increase in relative theta power, and an 85.4% improvement in the theta‐to‐beta ratio, alongside increased inter‐hemispheric synchronization and signal complexity (Kanwade et al. 2025). Maha Mantra chanting produced significant increases in central and parietal alpha relative power alongside elevated theta and delta activity (Mohanty et al. 2024). Long‐term Gayatri Mantra practitioners showed significantly reduced late positive potential amplitudes to negative emotional images at centroparietal sites, alongside higher emotional competence and non‐attachment scores (Sharma et al. 2025).

#### 3.4.3 Quranic Recitation and Listening

Simultaneous MEG and EEG during rhythmic Quranic recitation showed a broad gamma‐band network spanning language, musical, emotional, memory, and multisensory regions among both Muslim and Arabic‐naive non‐Muslim listeners, with comparable overall engagement between groups (Ab Aziz et al. 2026). fMRI showed that Quranic recitation activated empathic, mentalizing, and episodic memory regions significantly more among Muslim than non‐Muslim participants, indicating activation is mediated by religious meaning rather than acoustic properties alone (AlMahrouqi and Mostafa 2023).

Listening to the Quran's Fatihah chapter produced significant left‐occipital beta desynchronization not seen during Arabic news listening, attributed to enhanced visual mental imagery (Ismail et al. 2022). A related clustering analysis of the same recitation found broader beta desynchronization across frontal, temporal, parietal, and occipital channels than for Arabic news, implicating verbal fluency, memory retrieval, and inhibitory control networks (Samhani et al. 2022). Among diabetic patients, Surah Al‐Rehman recitation reduced systolic blood pressure and stress index while increasing low‐frequency HRV and right prefrontal alpha power (Majeed et al. 2022). Listening to Quranic recitation produced greater alpha‐band inter‐hemispheric brainwave balancing than listening to classical music (Zulkurnaini et al. 2012), and a small EEG/ECG comparison against hard music similarly found greater relaxation indices and smoother cardiac signals during recitation (Taha Alshaikhli et al. 2014).

#### 3.4.4 Dhikr (Islamic Remembrance Practice)

Comparing Dhikr, body‐scan meditation, and theistic contemplative thought against rest, all three practices produced highly similar activation patterns dominated by the caudate, ACC, temporal pole, and supramarginal gyrus, and all three unexpectedly increased rather than decreased DMN activity relative to rest, suggesting shared cultural‐religious framing rather than specific ritual form principally shapes the neural response (Saraei et al. 2023). EEG recordings during two forms of Dhikr found delta amplitude consistently highest among all recorded bands regardless of the specific phrase recited, consistent with a generalized calming effect (Razak et al. 2021). Brief Istighfar Dhikr recitation increased alpha and beta amplitude following induced anxiety, with a gender‐based dissociation in which alpha changes were significant among males and beta changes were significant among females (Sumarti et al. 2024). A case study of Dhikr practice by an adolescent with comorbid epilepsy and depression documented resolution of EEG seizure‐related changes under stress provocation alongside improved depressive symptoms (Sulianti et al. 2018).

#### 3.4.5 Biblical Recitation and Lifelong Scriptural Memorization

Recitation of Psalm 23 activated a frontal‐parietal circuit including bilateral dorsolateral PFC and right medial parietal cortex without activating any limbic regions, supporting a cognitive‐attributional model of scripture‐based religious experience (Azari et al. 2001). Hindu Vedic priests trained from childhood in lifelong oral memorization and recitation of over 10,000 Sanskrit stanzas showed significantly greater cortical thickness than matched controls, localized to the left orbitofrontal cortex and right inferior/middle temporal gyrus, with no hippocampal differences, indicating focal, expertise‐driven structural plasticity (Kalamangalam 2014).

#### 3.4.6 Structured Spiritual Retreat

A seven‐day Ignatian spiritual retreat produced increased resting‐state connectivity between the PCC and right superior frontal gyrus and left pallidum, alongside decreased cerebellar‐hippocampal connectivity (Wintering et al. 2021). The same retreat type produced significant decreases in dopamine transporter binding in the basal ganglia and serotonin transporter binding in the midbrain, with striatal reductions correlating with increases in self‐transcendence scores (Newberg et al. 2018).

### 3.5 Mental Health Outcomes: Depression, Anxiety, and Stress

A total of 14 studies directly examined depression, anxiety, or stress‐related outcomes alongside neural or neuroendocrine correlates of religious and spiritual practice, most converging on prefrontal‐limbic and reward circuitry already implicated above.

#### 3.5.1 Depression

An eight‐week cognition‐and‐behavior‐integrated loving‐kindness meditation intervention significantly reduced depression scores among patients with major depressive disorder relative to controls, with multimodal neuroimaging showing a dissociation in reward‐related activation: reduced frontal‐striatal engagement during reward outcomes but increased engagement during loss outcomes and anticipation following wins, alongside increased resting‐state activity and gray matter in frontal cortex (Z. Chen et al. 2025). A brief course of healing prayer ina participants with major depressive disorder produced sustained symptom improvement alongside increased medial PFC and left inferior frontal gyrus activation and decreased precuneus activation during traumatic memory processing, although this single‐arm, unblinded pilot design warrants cautious interpretation (Baldwin et al. 2016).

Altruism and “love of neighbor as self” spiritual phenotypes were associated with greater cortical thickness across the ventral frontotemporal network, which selectively protected against depression onset among individuals at high familial risk, whereas contemplative practice and general religious commitment alone showed no such association (Miller et al. 2021). Among offspring at high familial risk for depression, high self‐rated importance of religion and spirituality was associated with substantially attenuated white matter microstructural differences in the precuneus, frontal, and temporal lobes that otherwise characterized risk (Li et al. 2019). A parallel resting‐state fMRI study found that greater importance of religion and spirituality was associated with reduced left lateral parietal DMN connectivity specifically among high‐familial‐risk individuals, a normalizing pattern not seen among low‐risk participants (Svob et al. 2016). Four weeks of routine Dhikr practice by an adolescent with comorbid epilepsy and severe depression was associated with a sharp reduction in depression severity alongside decreased seizure frequency (Sulianti et al. 2018).

#### 3.5.2 Anxiety and Religious/Spiritual Coping

Using resting‐state fMRI and the Triple Network Model among older adults with and without mood disorders, greater negative religious coping was associated with increased within‐network connectivity in the cingulo‐opercular salience network (left insula), independent of depression severity, while both positive and negative coping were associated with greater DMN‐central executive network anti‐correlation, reflecting cognitive effort regardless of coping valence (Rosmarin et al. 2024). Positive religious coping scores were correlated with elevated right‐hemisphere theta power in frontal and temporal regions after controlling for general coping strategies, a pattern linked to the known protective association between right frontal‐temporal activity and resistance to depression and PTSD (Imperatori et al. 2020). Long‐term Sant Mat meditators (combining loving‐kindness meditation with a restrictive lifestyle) showed significantly lower state and trait anxiety than novices, with amygdala reactivity during explicit fear processing mediating the relationship between meditation experience and reduced trait anxiety (C. Chen et al. 2018).

#### 3.5.3 Stress, Neuroendocrine Regulation, and Well‐Being

A three‐day mindfulness‐based training program reduced stress‐related functional connectivity between the amygdala and subgenual ACC relative to a matched relaxation control, with reductions predicting lower hair cortisone at four‐month follow‐up, indicating attenuated cumulative hypothalamic‐pituitary‐adrenal‐axis activation (Taren et al. 2014). A structured spiritual practice (guided energy transfer) produced increased precuneus/PCC connectivity within the DMN and increased frontal lobe perfusion alongside significant subjective improvements in mood, calm, and reduced anxiety, outcomes dissociable from a physical‐exercise control group (Gupta et al. 2018). A randomized controlled trial of workplace TM practice found significant increases in EEG Brain Integration Scale scores and significant reductions across total mood disturbance, anxiety, anger, depression, fatigue, and confusion, with large effect sizes and high compliance (Travis et al. 2018).

Three months of TM practice among previously naive meditators reduced a composite depression‐anxiety‐stress score, with this improvement inversely correlated with increased functional connectivity between the PCC and posterior DMN regions (Avvenuti et al. 2020). Comparing long‐term TM practitioners and controls across gene expression, EEG, and hair glucocorticoid measures, older TM practitioners showed shorter ERP latencies and higher Brain Integration Scale scores comparable to young controls, downregulation of multiple aging‐associated genes, and lower hair cortisol and cortisol‐to‐cortisone ratios, jointly suggesting reduced allostatic load with long‐term practice (Wenuganen et al. 2025). Similar anxiolytic and stress‐modulating effects were reported for Quranic recitation among diabetic patients (Majeed et al. 2022), Istighfar Dhikr following induced anxiety (Sumarti et al. 2024), and Maha Mantra chanting (Mohanty et al. 2024), reinforcing convergence between recitation‐based practices and the affective circuitry described above.

### 3.6 Mystical Experience, Altered States, and Mediumship

A total of 11 studies examined mystical union, near‐death imagery, glossolalia, psychedelic ritual, and mediumistic trance as distinct altered states of consciousness.

Mystical union with God among Carmelite nuns engaged a distributed network encompassing the right medial orbitofrontal cortex, right middle temporal cortex, bilateral parietal lobules, bilateral caudate, left ACC, left insula, and extrastriate visual cortex (Beauregard and Paquette 2006). Meditative re‐engagement with near‐death experience imagery activated a similarly broad network spanning the brainstem, orbitofrontal and medial PFC, superior parietal lobule, occipital and temporal cortices, insula, parahippocampal gyrus, and substantia nigra, alongside multiband EEG augmentation, consistent with heightened, integrated processing of reward, self‐referential imagery, and autobiographical memory systems (Beauregard et al. 2009).

Personalized guided‐imagery paradigms comparing spiritual, stressful, and neutral relaxing conditions found that spiritual imagery engaged a ventral frontotemporal network resembling the ventral attention network plus striatum, thalamus, and brainstem, which remained positively engaged throughout the task, unlike the stress and neutral conditions, with engagement correlating with self‐reported spirituality (McClintock et al. 2019). Region‐based analysis of the same dataset additionally found reduced left inferior parietal lobule activity during spiritual relative to neutral imagery and reduced medial thalamus and striatal activity relative to stress imagery, interpreted as reflecting altered self‐other representation during spiritual experience (Miller et al. 2019). Glossolalia was characterized by bilateral prefrontal hypoactivation and left superior parietal augmentation relative to religious singing, reflecting a dissociative state in which verbal production is experienced as externally initiated (Newberg et al. 2006). Zen practitioners’ perception of “inner light” during meditation was reliably indexed by EEG alpha blocking and a shift to beta‐dominant or flattened signals, a pattern absent in non‐meditating controls (Lo et al. 2003).

Ritualistic ayahuasca consumption within the Santo Daime tradition produced systematic reorganization of functional connectome architecture, increasing network entropy and local integration while reducing global efficiency, with the DMN assuming a more central identity‐anchoring role even as individual connectivity fingerprints became less distinct, consistent with the entropic brain hypothesis and offering a candidate mechanism for the co‐occurrence of ego dissolution with preserved biographical self‐awareness (Mallaroni et al. 2024; Viol et al. 2017). Earlier EEG work on ayahuasca similarly documented significant increases in posterior 40 Hz gamma power, interpreted as reflecting enhanced thalamocortical synchronization amplifying endogenous visual imagery (Don et al. 1998).

Experienced spiritist mediums exhibited decreased regional cerebral blood flow in regions subserving cognitive monitoring, memory encoding, and language production during psychographic (automatic writing) trance, a pattern inversely associated with the complexity of produced text and interpreted as reflecting automatized, non‐consciously mediated processing (Peres et al. 2012). Using EEG‐based dynamic brain network analysis, mediumistic trance was distinguished from both a pre‐trance baseline and a prayer‐based control condition by increased and left‐lateralized delta, theta, and alpha connectivity, supporting the interpretation of mediumistic trance as a distinct, non‐pathological altered state of consciousness (Plácido et al. 2026).

### 3.7 Cross‐Traditional Convergences and Direction of Effects

Across the included studies, several neural systems appeared repeatedly across religious and spiritual practices, although the direction and interpretation of these effects varied according to practice type, practitioner expertise, measurement modality, and experimental context.

The PFC was among the most frequently implicated regions, appearing across studies of meditation, prayer, chanting, recitation, and mystical experience. In meditation studies, prefrontal activation was most evident during focused attention and cognitive monitoring, particularly among less experienced or intermediate practitioners, whereas highly experienced meditators showed patterns consistent with reduced effort or increased neural efficiency during attentional tasks (Brefczynski‐Lewis et al. 2007; Kober et al. 2017; Kozasa et al. 2012; Manna et al. 2010). This efficiency pattern extended to newer evidence. An EEG‐derived alpha transformation index reflecting flexible frontal cortical control was significantly greater among Buddhist practitioners than non‐practitioners (Lu et al. 2025), and a Stroop‐based fMRI paradigm found reduced dorsolateral PFC activation among Zen monks relative to controls despite equivalent behavioral performance (Osaka et al. 2026).

TM was associated with a distinct prefrontal signature, including medial prefrontal and anterior cingulate alpha generation localized via combined MEG and EEG (Yamamoto et al. 2006), rapid frontal alpha coherence increases within the first minute of practice (Travis and Wallace 1999), and reduced prefrontal pain reactivity independent of subjective pain intensity (Orme‐Johnson et al. 2006). In prayer studies, prefrontal regions were engaged during formalized prayer, religious self‐referential processing, and exposure to perceived charismatic authority, although Schjoedt et al. (2009) reported frontal executive deactivation when believers listened to speakers who were thought to possess healing abilities, suggesting that prefrontal involvement may differ depending on whether the practice requires active cognitive control or belief‐congruent suspension of critical evaluation (Han et al. 2008; Schjoedt et al. 2009, 2011).

This deactivation pattern also characterized prayer within Sahaja Yoga Meditation, which showed reduced left dorsolateral and ventrolateral PFC activity relative to secular speech (Perez‐Diaz et al. 2023); surrender‐based Islamic prayer and Dhikr, which showed decreased prefrontal cerebral blood flow relative to baseline (Newberg et al. 2015); and centering prayer among Franciscan nuns, which instead showed increased prefrontal perfusion alongside inferior parietal and frontal lobe changes (Newberg et al. 2003). Reward‐related prefrontal engagement was evident among devout Mormons’ “feeling the Spirit” experiences, marked by ventromedial PFC activation alongside nucleus accumbens engagement (Ferguson et al. 2018), and in healing prayer for major depressive disorder, which increased medial PFC and left inferior frontal gyrus activation during traumatic memory processing (Baldwin et al. 2016).

Quranic and mantra‐based recitation practices showed convergent prefrontal involvement, including reduced outgoing orbitofrontal connectivity to the amygdala during OM chanting (Rao et al. 2018), increased right prefrontal alpha power during Surah Al‐Rehman recitation (Majeed et al. 2022), and broad inferior frontal gyrus engagement within a gamma‐band language and multisensory network during Quranic recitation (Ab Aziz et al. 2026). Right‐hemisphere frontal theta elevation also characterized positive religious coping (Imperatori et al. 2020). Mystical and dissociative states showed a divergent prefrontal profile. Mystical union among Carmelite nuns and meditative near‐death imagery both engaged orbitofrontal and medial prefrontal regions in the service of reward and self‐referential processing (Beauregard et al. 2009; Beauregard and Paquette 2006), whereas glossolalia was characterized by bilateral prefrontal hypoactivation, consistent with reduced intentional control (Newberg et al. 2006).

The *ACC* was also repeatedly implicated, particularly in practices involving attentional regulation, conflict monitoring, affective salience, and devotional absorption. Meditation studies identified ACC involvement during focused attention and open monitoring practices, with the ACC and dorsolateral PFC serving antagonistic regulatory roles across styles (Manna et al. 2010), while TM was associated with alpha activity localized to medial prefrontal and anterior cingulate sources (Yamamoto et al. 2006). A Granger causality analysis of focused attention, open monitoring, and loving‐kindness meditation found style‐specific directed connectivity patterns implicating frontal and cingulate regions in each practice's distinct emotional and attentional profile (Kolev et al. 2026), and a cognitive‐conflict paradigm in Zen monks found reduced coupling between the ACC‐associated central executive network and the DMN during meditation (Osaka et al. 2026).

ACC involvement was also reported in mystical experiences among Carmelite nuns (Beauregard and Paquette 2006); in OM chanting, which showed deactivation of the ACC and related limbic‐paralimbic regions; and in a re‐analysis using Granger causality confirming that OM chanting specifically reduced outgoing ACC connectivity to the amygdala (Kalyani et al. 2011; Rao et al. 2018). By contrast, Dhikr, body‐scan meditation, and theistic contemplative thought each activated the ACC relative to rest, suggesting that shared cultural‐religious framing rather than specific ritual form drives this engagement (Saraei et al. 2023), and prayer within Sahaja Yoga Meditation showed reduced dorsal ACC activity relative to secular speech (Perez‐Diaz et al. 2023), again indicating that cingulate engagement depends on the affective and doctrinal profile of the specific devotional practice rather than operating as a uniform “prayer” or “chanting” signature.

The *DMN*, particularly the medial PFC and PCC, showed one of the clearest practice‐dependent patterns. Mindfulness and Zen meditation studies generally reported reduced activation or altered responsiveness in DMN regions during meditative absorption, consistent with reduced self‐referential processing and mind‐wandering (Brewer et al. 2011; Garrison et al. 2013; Pagnoni et al. 2008), and this suppression was echoed in Zen monks' inhibitory coupling among the PCC and posterior parietal regions during meditation (Osaka et al. 2026) and in Christian musical worship, where the DMN showed a negative association with subjective religious experience (Walter and Koenig 2022). A hierarchical MEG study of Buddhist selfless‐awareness states further localized this suppression, finding progressive beta‐band attenuation across DMN‐associated regions, including the PCC, precuneus, and inferior parietal lobule, as meditators moved toward a fully selfless state (Dor‐Ziderman et al. 2013).

By contrast, prayer and recovery‐related devotional practices sometimes engaged self‐referential and DMN‐associated regions, as observed among alcoholics. Anonymous prayer and Christian self‐referential processing paradigms (Galanter et al. 2017; Han et al. 2008), and Dhikr, body‐scan meditation, and theistic contemplative thought unexpectedly increased rather than decreased DMN activity relative to rest (Saraei et al. 2023). A seven‐day Ignatian spiritual retreat was associated with altered resting‐state connectivity involving the PCC (Wintering et al. 2021), and DMN connectivity also carried direct mental health relevance: three months of TM reduced a composite depression‐anxiety‐stress score in proportion to increased PCC‐precuneus connectivity within the posterior DMN (Avvenuti et al. 2020), reduced left lateral parietal DMN connectivity was associated with higher religious and spiritual importance specifically among individuals at high familial risk for depression (Svob et al. 2016), and religious coping was associated with altered DMN‐central executive network anti‐correlation regardless of coping valence (Rosmarin et al. 2024).

The *insula* appeared most prominently in studies involving interoception, compassion, mystical experience, and altered self‐processing. Mindfulness training was associated with a shift from narrative self‐processing toward present‐centered experiential processing involving insular regions, while compassion meditation showed differential relationships between insular BOLD signal and cardiac function among expert versus novice practitioners, with long‐term practice strengthening insula‐cardiac coupling specifically (Farb et al. 2007; Lutz et al. 2009). Insular activation was also reported during mystical union among Carmelite nuns, consistent with the role of this region in somatovisceral integration and emotionally salient subjective experience (Beauregard and Paquette 2006).

OM chanting produced insular deactivation alongside reduced outgoing insula‐to‐amygdala connectivity, again implicating this region in the limbic downregulation associated with mantra‐based practice (Kalyani et al. 2011; Rao et al. 2018), whereas more intensely surrendered Islamic prayer activated the insula alongside the caudate and thalamus, indicating that insular engagement in devotional practice can move in either direction depending on the depth of surrender involved (Newberg et al. 2015). A three‐day mindfulness training program reduced stress‐related amygdala‐subgenual ACC connectivity in a manner that predicted lower cortisol output at four‐month follow‐up, a finding that similarly implicates interoceptive and salience‐related circuitry, including the insula, in the stress‐buffering effects of contemplative practice (Taren et al. 2014).

The *TPJ* and related social cognition networks, including the precuneus, were especially prominent in prayer, chanting, and mystical‐experience paradigms. Improvised personal prayer among highly religious Christians activated the TPJ, temporopolar cortex, medial PFC, and precuneus, supporting the interpretation that personal prayer may be processed as communication with an intentional agent rather than as simple verbal rehearsal (Schjoedt et al. 2009), a pattern consistent with Christian belief consolidating a direct semantic representation of the divine that bypasses episodic retrieval via TPJ‐precuneus connectivity (Ge et al. 2009). Quranic recitation activated regions involved in empathic, mentalizing, and episodic memory processes among Muslim participants but not among non‐Muslim participants, suggesting that religious meaning and participant identification with the tradition may shape neural responses beyond acoustic exposure alone (AlMahrouqi and Mostafa 2023), while a broader gamma‐band network spanning language, memory, and multisensory regions was engaged comparably among Muslim and Arabic‐naive non‐Muslim listeners during rhythmic Quranic recitation, indicating that some acoustic and musical features of recitation are processed independently of religious meaning (Ab Aziz et al. 2026).

By contrast, prayer within Sahaja Yoga meditation showed no comparable activation of social‐cognitive regions relative to secular speech, suggesting that TPJ engagement during prayer is not universal but depends on the specific theological framing of the divine relationship (Perez‐Diaz et al. 2023). Personalized spiritual imagery paradigms further implicated the precuneus and inferior parietal lobule, with reduced left inferior parietal lobule activity during spiritual relative to neutral imagery interpreted as reflecting altered self‐other representation (McClintock et al. 2019; Miller et al. 2019). Moreover, mediumistic automatic writing was associated with decreased activity in regions subserving language production and social‐cognitive monitoring, inversely related to the linguistic complexity of the produced text (Peres et al. 2012).

The *amygdala* and broader limbic system showed bidirectional modulation across practices. OM chanting was associated with deactivation of the amygdala, hippocampus, parahippocampal gyri, thalami, orbitofrontal cortex, and ACC, a pattern interpreted as similar to limbic downregulation observed in vagus nerve stimulation paradigms, and a Granger causality re‐analysis confirmed that this effect was driven by specifically reduced outgoing connectivity from the insula, ACC, and orbitofrontal cortex to the amygdala (Kalyani et al. 2011; Rao et al. 2018). Mindfulness‐based and meditation‐related studies also reported changes relevant to amygdala function and stress‐related connectivity, including reduced amygdala‐subgenual ACC connectivity following mindfulness training that predicted lower cortisol output months later (Taren et al. 2014). Long‐term Sant Mat meditators showed reduced trait anxiety mediated specifically by amygdala reactivity during explicit fear processing (C. Chen et al. 2018).

In contrast, Buddhist chanting studies showed activation of regions including the amygdala during chanting paradigms and attenuation of late‐stage emotional responses to fear‐inducing stimuli, indicating that chanting may modulate limbic reactivity rather than uniformly suppress it (Gao et al. 2017, 2020). Islamic devotional practices showed a similarly mixed limbic profile. Surrender‐based Dhikr and Salat decreased cerebral blood flow across sensory and prefrontal regions, while the more intensely surrendered condition additionally activated limbic and reward‐related structures (Newberg et al. 2015). Delta‐band amplitude was consistently elevated during Dhikr regardless of the specific phrase recited (Razak et al. 2021), and Istighfar Dhikr following induced anxiety produced sex‐differentiated alpha and beta changes (Sumarti et al. 2024). Reward‐related limbic engagement was also evident among devout Mormons' spiritual experiences, with nucleus accumbens activation reproducibly preceding the subjective peak of “feeling the Spirit” (Ferguson et al. 2018).

Evidence for *hippocampal* and *structural neuroplasticity* was more limited but clinically relevant, and this evidence base has expanded considerably beyond gray matter volume alone. Mindfulness‐based stress reduction was associated with increased gray matter density in the left hippocampus, PCC, left TPJ, and cerebellum after eight weeks of training (Hölzel et al. 2011). Catholic nuns showed regional gray matter differences in cingulate, frontal, temporal, and occipital regions compared with lay controls, although the cross‐sectional nature of this study precluded causal inference (Chung et al. 2022). DTI evidence also suggested that religious and spiritual importance may moderate white matter microstructural patterns among individuals at familial risk for depression, but these findings should be interpreted as associative rather than causal (Li et al. 2019).

Structural adaptation extended beyond the hippocampus and cortex to white matter tracts directly shaped by devotional posture and practice. Habitual Islamic prayer practitioners showed region‐specific corpus callosum morphological differences concentrated in sub‐regions connecting motor, somatosensory, auditory, and cognitive processing areas, consistent with use‐dependent remodeling (Baykara et al. 2023). Moreover, Hindu Vedic priests trained from childhood in lifelong oral scriptural memorization showed focal cortical thickening in the left orbitofrontal cortex and right inferior/middle temporal gyrus, with no hippocampal differences, indicating expertise‐driven plasticity specific to verbal semantic and prosodic processing rather than episodic memory systems (Kalamangalam 2014). Cortical thickness across the ventral frontotemporal network was additionally associated with altruistic and prosocial spiritual phenotypes and appeared selectively protective against depression onset among individuals at high familial risk (Miller et al. 2021).

Finally, *subcortical dopaminergic and serotonergic systems* were implicated in a smaller subset of molecular imaging studies, several of which carry direct relevance to reward and mood regulation. Yoga Nidra meditation was associated with reduced 11C‐raclopride binding potential in the ventral striatum, interpreted as increased endogenous dopamine release during meditation‐induced altered consciousness (Kjaer et al. 2002). A one‐week spiritual retreat was associated with changes in dopamine transporter binding in the basal ganglia and serotonin transporter binding in the midbrain, with some transporter changes correlating with increases in self‐transcendence (Newberg et al. 2018). Reward circuitry was also directly implicated in devotional experience through nucleus accumbens activation during “feeling the Spirit” among devout Mormons (Ferguson et al. 2018), and long‐term TM practice was associated with molecular markers of reduced allostatic load, including downregulation of several aging‐associated genes and lower hair cortisol and cortisol‐to‐cortisone ratios (Wenuganen et al. 2025).

A structured spiritual practice involving guided energy transfer produced increased frontal lobe perfusion and DMN connectivity alongside subjective mood and anxiety improvements dissociable from a physical‐exercise control condition, suggesting that reward‐ and perfusion‐related changes may also accompany less formally devotional contemplative practices (Gupta et al. 2018). These findings suggest that reward and salience‐related neurotransmitter and neuroendocrine systems may contribute to spiritual and contemplative phenomenology and, in several cases, to measurable stress and mood outcomes. However, the small samples and preliminary nature of many of these studies warrant cautious interpretation.

## 4 Discussion

### 4.1 Main Findings

The present review synthesized evidence from 105 empirical studies spanning 28 years (1998–2026), eight religious and spiritual traditions, and seven neuroimaging and neurophysiological modalities. The principal finding was that religious and spiritual practices produced consistent, practice‐specific, and in many cases neuroplastic modifications to brain structure and function. This conclusion rests not on any single study but on the convergence of findings across methodologically heterogeneous and culturally diverse evidence, extended to include contemplative, devotional, ritual, psychedelic, and dissociative practices, as well as a growing body of studies conducted directly among clinical or at‐risk populations rather than healthy volunteers alone.

Three interpretive errors should be avoided: (i) treating neuroimaging findings as proof of theological claims commits a category error; (ii) dismissing religious descriptions of prayer or contemplation as mere metaphor is untenable once neural, endocrine, and autonomic correlates are documented; and (iii) drawing one‐to‐one mappings between a neural pattern and a devotional category ignores the multidimensional nature of religious experience (Barbour 2000; Whewell 2014). The theoretical framework that best accommodates these findings conceptualizes spiritual practices as complex, experience‐dependent behaviors whose neural effects are shaped by the specific attentional strategy deployed, the emotional and motivational significance of the practice, the depth of subjective surrender or absorption involved, and the cumulative history of engagement.

### 4.2 Practice‐Specific Signatures

Meditation is characterized by a dynamic between DMN suppression and prefrontal‐cingulate engagement that shifts from effortful to effortless as expertise deepens. This dynamic is supported by cross‐traditional evidence showing that formally distinct styles (Focused Attention, Open Monitoring, Loving Kindness, Samatha, Vipassana, and Vajrayana visualization practices) produce dissociable oscillatory and connectivity signatures despite converging on similar entropy and complexity changes (Amihai and Kozhevnikov 2014; Kolev et al. 2026; Pascarella et al. 2025). This DMN‐suppressing capacity has direct mechanistic relevance to the ruminative DMN hyperactivity that is among the most robust neurobiological features of major depressive disorder, a link strengthened by a clinical trial showing that a loving‐kindness intervention normalizes reward‐circuit function and reduces depression severity among diagnosed patients (Z. Chen et al. 2025).

TM constitutes a partially distinct mechanistic cluster within this practice family, marked by reproducible medial prefrontal and anterior cingulate alpha generation (Yamamoto et al. 2006), reduced thalamic pain reactivity independent of subjective pain intensity (Orme‐Johnson et al. 2006), and, with sustained practice, molecular and neuroendocrine correlates of reduced allostatic load, including downregulation of aging‐associated genes and lower cortisol output (Wenuganen et al. 2025).

Prayer engages a fundamentally different neural architecture organized around social cognition. The activation of the TPJ and medial PFC during improvised personal prayer indicates that sincere devotional communication with a perceived divine agent is neurologically equivalent to real interpersonal interaction, providing the neural correlates of companionship and understanding even in solitude (Schjoedt et al. 2009). Notably, this signature is not universal across devotional traditions.

Prayer within Sahaja Yoga meditation instead produces widespread thalamic and prefrontal deactivation rather than heightened theory of mind engagement (Perez‐Diaz et al. 2023), suggesting that the specific theological framing of the divine relationship, rather than prayer as a generic behavioral category, determines which neural systems are activated. Reward circuitry also appears central to some devotional traditions, with nucleus accumbens activation reliably preceding the subjective peak of “feeling the Spirit” across independent task paradigms among devout Mormons (Ferguson et al. 2018), pointing to a motivational, dopaminergic dimension of prayer that complements its social‐cognitive architecture.

Chanting and recitation operate through limbic modulation, gamma‐band and delta‐band oscillatory enhancement, and autonomic nervous system regulation. The finding that OM chanting produces limbic deactivation analogous to vagus nerve stimulation (Kalyani et al. 2011), and that this effect is driven specifically by reduced directional connectivity from the insula, anterior cingulate, and orbitofrontal cortex to the amygdala (Rao et al. 2018), provides a mechanistic pathway through which a historically practiced devotional behavior may produce clinical effects comparable to an approved medical intervention.

Devotional chanting further produces long‐term structural asymmetry in subcortical emotion‐regulation structures (Gao et al. 2020), indicating that its limbic effects are not purely transient. Islamic Dhikr shows convergent but practice‐specific outcomes, including case‐level clinical improvement in comorbid depression and epilepsy (Sulianti et al. 2018) and sex‐differentiated oscillatory responses to induced anxiety (Sumarti et al. 2024). This suggests that repetitive devotional recitation may engage partially sex‐specific regulatory pathways.

Salat is distinctive in combining posture, breath, recitation, and directed attention within a fixed five‐times‐daily rhythm. The parieto‐occipital alpha augmentation, parasympathetic HRV shift, and activation of empathic and mentalizing networks during Quranic recitation (AlMahrouqi and Mostafa 2023; Doufesh et al. 2014) align with evidence that vagally mediated HRV indexes prefrontal regulatory capacity over subcortical threat circuits (Thayer et al. 2012). This embodied, repetitive structure has a direct structural correlate. Habitual Salat practitioners show region‐specific corpus callosum remodeling concentrated in sub‐regions connecting the motor, somatosensory, auditory, and cognitive systems activated during prayer (Baykara et al. 2023), providing morphological evidence that the postural and multisensory demands of Salat leave a durable anatomical signature.

Mystical and altered state experiences, including near‐death imagery, glossolalia, mediumistic trance, and ritual ayahuasca ingestion, activate the most distributed and integrative neural profiles in the entire corpus, engaging simultaneously the networks of positive affect, reward, self‐representation, visual imagery, memory, and somatovisceral integration, consistent with the phenomenological quality of mystical experience as a totalizing alteration of consciousness. The addition of (i) connectome‐level evidence from ayahuasca ritual, showing increased entropy and a shift of identity‐anchoring functions toward the DMN even as individual connectivity fingerprints become less distinct (Mallaroni et al. 2024), and (ii) EEG‐based network evidence distinguishing mediumistic trance from both rest and a prayer control condition (Plácido et al. 2026), extends this profile to dissociative and psychedelic‐ritual states that were absent from earlier syntheses of this literature.

### 4.3 Implications for Mental Health

Most of the 105 reviewed studies examined healthy participants. Therefore, the present review does not establish that spiritual practices treat diagnosed mental health conditions. However, a meaningful subset of the current evidence base was collected directly among clinical or at‐risk populations, including (i) a randomized loving‐kindness meditation trial among patients with major depressive disorder (Z. Chen et al. 2025), (ii) a pilot healing‐prayer intervention among depressed patients with sustained symptom reduction (Baldwin et al. 2016), (iii) a case study of Dhikr practice with an adolescent experiencing comorbid depression and epilepsy (Sulianti et al. 2018), and (iv) religious‐coping studies conducted in older adults with and without diagnosed mood disorders (Imperatori et al. 2020; Rosmarin et al. 2024). This shift strengthens, without yet confirming, the mechanistic account that follows.

DMN hyperactivity, the signature most consistently modified by meditation, is one of the most robust neurobiological features of major depressive disorder, generalized anxiety disorder, and attention‐deficit/hyperactivity disorder. Amygdala hyperreactivity, modulated by chanting, Dhikr, and mindfulness, is the core pathological mechanism in anxiety disorders, PTSD, and specific phobias, and this circuit's clinical relevance is reinforced by direct evidence that a three‐day mindfulness program reduced amygdala‐subgenual ACC connectivity in a manner that predicted lower cortisol output months later (Taren et al. 2014). Hippocampal atrophy, partially reversed by mindfulness‐based stress reduction (Hölzel et al. 2011), is a documented structural consequence of chronic stress.

Dopaminergic and serotonergic signaling, modified by meditation, retreat, and Yoga Nidra (Kjaer et al. 2002; Newberg et al. 2018), contributes to the neurobiology of depression, addiction, and anhedonia, although contemporary models situate these systems within a wider network account (Moncrieff et al. 2023). Long‐term TM practice extends this picture to the molecular level, with downregulation of aging‐associated genes and reduced cortisol‐to‐cortisone ratios suggestive of reduced allostatic load (Wenuganen et al. 2025). White matter and functional connectivity deficits associated with familial risk for depression are attenuated by high religious and spiritual importance, whether indexed structurally (Li et al. 2019) or through DMN connectivity (Svob et al. 2016), and cortical thickness in the ventral frontotemporal network, associated specifically with altruistic and prosocial spiritual phenotypes, appears selectively protective against depression onset among genetically vulnerable individuals (Miller et al. 2021).

The public health implications deserve separate emphasis. The treatment gap in global mental health remains large, particularly in low‐ and middle‐income countries and in communities where mental health stigma is high. Religious and spiritual practices are, in many of these same communities, already ubiquitous, freely available, culturally legitimate, and actively engaged in by a substantial proportion of the population. If these practices produce neurobiologically protective effects in systems implicated in psychopathology, as the evidence reviewed here strongly suggests, and if this protective effect extends even to genetically or environmentally at‐risk populations rather than healthy volunteers alone, they represent a scalable, culturally embedded mental health resource that clinical and public health science has been insufficiently systematic in characterizing.

### 4.4 Limitations and Future Directions

Several methodological limitations should be considered when interpreting these findings. Although the search spanned three electronic databases (*Scopus, PubMed/MEDLINE*, and *ProQuest*, the latter including 23 institutional sub‐databases) and the review protocol was registered on the OSF, screening was restricted to English‐language, peer‐reviewed empirical studies, which may have excluded relevant non‐English literature, gray literature, and dissertations not indexed within these platforms. The substantial heterogeneity across religious traditions, practice definitions, participant populations, imaging modalities, comparator conditions, and outcome reporting precluded quantitative meta‐analysis. The narrative, SWiM‐guided synthesis adopted here identified convergent and divergent patterns but could not provide pooled effect‐size estimates or formal heterogeneity statistics.

Future research should prioritize multi‐tradition comparative neuroimaging studies comparing formally equivalent practice types across Buddhist, Christian, Islamic, and Hindu practitioners using identical protocols. There should also be clinical neuroimaging trials among populations with diagnosed mental health disorders using rigorous randomized controlled designs, as well as expanded investigation of systematically underrepresented traditions, including Jewish contemplative prayer, additional Sufi and Sant Mat lineages, and indigenous spiritual practices. Also needed are multimodal concurrent neuroimaging studies combining fMRI with EEG, ECG, and peripheral physiological measures. Trials should test devotional practices within their own cultural contexts rather than as secularized protocols, with Salat, Dhikr, contemplative prayer, and loving‐kindness practice meriting pre‐registered randomized trials as adjuncts to standard care for anxiety, insomnia, depression, and PTSD.

## 5 Conclusion

The present systematic review, conducted in accordance with PRISMA 2020 guidelines and registered on the OSF, provides the most comprehensive synthesis to date of empirical neuroimaging and neurophysiological evidence on the effects of religious and spiritual practices on the human nervous system. Across 105 studies identified through a systematic search of three electronic platforms (25 databases), spanning eight religious and spiritual traditions, seven neuroimaging and neurophysiological modalities, and 28 years of research, a consistent and theoretically coherent picture emerged. Religious and spiritual practices are neurologically active behaviors that engage specific, identifiable, and clinically relevant brain systems in ways that are practice‐specific, tradition‐sensitive, and neuroplastically cumulative.

The PFC, ACC, DMN, insula, amygdala, hippocampus, TPJ, and subcortical dopaminergic and serotonergic systems are the principal neural substrates through which diverse spiritual practices exert their effects. Meditation operates primarily through DMN suppression and progressive prefrontal efficiency, with distinguishable sub‐signatures across contemplative styles and a molecularly and neuroendocrinologically distinct profile for TM specifically. Prayer engages social cognition networks in a manner equivalent to real interpersonal communication, although this signature varies by theological framing and is complemented by reward‐circuit engagement in some traditions.

Chanting and repetitive recitation produce limbic modulation, gamma‐ and delta‐band enhancement, and, with sustained practice, structural asymmetry in emotion‐regulation circuitry. Salat integrates posture, recitation, and attention into a rhythmically repeated practice with both functional and structural neural correlates. Mystical, dissociative, and psychedelic‐ritual states activate the most distributed and integrative neural profiles observed in the extant literature. Long‐term practice across nearly all traditions produces structural neuroplasticity in regions governing memory, emotional regulation, attentional control, and prosocial cognition.

These findings are mechanistically relevant to mental health in a manner that is scientifically specific and theoretically grounded. The circuits modified by spiritual practice, including the DMN, the amygdala and its prefrontal and subgenual cingulate connections, the hippocampus, and dopaminergic and serotonergic reward systems, are precisely the circuits whose dysregulation constitutes the neuropathology of depression, anxiety, PTSD, and addiction. A growing, although still limited, number of studies conducted directly in clinical and at‐risk populations now supports this mechanistic account rather than resting solely on inference from healthy volunteers.

Given the substantial heterogeneity documented across traditions, practices, and methodologies, the review's synthesis was necessarily narrative rather than quantitative, and its conclusions should be read as identifying convergent mechanistic patterns rather than pooled effect estimates. The review does not claim that spiritual practices treat these conditions. It demonstrates that spiritual practices engage their neural substrates in directions that are biologically protective and therapeutically relevant. Neurotheology is the scientific study of one of the most universally practiced categories of human behavior and one of the most neurobiologically consequential, and the evidence reviewed here establishes it as an empirically grounded, methodologically rigorous, and clinically significant field whose further development deserves sustained investment of the neuroscientific, psychological, and medical communities.

## Author Contributions

Conceptualization: Waqar Husain and Haitham Jahrami. Methodology: Waqar Husain and Haitham Jahrami. Data collection, Waqar Husain, Haitham Jahrami, Urwa tul Wausqa, Maha Farooq, and Zehra Tariq. Software, Waqar Husain and Haitham Jahrami. Formal analysis, Waqar Husain and Haitham Jahrami. Writing – Original draft preparation, Waqar Husain, Urwa tul Wausqa, Maha Farooq, Zehra Tariq, Achraf Ammar, Khaled Trabelsi, Mark D. Griffiths, Amir Pakpour, and Haitham Jahrami. Writing – review and editing, Waqar Husain, Urwa tul Wausqa, Maha Farooq, Zehra Tariq, Achraf Ammar, Khaled Trabelsi, Mark D. Griffiths, Amir Pakpour, and Haitham Jahrami. Funding acquisition, not applicable.

## Ethics Statement

The authors have nothing to report.

## Funding

The authors have nothing to report.

## Conflicts of Interest

The authors declare no conflicts of interest.

## Supporting information




**Supplementary Material**: brb371733‐sup‐0001‐SuppMat.docx

**Supplementary Material**: brb371733‐sup‐0002‐SuppMat.docx

**Supplementary Material**: brb371733‐sup‐0003‐SuppMat.docx

## Data Availability

No new primary datasets were generated. Extracted study‐level data are available in Supporting Information 3. To enhance transparency, reproducibility, and methodological accountability, the review protocol and analytical framework were registered on the Open Science Framework (OSF; https://osf.io/wm6at/overview).
