# Correction to “The Neuroscience of Religious and Spiritual Practices: A Systematic Review of Neurotheological Evidence”

**DOI:** 10.1002/brb3.71785

**Published:** 2026-09-17

Husain, W., A. Ammar, U. T. Wusqa, et al. 2026. “The Neuroscience of Religious and Spiritual Practices: A Systematic Review of Neurotheological Evidence.” *Brain and Behavior* 16, no. 9: e71733. https://doi.org/10.1002/brb3.71733. PMID: 42670176; PMCID: PMC13527413.

The authors identified visual inaccuracies in Figure 2, which resulted from the AI‐assisted image‐generation process. Although the figure was intended as a conceptual schematic rather than a precise anatomical atlas, certain anatomical representations, spatial relationships, and labels were visually misleading. Specifically, the published Figure 2 included lateral cortical structures not visible in a true midsagittal section, used the nonstandard anatomical label “Cuneus Sulcus,” and incorrectly placed the label for the isthmus of the cingulate gyrus. Consequently, Figure 2 and its caption should be removed. Additionally, the third paragraph in Section 2.11 and the last sentence in Section 3.1 should be removed.

This figure was purely illustrative and was not utilized in the study selection, data extraction, analysis, or interpretation of findings. Its removal therefore does not impact the reported results, discussion, conclusions, or the overall scientific validity of the systematic review.

We apologize for this error.

