# Supplementary material for: The Neuroscience of Religious and Spiritual Practices: A Systematic Review of Neurotheological Evidence
Source: Brain Behav. 2026 Aug 30;16(9):e71733. doi: 10.1002/brb3.71733 (PMC13527413; doi:10.1002/brb3.71733)
Supplement: Supplementary file 1 [file BRB3-16-e71733-s001.docx]

**Supplementary Material 1: Database-specific search strategies**

A systematic literature search was conducted in three electronic database platforms: Scopus, PubMed/MEDLINE, and ProQuest, from database inception to 30 June 2026. Within ProQuest, the search was conducted across 23 available institutional sub-databases and collections, including Academic Video Online, American Periodicals, British Periodicals, Coronavirus Research Database, Ebook Central, Education Magazine Archive, Education Research Index (1966–current), Health & Medical Collection, MEDLINE (1946–current), Periodicals Archive Online, ProQuest Dissertations & Theses Global, ProQuest One Health & Nursing (1946–current), British Nursing Collection (1994–current), Health & Medical Research Collection (1946–current), ComDisDome (1950–current), Consumer Health Database, Healthcare Administration Database, Medical Database, Public Health Database, Sports Medicine & Education Index (1970–current), Nursing & Allied Health Collection, Publicly Available Content Database, and The Vogue Archive. Search strategies were adapted to the syntax of each database and combined terms related to religious and spiritual practices or experiences, including neurotheology, neuroscience of religion, prayer, Salat/Salah, dhikr/zikr, Quranic recitation, religious chanting, glossolalia, worship, mystical experience, spiritual retreat, self-transcendence, and tradition-specific contemplative practices, with terms related to objective neuroimaging and neurophysiological modalities, including fMRI, structural MRI, resting-state fMRI, EEG, MEG, PET, SPECT, DTI, functional connectivity, BOLD signal, cerebral blood flow, grey/gray matter, white matter, cortical thickness, and monoaminergic transporter imaging. Searches were restricted during screening to English-language, peer-reviewed empirical human studies using objective neurological measurement instruments. Reviews, commentaries, theoretical papers, dissertations, theses, books, book chapters, magazines, newspapers, videos, archival non-journal materials, animal studies, and records without objective neuroimaging or neurophysiological outcomes were excluded from the eligible study pool.

**Scopus**

TITLE-ABS-KEY(

neurotheology

OR "neuroscience of religion"

OR "spiritual neuroscience"

OR "religious experience"

OR "spiritual experience"

OR "mystical experience"

OR "religious practice"

OR "spiritual practice"

OR "personal prayer"

OR "formal prayer"

OR "Christian prayer"

OR "Islamic prayer"

OR "Muslim prayer"

OR salat

OR salah

OR dhikr

OR zikr

OR "Quran recitation"

OR "Quranic recitation"

OR "religious chanting"

OR "devotional chanting"

OR glossolalia

OR "speaking in tongues"

OR "spiritual retreat"

OR worship

)

AND

TITLE-ABS-KEY(

fMRI

OR "functional magnetic resonance imaging"

OR EEG

OR electroencephalograph*

OR MEG

OR magnetoencephalograph*

OR PET

OR "positron emission tomography"

OR SPECT

OR "single photon emission computed tomography"

OR DTI

OR "diffusion tensor imaging"

OR "structural MRI"

OR "functional connectivity"

OR "cerebral blood flow"

OR BOLD

OR "gray matter"

OR "grey matter"

OR "cortical thickness"

OR "dopamine transporter"

OR "serotonin transporter"

)

AND

TITLE-ABS-KEY(

brain

OR neural

OR neuroimaging

OR neurophysiolog*

OR electrophysiolog*

)

AND NOT

TITLE-ABS-KEY(

animal*

OR rat

OR rats

OR mouse

OR mice

OR rodent*

)

**PubMed/Medline**

(

neurotheology[Title/Abstract]

OR "neuroscience of religion"[Title/Abstract]

OR "spiritual neuroscience"[Title/Abstract]

OR "religious neuroscience"[Title/Abstract]

OR "religious experience"[Title/Abstract]

OR "spiritual experience"[Title/Abstract]

OR "mystical experience"[Title/Abstract]

OR "religious practice"[Title/Abstract]

OR "spiritual practice"[Title/Abstract]

OR "religious belief"[Title/Abstract]

OR "spiritual belief"[Title/Abstract]

OR "religious cognition"[Title/Abstract]

OR "religious ritual"[Title/Abstract]

OR "spiritual ritual"[Title/Abstract]

OR "religious behavior"[Title/Abstract]

OR "spiritual behavior"[Title/Abstract]

OR prayer[Title/Abstract]

OR praying[Title/Abstract]

OR "personal prayer"[Title/Abstract]

OR "formal prayer"[Title/Abstract]

OR "intercessory prayer"[Title/Abstract]

OR "contemplative prayer"[Title/Abstract]

OR "centering prayer"[Title/Abstract]

OR "Christian prayer"[Title/Abstract]

OR "Islamic prayer"[Title/Abstract]

OR "Muslim prayer"[Title/Abstract]

OR salat[Title/Abstract]

OR salah[Title/Abstract]

OR dhikr[Title/Abstract]

OR zikr[Title/Abstract]

OR "Quran recitation"[Title/Abstract]

OR "Quranic recitation"[Title/Abstract]

OR "scripture recitation"[Title/Abstract]

OR "religious recitation"[Title/Abstract]

OR "religious chanting"[Title/Abstract]

OR "devotional chanting"[Title/Abstract]

OR mantra[Title/Abstract]

OR "OM chanting"[Title/Abstract]

OR glossolalia[Title/Abstract]

OR "speaking in tongues"[Title/Abstract]

OR "spiritual retreat"[Title/Abstract]

OR worship[Title/Abstract]

OR "religious worship"[Title/Abstract]

OR "religious coping"[Title/Abstract]

OR "self-transcendence"[Title/Abstract]

OR "transcendent experience"[Title/Abstract]

)

AND

(

fMRI[Title/Abstract]

OR "functional magnetic resonance imaging"[Title/Abstract]

OR MRI[Title/Abstract]

OR "magnetic resonance imaging"[Title/Abstract]

OR EEG[Title/Abstract]

OR electroencephalograph*[Title/Abstract]

OR MEG[Title/Abstract]

OR magnetoencephalograph*[Title/Abstract]

OR PET[Title/Abstract]

OR "positron emission tomography"[Title/Abstract]

OR SPECT[Title/Abstract]

OR "single photon emission computed tomography"[Title/Abstract]

OR DTI[Title/Abstract]

OR "diffusion tensor imaging"[Title/Abstract]

OR "structural MRI"[Title/Abstract]

OR "resting-state fMRI"[Title/Abstract]

OR "functional connectivity"[Title/Abstract]

OR "cerebral blood flow"[Title/Abstract]

OR BOLD[Title/Abstract]

OR "gray matter"[Title/Abstract]

OR "grey matter"[Title/Abstract]

OR "white matter"[Title/Abstract]

OR "cortical thickness"[Title/Abstract]

OR "brain volume"[Title/Abstract]

OR "regional brain volume"[Title/Abstract]

OR "neural correlates"[Title/Abstract]

OR "brain activity"[Title/Abstract]

OR "brain activation"[Title/Abstract]

OR "dopamine transporter"[Title/Abstract]

OR "serotonin transporter"[Title/Abstract]

)

AND

(

brain[Title/Abstract]

OR neural[Title/Abstract]

OR cortical[Title/Abstract]

OR neuroimaging[Title/Abstract]

OR neurophysiolog*[Title/Abstract]

OR electrophysiolog*[Title/Abstract]

OR "default mode network"[Title/Abstract]

OR "medial prefrontal cortex"[Title/Abstract]

OR "prefrontal cortex"[Title/Abstract]

OR "anterior cingulate"[Title/Abstract]

OR insula[Title/Abstract]

OR amygdala[Title/Abstract]

OR hippocampus[Title/Abstract]

OR "temporoparietal junction"[Title/Abstract]

)

NOT

(

animal*[Title/Abstract]

OR rat[Title/Abstract]

OR rats[Title/Abstract]

OR mouse[Title/Abstract]

OR mice[Title/Abstract]

OR rodent*[Title/Abstract]

)

**ProQuest**

AB,TI(

"Buddhist meditation"

OR "Zen meditation"

OR Vipassana

OR Samatha

OR "loving-kindness meditation"

OR "compassion meditation"

OR "Transcendental Meditation"

OR "Yoga Nidra"

OR "OM chanting"

)

AND

AB,TI(

fMRI

OR "functional magnetic resonance imaging"

OR EEG

OR electroencephalograph*

OR MEG

OR magnetoencephalograph*

OR PET

OR SPECT

OR DTI

OR "diffusion tensor imaging"

OR "functional connectivity"

OR BOLD

OR "gray matter"

OR "grey matter"

OR "white matter"

OR "cortical thickness"

OR "neural correlates"

)

AND

AB,TI(

brain

OR neural

OR neuroimaging

OR neurophysiolog*

OR electrophysiolog*

)

NOT

AB,TI(

animal*

OR rat

OR rats

OR mouse

OR mice

OR rodent*

)
