# Supplementary material for: The Neuroscience of Religious and Spiritual Practices: A Systematic Review of Neurotheological Evidence
Source: Brain Behav. 2026 Aug 30;16(9):e71733. doi: 10.1002/brb3.71733 (PMC13527413; doi:10.1002/brb3.71733)
Supplement: Supplementary file 2 [file BRB3-16-e71733-s002.docx]

*Supplementary Material 2: The Influence of Religious and Spiritual Practices on the Nervous System: A Systematic Review of Neurotheological Evidence*

| **Sr.** | **Reference** | **Year** | **Aim/Objective** | **Sample Size** | **Women**  **%** | **Mean Age** | **SD Age** | **Instruments** | **Religion of Participants** | **Religious Practice** | **Spiritual Practice** | **Brain Region Involved** | **Major Findings** | **Conclusion** |
| --- | --- | --- | --- | --- | --- | --- | --- | --- | --- | --- | --- | --- | --- | --- |
| 1 | (Ab Aziz et al., 2026) | 2026 | To examine brain-wide gamma band (30 to 80 Hz) synchronization and functional connectivity during passive listening to three rhythmic styles of Quranic recitation (Ayatul Kursi verse), comparing Muslim and non-Muslim, Arabic-naive participants using simultaneous MEG/EEG recording. | 29 (14 Muslim, 15 non-Muslim) |  |  |  | MEG; EEG | Islam | Quranic Recitation |  | Supramarginal gyrus, anterior cingulate cortex, hippocampus, central (sensorimotor) region, temporal lobe, inferior frontal gyrus, Rolandic and frontal operculum, cerebellum, thalamus, visual network regions (middle and inferior occipital gyri), superior parietal gyrus | Gamma activity was distributed across a broad language, musical, emotional, memory, and multisensory processing network in both Muslim and non-Muslim listeners, with no meaningful difference in overall brain-wide engagement despite non-Muslims not understanding Arabic. Functional connectivity varied by recitation style, linking regions such as the thalamus and anterior cingulate cortex, hippocampus and postcentral gyrus, and occipital and parietal regions. | Rhythmic Quranic recitation engages a brain-wide gamma network supporting language and musical perception, emotional regulation, memory, and multisensory integration in listeners regardless of religious familiarity or Arabic comprehension, suggesting its psycho-spiritual and well-being benefits are not limited to Muslim practitioners. |
| 2 | (Chandia-Jorquera et al., 2026) | 2026 | To characterize the neurophenomenological signatures of pure awareness (PA) during Transcendental Meditation (TM) by combining Temporal Experience Tracing (TET) with a large-scale screening of theoretically motivated EEG markers, comparing TM practitioners with matched controls performing a counting task. | 66 (33 TM practitioners, 33 matched controls) | 36.4 | 40.3 (TM group); 38.9 (Control group) | 6.2 (TM group); 7.5 (Control group) | EEG | Not Specified |  | Meditation | Whole-scalp EEG (128 electrodes); analyses focused on single-electrode measures (not region-specific) and functional connectivity across delta, theta, alpha, and beta bands rather than a localized region | TM practitioners reported significantly greater intensity and variability of pure awareness than controls, independent of years of practice, supporting "automatic self-transcendence." Multivariate classification revealed a double dissociation: when contrasting TM with controls, temporal entropy (permutation entropy) and aperiodic (1/f) dynamics were the strongest discriminators; when contrasting TM with its own resting baseline, low-frequency linear functional connectivity (WPLI, delta/theta) dominated instead. TM showed no carry-over effects into subsequent rest, unlike controls, whose counting task produced residual EEG changes in the following baseline. | Awareness during Transcendental Meditation is marked by a distinctive, dissociable neural signature: relative to ordinary cognition it is characterized by increased entropy and scale-free (aperiodic) neural dynamics, while relative to resting baseline it is marked by enhanced slow-frequency functional connectivity. This suggests pure awareness is both a more variable/information-rich state than ordinary wakefulness and a more stabilized, transient state relative to rest, supporting neurophenomenology as a robust framework for studying minimal phenomenal experience in contemplative/spiritual practice. |
| 3 | (Kolev et al., 2026) | 2026 | To apply spectral Granger causality analysis to EEG recordings obtained during Focused Attention Meditation (FAM), Open Monitoring Meditation (OMM), and Loving Kindness Meditation (LKM) in highly experienced meditators, in order to uncover distinct directed connectivity signatures, including strength, frequency band, and direction of inter regional information transfer, associated with each meditation style. | 22 | 18.2 | 44.2 |  | EEG | Buddhism | Monastic Buddhist practice; participants were monks, nuns, and novice practitioners residing at Amaravati Buddhist Monastery (Southern England) and Santacittarama Monastery (Central Italy), practicing approximately 100 hours per month during monastic life with an average lifetime practice of 19,358 hours (range 900 to 50,600 hours) | Meditation; Three meditation forms practiced in an integrated, balanced way within the Thai Forest Theravada tradition: Focused Attention Meditation (Samatha, sustained attention on breath sensations), Open Monitoring Meditation (Vipassana, open receptive awareness of arising experience without judgment), and Loving Kindness Meditation (Metta, generating and sustaining kindness and friendliness toward self and all beings) | Left and right anterior (frontal) clusters and left and right posterior (parietal/occipital) clusters, encompassing frontal, fronto-central, centro-parietal, parietal, and occipital electrode sites | Each meditation style produced a distinct pattern of directed connectivity relative to rest. FAM showed significant reduction in posterior to anterior Granger causality (GC) in alpha and beta bands and decreased multi-spectral inter-hemispheric frontal GC, suggesting attenuated bottom-up sensory input and reduced need for inter-hemispheric executive coordination. OMM showed significantly increased multi-spectral (theta, alpha, beta) GC from the left hemisphere to right posterior regions, suggesting enhanced top-down modulation of right parietal regions implicated in expanded awareness and interoceptive processing. LKM showed a distinctive symmetric, bi-directional posterior-anterior cross-hemispheric increase in GC specifically in the beta band (left frontal to right posterior and right frontal to left posterior), suggesting balanced co-activation of approach and withdrawal related emotional systems supporting equanimity and compassionate mental states. | Long-term Buddhist contemplative practice within the Thai Forest Theravada tradition produces highly specific, dissociable patterns of directed neural information flow depending on the meditation style practiced, with Focused Attention Meditation showing reduced sensory and executive information transfer, Open Monitoring Meditation showing enhanced left-to-right posterior information flow linked to expanded awareness, and Loving Kindness Meditation showing a unique symmetric beta-band signature linked to compassion and emotional balance. These findings provide neurotheological evidence that distinct traditional meditative practices, each rooted in a shared religious framework but cultivating different cognitive and affective faculties, engage dissociable large-scale brain network dynamics, though the modest sample size and cross-sectional design limit causal conclusions about the effects of meditation training itself. |
| 4 | (Manea et al., 2026) | 2026 | To examine whether a candidate EEG-derived Transcendence Index (TI), computed as the relative balance of theta-alpha activity versus beta activity, is associated with EEG oscillatory activity across canonical frequency bands, and whether prayer and relaxation show descriptively distinct oscillatory patterns during eyes-closed internal-focus states. | 39 |  |  |  | EEG | Not specified | Prayer |  | Not localized to specific regions (8-channel scalp EEG at Fz, C3, Cz, C4, Pz, PO7, Oz, PO8) | The Transcendence Index (TI), a ratio of theta plus alpha power relative to beta power, was significantly higher during prayer than during relaxation in a within-subject comparison (t(38) = 2.18, p = 0.035, Cohen's d = 0.35). TI correlated positively with theta (r = 0.547, p < 0.001) and low alpha (r = 0.592, p < 0.001), and negatively with high beta (r = -0.592, p < 0.001). TI was also negatively associated with extraversion (r = -0.335, p = 0.037), agreeableness (r = -0.330, p = 0.040), and emotional stability (r = -0.363, p = 0.023). A delta-augmented variant of the index was positively associated with anxiety (r = 0.469, p = 0.003). | Prayer and relaxation produce partially distinguishable EEG spectral profiles, with prayer associated with a greater relative predominance of theta-alpha activity over beta activity than relaxation. |
| 5 | (Osaka et al., 2026) | 2026 | To investigate the neural mechanisms underlying consciousness in Zen meditation by examining central executive network (CEN) and default mode network (DMN) activity and connectivity during a cognitive conflict task (Stroop) followed by meditation, comparing experienced Zen monks with meditation-naive controls. | 37 (19 Zen monks, 18 controls) | 0 | Monks: 39.26; Controls: 38.83 | Monks: 5.91; Controls: 6.35 | fMRI | Buddhism | Meditation | Zen Meditation | Medial Prefrontal Cortex (MPFC) and Anterior Cingulate Cortex (ACC) (Default Mode Network), Dorsolateral Prefrontal Cortex (DLPFC) (Central Executive Network), Precuneus, Posterior Cingulate Cortex (PCC), Posterior Parietal Cortex (PPC), Lateral Parietal (LP) cortex, Anterior Insula, Supramarginal Gyrus | Zen monks showed significantly reduced BOLD activation compared to controls in the MPFC/ACC, DLPFC, and precuneus during the Stroop task, with a significant group by condition interaction in the MPFC/ACC (F(2,70) = 9.10, p = 0.0003) and DLPFC (F(2,70) = 9.25, p = 0.0003). Behaviorally, controls showed significantly slower response times in incongruent versus neutral trials (p < 0.005), while monks showed no such difference, and monks had significantly higher accuracy than controls in incongruent trials (p < 0.05). Contrary to the classic antagonistic CEN-DMN model, Stroop conflict trials did not show a strong inverse relationship between DMN and CEN activity. Functional connectivity analysis during the meditation period revealed significant inhibitory connections among the PCC (DMN), PPC (CEN), and lateral parietal cortex (FDR corrected, p < 0.05), a pattern not observed during the conflict or control conditions. | Long-term Zen meditation training is associated with reduced activation across both DMN and CEN regions during cognitive conflict, consistent with enhanced attentional efficiency and suppressed mind-wandering in experienced monks. Rather than operating as strictly antagonistic systems, the DMN and CEN appeared to share attentional resources during conflict processing, with connectivity analyses indicating that meditation specifically suppresses DMN-related network activity. |
| 6 | (Plácido et al., 2026) | 2026 | To analyze the topological indices of dynamic brain functional networks (BFN), using EEG and motif synchronization, during alleged mediumistic trance compared to a prayer control task, in order to characterize mediumistic trance as a distinct brain state. | 28 (14 Mediums Group, 14 Control Group) | 100 | 49.71 | 11.04 | EEG | Spiritism |  | Psychophonic mediumship trance | Whole scalp (20 electrodes, 10-20 system); left hemisphere electrodes (F7, T3, T5, FP1, F3, C3, P3, O1, FT7) and right hemisphere electrodes (F8, T4, T6, FP2, F4, C4, P4, O2, FT8); left hemisphere showed the most pronounced changes (theta weighted degree and alpha E-index) | During mediumistic trance, the Mediums Group showed significantly increased weighted degree (greater connectivity) in the delta band (whole head, both hemispheres) and theta band (whole head and left hemisphere) compared to the pre-trance baseline. The alpha band E-index showed reduced inter-hemispheric heterophilia during trance relative to pre-trance (whole head and left hemisphere), indicating a shift toward more balanced or intra-hemispheric connectivity. No significant hub changes occurred in the Mediums Group. In contrast, the Control Group (prayer task) showed no significant changes in delta, theta, or alpha, but showed a significant decrease in hub probability in the beta band during prayer relative to baseline, in both hemispheres. | Mediumistic trance is associated with increased and left-lateralized electroencephalographic connectivity in the delta, theta, and alpha bands, distinguishing it topologically from both the resting baseline and from the prayer-based control condition. These findings support the interpretation of mediumistic trance as an altered, non-pathological state of consciousness with a distinct neural signature, separate from sleep-like states and from ordinary devotional prayer, reinforcing the relevance of dynamic brain network approaches for neurotheological research into spiritually framed altered states. |
| 7 | (Chen et al., 2025) | 2025 | To develop a novel loving kindness meditation intervention integrating cognition and behavior (LKM-CB) for major depressive disorder, examine its clinical effect on depression symptoms (Study 1), and explore the underlying neural reward mechanism using multimodal neuroimaging including task based fMRI, resting state ALFF, and structural MRI (Study 2). | Study 1: 61 (30 intervention, 31 control); Study 2: 16 MDD patients (subset who completed neuroimaging) | Study 1: 62.3% (38 of 61: 17 of 30 intervention, 21 of 31 control); Study 2: 50% (8 of 16) | Study 1: 22.50 (intervention group median), 22.00 (control group median); Study 2: 27.63 | Study 2: 9.80 (Study 1 reported medians with interquartile ranges rather than mean/SD due to non-normal distribution) | MRI; fMRI | Not specified |  | Meditation | Middle orbitofrontal cortex (OFC), medial OFC, anterior cingulate (AC), insula, hippocampus/parahippocampal region, middle frontal cortex, superior frontal cortex, inferior occipital gyrus (IOG), middle and superior occipital cortex, middle and inferior temporal cortex | After 8 weeks of LKM-CB, patients showed significantly reduced depression scores (BDI-II, PHQ-9, HAMD) compared to controls (Study 1) and compared to their own pre-intervention scores (Study 2). Neuroimaging revealed a dissociation in reward related brain activation: patients showed lower activation in frontal-striatal regions (middle OFC, AC, insula) for win and neutral outcomes and for anticipation following loss feedback, but higher activation in frontal-striatal regions (medial/middle OFC, hippocampus) during loss outcomes and anticipation following win feedback, indicating hyperactivation during reward anticipation paired with hypoactivation during reward outcome. Resting state ALFF and gray matter volume also increased in frontal cortex. Conversely, non-reward temporal-occipital cortex showed increased task related activation for loss and neutral conditions but decreased resting state ALFF and gray matter volume after the intervention. Behavioral response times on the card guessing task did not change significantly. | A novel cognition and behavior integrated loving kindness meditation (LKM-CB) is effective in reducing clinical depression in patients with major depressive disorder, and its therapeutic effect appears to be mediated by a normalization of reward circuit function, characterized by enhanced anticipatory reward processing alongside dampened reward outcome processing, together with reduced engagement of non-reward temporal-occipital regions. These findings extend the neurotheological evidence base by showing that a contemplative practice rooted in loving kindness and compassion, when combined with cognitive and behavioral elements, can reshape reward related neural circuitry implicated in anhedonia, offering a plausible brain based mechanism for the antidepressant effects of spiritually derived meditative practice, though the small neuroimaging sample, lack of a control group in Study 2, and concurrent antidepressant medication use warrant cautious interpretation. |
| 8 | (Kanwade et al., 2025) | 2025 | To investigate the effects of OM chanting on brainwave patterns using EEG, in order to identify and quantify changes in brain activity before and after chanting, and to discuss possible cognitive and therapeutic implications. | 25 | 52 |  |  | EEG | Hinduism | Chanting |  | Frontal, central, and parietal regions (assessed via electrode pairs F3/F4, C3/C4, and P3/P4), with the strongest alpha power increase in frontal regions and the strongest theta power increase in central regions | Alpha relative power increased by 34.2% (from 24.56% to 32.94%) and alpha coherence improved by 27.8% after chanting. Theta relative power increased by 42.3%, and the theta to beta ratio improved by 85.4%. Beta relative power decreased by 28.5%. Peak to peak amplitude dropped by 32.56%, and amplitude variance decreased by 82.22% (both statistically significant, p < 0.01). The Pearson correlation coefficient between EEG signals strengthened from -0.962 to -0.974, indicating greater inter-hemispheric synchronization. Right hemisphere dominance was observed, with the strongest lateralization in frontal alpha activity. Derived metrics also showed improvements: Global Field Power decreased by 21.7%, Phase Lag Index increased by 25.8%, sample entropy (complexity) increased by 17.7%, and the Synchronization Index increased by 33.3%. | Chanting produces measurable neurophysiological changes consistent with relaxation, reduced cognitive load, and improved emotional regulation, including increased alpha and theta activity, decreased beta activity, and greater inter-hemispheric synchronization. These findings suggest chanting could serve as a neurotherapeutic tool for stress management, attention regulation, and emotional well-being, potentially useful in both clinical and non-clinical settings. |
| 9 | (Kurek et al., 2025) | 2025 | To investigate the bioelectric brain activity associated with Zen meditation in an ecologically valid monastic setting, comparing experienced Zen monks with non-meditating controls, using a portable wearable EEG system to examine theta, alpha, and beta band power during meditation, mind-wandering, and across short versus long meditation sessions. | 15 | 0 | 53 |  | EEG | Buddhism | Meditation | Meditation | Frontal regions (Fp1, Fp2) and temporal regions (T3, T4) | Zen monks exhibited significantly higher alpha and theta band power during meditation compared to inexperienced controls at frontal electrodes (Fp1, Fp2 for theta; Fp1, Fp2, T4 for alpha), with no significant differences in beta power between groups. No significant differences were found between meditation and mind-wandering states within monks, nor between short (4 minute) and long (1.5 hour) meditation sessions, suggesting a high degree of continuity between meditative and non-meditative states in advanced practitioners. Temporal analysis of long meditation sessions showed individual variation, with one monk showing a steady rise in alpha power and another showing peak alpha around 20 to 21 minutes followed by increases in theta and beta later in the session, accompanied by a slight slowing of dominant alpha frequency from 10 Hz to 9 Hz. | Zen meditation is characterized by a distinct neural signature combining elevated frontal alpha and theta activity without corresponding changes in beta power, differentiating it from mindfulness and other meditation traditions where beta reductions are commonly observed. The near absence of difference between meditative and mind-wandering states in advanced monks suggests that sustained, decades-long religious contemplative practice can generate a stable, trait-like neurophysiological state that persists even outside formal meditation, offering neurotheological evidence that deeply rooted spiritual discipline reshapes baseline brain function rather than producing only transient state changes, though the small sample size and use of a consumer-grade, low-density EEG system limit the strength and generalizability of these conclusions. |
| 10 | (Lee et al., 2025) | 2025 | To characterize EEG spectral power (alpha, beta, gamma bands) during Chan/Zen intuitive inquiry meditation (Can-Hua-Tou) in experienced Buddhist monks compared to novice practitioners, testing whether this cognitively demanding, doubt-driven practice produces a distinct oscillatory profile from relaxation-based meditation styles. | 38 (20 monks, 18 novices) | 0 | Monks: 48; Novices: 47 | Monks: 13; Novices: 11 | EEG | Buddhism | Meditation | Zen inquiry meditation (Can-Hua-Tou) | Frontal and bilateral temporal lobes (beta band), fronto-parietal and parieto-occipital regions (gamma band), occipital regions (alpha band, O1, O2, Oz) | Monks showed significantly higher beta power over parietal-occipital regions than novices across all conditions, present even in the non-meditative control task. Monks also showed trait-like elevated gamma power across the whole scalp regardless of condition (rest, control task, or meditation), with novices showing consistently low gamma throughout. Alpha power increased with eyes closed in both groups but showed no significant group or condition differences after correction. | Long-term Can-Hua-Tou practice is associated with stable, trait-like beta and gamma elevations rather than the alpha and theta dominance typical of relaxation-based meditation, suggesting that different contemplative styles produce distinct neurophysiological signatures tied to their specific cognitive demands (sustained concentration and introspective vigilance in this case). |
| 11 | (Lu et al., 2025) | 2025 | To develop and apply a new neuroscientific index, the alpha transformation index, using an EEG neurofeedback procedure to investigate active cortical control and mental flexibility in Buddhist meditation practitioners compared to nonpractitioners, and to examine its relationship with self esteem, subjective well being, and Buddhist mindfulness levels. | 56 (28 Buddhist meditation practitioners, 28 nonpractitioners) | 71.4% in practitioners, 42.9% in nonpractitioners |  |  | EEG | Buddhism | Meditation | Meditation | Prefrontal cortex (F3 and F4 sites, per International 10 to 20 system) | After controlling for age and gender, Buddhist practitioners showed a significantly higher alpha transformation index (reflecting the ability to flexibly transition between alpha enhancement and suppression states) compared to nonpractitioners, though no group differences emerged in alpha enhancement or suppression trials alone. Practitioners also reported significantly higher self esteem, life satisfaction, and lower negative affect. The alpha transformation index was positively correlated with life satisfaction and, within the practitioner group, was significantly correlated with Buddhist mindfulness scores, which were themselves positively associated with self esteem, life satisfaction, and positive affect, and negatively associated with negative affect. | Buddhist meditation practice is associated with enhanced flexible cortical control, as indexed by the novel alpha transformation measure, supporting the Unified Flexibility and Mindfulness model's proposal that mental flexibility is a key mechanism through which mindfulness produces its psychological benefits. These findings suggest that sustained Buddhist spiritual practice cultivates the neural capacity to voluntarily shift between relaxed and focused brain states, offering a plausible neurophysiological pathway linking traditional contemplative practice to improved well being, though the modest sample size, unmatched demographic characteristics between groups, and lack of differentiation among meditation subtypes warrant cautious interpretation. |
| 12 | (Pascarella et al., 2025) | 2025 | To characterize changes in brain oscillations, signal complexity, and criticality related dynamics in expert Buddhist monks during Samatha (focused attention) and Vipassana (open monitoring) meditation compared to resting state, using source space MEG combined with machine learning. | 12 | 0 | 38.7 | 10.9 | MEG | Buddhism | Meditation | Samatha (focused attention) and Vipassana (open monitoring) | Frontal, anterior cingulate, lingual, and insular regions (Samatha discrimination from rest); superior frontal, precentral, and parietal regions (Vipassana discrimination from rest); frontal, parietal, occipital, and temporal regions (aperiodic slope changes); Ventral Attention, Dorsal Attention, Motor, and Default Mode Networks (criticality changes) | Both meditation styles showed a flattened aperiodic (1/f) slope, reduced long range temporal correlations in gamma bands and broadband signal, and increased complexity (Lempel-Ziv complexity, Higuchi fractal dimension, spectral entropy) compared to rest. After correcting for the aperiodic component, both practices showed reduced gamma power, contrasting with earlier reports of gamma increases during meditation. A machine learning classifier distinguished meditation from rest with high accuracy but could not reliably distinguish Samatha from Vipassana, except for a criticality measure (deviation from criticality coefficient) that showed a trend level separation between the two styles. | Samatha and Vipassana meditation both shift brain dynamics toward greater neural complexity and reduced temporal persistence relative to rest, but show subtle differences in criticality related measures, suggesting that focused attention and open monitoring meditation, despite shared surface level EEG/MEG signatures, may be supported by distinct underlying computational dynamics. |
| 13 | (Reggente et al., 2025) | 2025 | To determine the feasibility of decoding self-reported, time-varying meditative depth from EEG activity in expert Vipassana practitioners during prolonged periods of routine practice, using both traditional probing and a novel "spontaneous emergence" self-reporting method, and to identify neural correlates (spectral power and effective connectivity in theta, alpha, and gamma bands) associated with subjective depth gradations. | 34 | 76 | 46.53 | 13.09 | EEG | Not specified | Meditation | Vipassana Meditation | Default Mode Network (bilateral angular gyrus, medial prefrontal cortex, posterior cingulate cortex); frontal-midline, parietal, and occipito-parietal regions | No single EEG frequency or connectivity measure alone distinguished meditative depth, but multivariate machine learning decoded self-reported depth from theta, alpha, and gamma activity with above-chance accuracy. Lower depth states showed relatively higher beta and gamma power in centro-parietal regions compared to deeper states. | Meditative depth in expert Vipassana practitioners is a graded, multidimensional neural state best captured by combined spectral and connectivity patterns rather than any single EEG marker, supporting EEG as a viable tool for objectively indexing contemplative depth. |
| 14 | (Sharma et al., 2025) | 2025 | To investigate the impact of long-term Gayatri Mantra meditation on emotional processing using event-related potentials (specifically the Late Positive Potential) during passive viewing of emotionally arousing images, comparing meditation practitioners with non-practitioners. | 24 (12 practitioners, 12 non-practitioners) | 0 | 32.37 | 8.02 | EEG; ERP | Hinduism |  | Gayatri Mantra meditation | Mid-centroparietal region (CPz), right centroparietal region (CP2, CP4, CP6), left centroparietal region (CP1, CP3, CP5) | practitioners showed significantly reduced Late Positive Potential amplitudes compared to non-practitioners, most notably for negative stimuli at the mid-centroparietal site, with a similar trend at right centroparietal sites. Practitioners also scored significantly higher on emotional competence and non-attachment (anasakti), and showed a consistent pattern of negative correlations between LPP amplitude and emotional traits, unlike non-practitioners whose correlations were weak and inconsistent. | Practitioners showed significantly reduced Late Positive Potential amplitudes compared to non-practitioners, most notably for negative stimuli at the mid-centroparietal site, with a similar trend at right centroparietal sites. Practitioners also scored significantly higher on emotional competence and non-attachment (anasakti), and showed a consistent pattern of negative correlations between LPP amplitude and emotional traits, unlike non-practitioners whose correlations were weak and inconsistent. |
| 15 | (Wenuganen et al., 2025) | 2025 | To examine possible anti-aging and anti-stress effects of long-term Transcendental Meditation (TM) practice by comparing gene expression (via qPCR), EEG correlates of cognitive function (ERP latencies and the Brain Integration Scale), and hair glucocorticoid concentrations between TM practitioners and non-practitioner controls across two age groups. | Total sample varied by analysis. Gene expression: 63 (19 Young Control, 21 Old Control, 23 Old TM). Cognitive/EEG performance: 103 (25 Young Control, 30 Young TM, 22 Old Control, 26 Old TM). Hair glucocorticoid analysis: 108 (29 Young Control, 26 Young TM, 23 Old Control, 30 Old TM). | Varied by subgroup. For the EEG cognitive sample (N=100 with recorded sex): approximately 48% women (52 males of 100). Other subgroups had similar approximate gender distribution (roughly 50 to 60% male across groups). | Young groups: approximately 24.0 to 24.4 years. Old groups: approximately 62.4 to 64.8 years. | Young groups: approximately 2.6 to 3.1. Old groups: approximately 3.5 to 4.7. | qPCR; EEG; LC/MS/MS | Not specified |  | Transcendental Meditation | Frontal-central regions (N2 and P3a components); parietal region (P3b component); broadband frontal regions (EEG coherence measure within the Brain Integration Scale); whole blood immune cells were used for gene expression rather than a specific brain region. | Long-term TM practitioners showed significantly shorter N2, P3a, and P3b latencies compared to age-matched controls, with the Old TM group showing latencies not significantly different from the Young Control group for N2 and P3b, suggesting a more youthful pattern of neural processing speed. TM practitioners also had significantly higher Brain Integration Scale scores than controls, with Old TM scores comparable to Young Control scores. At the molecular level, several aging-associated genes (including SOCS3, ITGB3, ITGB5, LMNA, TAL1, and ALOX12) were downregulated in the Old TM group relative to Old Controls, moving expression levels closer to those seen in the Young Control group. TM practitioners also showed lower hair cortisol and lower cortisol to cortisone ratios than controls, consistent with reduced long-term HPA axis activation. | Long-term practice of Transcendental Meditation is associated with converging neurophysiological, molecular, and endocrine markers suggestive of reduced allostatic load and a more youthful pattern of brain function with aging. |
| 16 | (Krause-Sorio et al., 2024) | 2024 | To conduct a pilot fMRI study exploring the neural correlates of watching digital art or nature videos compared to contemplating universal connectedness (a form of transcendental meditation) in healthy adults with sub-clinical levels of stress, anxiety, and depression. | 9 | 55.6 | 29 |  | fMRI | Not specified |  | Transcendental Meditation | Bilateral lateral occipital cortices, bilateral occipital fusiform gyri, right postcentral gyrus, and right hippocampus (meditation compared to rest); left parietal and central operculum and right pre- and postcentral gyri (meditation compared to art); left parietal operculum, bilateral postcentral and supramarginal gyri, and bilateral lateral occipital cortices (meditation compared to nature). | Compared to rest, the brief guided meditation task increased BOLD activity in regions associated with visual object processing (lateral occipital and fusiform gyri), sensory processing (postcentral gyrus), and memory (hippocampus). Compared to viewing AI-generated digital art, meditation showed greater activation in the parietal and central operculum and pre- and postcentral gyri, regions linked to sensory integration, interoception, and possibly self-monitoring. Compared to nature videos, meditation showed greater activation in sensory, object-processing, and sensory-integration regions, including the supramarginal gyrus, an area also linked to emotion regulation. No brain regions showed greater activation for art or nature compared to meditation. There was no significant change in state anxiety before versus after the scan session, and correlations between anxiety scores and brain activity did not survive correction for multiple comparisons. | In this small pilot study, a brief, novice-guided form of transcendental meditation focused on universal connectedness engaged a distinct pattern of brain activity compared to passive viewing of nature or art videos, particularly in regions tied to sensory integration, interoception, and object processing, suggesting that even short-term contemplative practice recruits neural systems beyond those activated by simple visual stimulation alone. These preliminary findings tentatively support the idea that meditative practices aimed at inducing a sense of transcendence or universal connection engage a partly distinct neural signature relative to other calming visual experiences, though the very small sample size, lack of correction for multiple task comparisons, and single-block presentation of the meditation condition limit how strongly these conclusions can be drawn. |
| 17 | (Mallaroni et al., 2024) | 2024 | To characterise changes in functional connectome identifiability under the altered state of consciousness induced by ritualistic ayahuasca consumption, examining static and dynamic connectivity, the preservation of individual neural identity, and the relationship between connectivity patterns and subjective experience. | 21 | 48 | 54.48 | 10.55 | fMRI | Santo Daime | Ayahuasca Ritual | Ritual Song, Dance, And Contemplative Silence | Default Mode Network (DMN), Visual Network (VIS), Ventral Attention Network (VA), Dorsal Attention Network (DA), Somatomotor Network (SM), Fronto-parietal Network (FPN), and Limbic Network (L) | Ayahuasca ingestion reduced connectome idiosyncrasy; individual functional connectivity fingerprints became less distinct. Self-identity was nonetheless preserved through alterations in the network basis of identification, with the default mode network assuming a more central role. Brain timing hierarchy patterns were inverted. Attention network connectivity stability changed significantly. Static functional connectivity patterns in visual and auditory networks predicted intensity of visual restructuralisation and auditory alteration experiences respectively. | Ritualistic ayahuasca consumption induces a systematic reorganization of functional connectome architecture, reducing individual idiosyncrasy while preserving identity through altered network substrates. The default mode network's assumption of an identity-anchoring role during psychedelic states may explain the paradoxical co-occurrence of ego dissolution with preservation of biographical self-awareness. |
| 18 | (Mohanty et al., 2024) | 2024 | To investigate the impact of Mahā Mantra (MM) chanting on anxiety and depression through quantitative EEG rhythm analysis, examining oscillatory changes in central and parietal regions following the intervention. | 40 | 50 | 25.5 |  | EEG | Hinduism | Chanting | Mantra Meditation | Central region C3-C4 and Parietal region P3-P4 | Mahā Mantra chanting produced a significant increase in alpha relative power in the central (C3, C4) and parietal (P3, P4) regions. Increased theta and delta wave production were also observed. These electrophysiological changes are associated with neurotransmitter release patterns inducing vasodilation and indicative of enhanced mental relaxation and cognitive refreshment. | Mahā Mantra chanting is associated with significant central and parietal alpha augmentation, reflecting a state of relaxed, alert awareness. Concurrent theta and delta enhancement suggests engagement of deeper restorative neural states. Autonomic nervous system modulation through neuroendocrine pathways may mediate reported improvements in mental wellbeing. |
| 19 | (Rosmarin et al., 2024) | 2024 | To conduct an exploratory investigation of neurobiological correlates of positive and negative religious coping among older adults with and without mood disorders, using resting state fMRI to examine within network and between network connectivity of three networks in Menon's Triple Network Model (cingulo-opercular salience network, central executive network, and default mode network). | 32 (15 with unipolar/bipolar mood disorders, 17 healthy controls) | 50 | 62.56 | 4.99 | MRI | Mixed | Religious service attendance and private religious practice | Religious coping strategies, both positive (e.g., prayer, asking forgiveness) and negative (e.g., questioning God's love), as measured by the Brief RCOPE; general spirituality and religiosity importance also assessed | Left insula, cingulo-opercular salience network (cSN), default mode network (DMN), central executive network (CEN) | Greater negative religious coping was associated with increased within network connectivity in the cingulo-opercular salience network, specifically involving the left insula, an effect that remained significant after controlling for depression severity. Both negative and positive religious coping were associated with greater anti-correlation (reduced connectivity) between the DMN and CEN, reflecting cognitive effort associated with coping regardless of valence, and these associations also survived after controlling for depression. No significant associations were found between religious coping and salience network to CEN or DMN to salience network connectivity, and positive coping was not related to within network connectivity in any of the three networks. | Religious coping, particularly negative religious coping, is associated with distinct neurobiological signatures within brain networks implicated in psychopathology, including heightened insula related salience network connectivity and altered DMN-CEN coupling reflecting cognitive effort. These preliminary findings extend the neurotheological evidence base by demonstrating that religious and spiritual coping processes engage large scale brain networks relevant to emotional processing and self-regulation in older adults, though findings do not yet inform clinical intervention and require replication in larger samples. |
| 20 | (Saini et al., 2024) | 2024 | To compare the effects of verbal "OM" chanting (VOM) and listening to "OM" chanting (LOM) on cortical sources, as compared to baseline, using high-density quantitative electroencephalography (qEEG) and standardized low-resolution electromagnetic tomography (sLORETA) source localization. | 20 | 0 | 27.5 | 7.5 | EEG; sLORETA | Not Specified | Chanting |  | Widespread cortical activation across 66 gyri, including the anterior cingulate, angular gyrus, cingulate gyrus, medial and middle frontal gyrus, cuneus, fusiform gyrus, inferior frontal gyrus, inferior parietal lobule, insula, middle and superior temporal gyrus, precentral and postcentral gyrus, precuneus, parahippocampal gyrus, superior frontal and parietal lobule, and supramarginal gyrus; with the rectal gyrus, orbital gyrus, and sub callosal gyrus | Both verbal and listening OM chanting produced significant increases in cortical source activity compared to their respective pre-chanting baselines (29 gyri activated post-VOM, 33 gyri activated post-LOM), with no significant difference found between post-VOM and post-LOM conditions directly. The activated regions in both conditions could be classified as nodes of several resting-state networks, including the frontoparietal control network (FPCN), dorsal attentional network (DAN), ventral attentional network (VAN), and default mode network (DMN). Listening to OM chanting uniquely activated additional orbitofrontal and limbic areas (rectal gyrus, orbital gyrus, sub callosal gyrus) not seen in verbal chanting, contrasting with a separate study that had reported limbic deactivation during OM chanting. | Both verbal and listening forms of OM chanting activate overlapping cortical regions corresponding to major resting-state networks associated with attention, salience, and mind-wandering, suggesting that OM chanting may facilitate flexible switching between the frontoparietal control network and the default mode and attentional networks. |
| 21 | (Sik et al., 2024) | 2024 | To investigate the neural correlates of repetitive religious chanting using a multi-modal neuroimaging approach (EEG combined with fMRI and peripheral physiological measures), with focus on self-related brain regions and the associated emotional and autonomic changes during religious versus non-religious chanting. | 21 |  |  |  | fMRI; EEG; ECG; HRV; GSR | Buddhism | Chanting | Devotional Chanting Meditation | Posterior Cingulate Cortex | fMRI eigenvector centrality analysis revealed the strongest decrease in posterior cingulate cortex (PCC) during religious chanting compared to non-religious chanting. EEG analyses demonstrated significantly higher delta-band power during religious chanting. Heart rate variability total power, absolute high-frequency power, and absolute very-low-frequency power were all significantly reduced during religious chanting relative to resting state. | Religious chanting significantly reduces PCC network centrality; a core default mode network hub; consistent with diminished self-referential processing and the subjective experience of transcendental absorption. Concurrent delta augmentation and parasympathetic modulation indicate a deeply relaxed yet inwardly attentive state that is neurobiologically distinct from non-religious chanting. |
| 22 | (Sumarti et al., 2024) | 2024 | To use EEG to determine whether Istighfar Dhikr (Islamic recitation of seeking forgiveness) modulates brain wave activity during states of induced anxiety, and to examine whether these effects differ between males and females. | 24 | 50 |  |  | EEG | Islam | Dhikr | Istighfar Dhikr | Frontal cortex, using frontal electrode pairs referenced to the ears (Fp1 to A1, Fp2 to A2, F3 to A1, F4 to A2, F7 to A1, F8 to A2), focusing on alpha and beta frequency bands | Both alpha and beta brain wave amplitudes increased from the anxiety condition to the istighfar condition in nearly all participants, with only one female subject showing a beta wave increase pattern that deviated from the group trend. Logistic binary regression revealed a gender-based divergence: in males, the change in alpha wave activity was statistically significant (p = 0.016) while the change in beta wave activity was not (p = 0.188); in females, the reverse pattern held, with beta wave changes being statistically significant (p = 0.032) while alpha wave changes were not (p = 0.996). | Istighfar Dhikr, even in a brief three minute silent recitation format, produced measurable, objective changes in frontal EEG activity following induced anxiety, providing EEG based evidence in support of the traditionally held belief that this Islamic devotional practice can help alleviate anxiety and promote emotional stability. The findings additionally suggest that the specific neural marker of this calming effect differs by gender, with alpha wave dominance characterizing the male response and beta wave dominance characterizing the female response, indicating that the neurophysiological signature of religious or spiritual practice may not be uniform across sexes. |
| 23 | (Walter, 2024) | 2024 | To analyze neurophysiological correlates of religious experiences during worship with music, specifically comparing religious versus secular songs and self-selected versus researcher-given songs, using EEG spectral analysis. | 57 | 55 | 27 | 4.2 | EEG | Christianity |  | Musical worship | Occipital regions (primary novel finding), also referenced temporal cortex, frontal regions (contrasted against prior literature); topographic activity noted at electrodes such as CP2, T7, PO3, TP7, PO8, FC3, TP9, C1, C2 | Religious songs produced greater neurophysiological activation than secular songs, mainly through increased upper beta waves. This effect was stronger when songs were self-selected, shown by decreased lower alpha activity in the religious self-selected condition. Higher self-reported religious experience correlated with more theta and alpha activity (similar to meditative states), especially in occipital regions. The study did not support the temporal involvement or executive inhibition hypotheses; instead, findings pointed toward a possible new "occipital involvement hypothesis." | Religious worship experiences share neurophysiological features with meditative states (theta and alpha activity), but also involve heightened active thinking and focus (beta activity) tied specifically to the religious content of the experience. The study distinguishes between the "religious experience" (subjective sensing of divine presence) and the "religious element" (content of the stimulus), proposing that both self-selection and religious content independently intensify neural engagement. The unexpected occipital involvement suggests a new avenue for future research beyond the traditional temporal lobe framework. |
| 24 | (AlMahrouqi & Mostafa, 2023) | 2023 | To identify cortical regions selectively responsive to Quranic recitation in Muslim participants, and to determine whether differential neural activation patterns are observed between Muslim and non-Muslim participants during Quran listening. | 18 | 33 | 26.07 | 4.13 | fMRI | Islam | Quranic Recitation |  | Anterior Cingulate Cortex (ACC), Medial Frontal Cortex (MFC), Insula, Superior Temporal Gyrus, Superior Frontal Gyrus, Inferior Parietal Lobe, Precentral Gyrus, Postcentral Gyrus, | Muslim participants demonstrated significantly greater activation in the anterior cingulate cortex, medial frontal cortex, insula, inferior parietal lobule, superior frontal cortex, and superior temporal cortex during Quran recitation relative to non-Muslims. Native Arabic-speaking Muslims additionally exhibited bilateral superior frontal gyrus (BA 9), right medial frontal cortex (BA 10), left insula (BA 13), and right anterior cingulate (BA 32) activation for Quranic stimuli. Non-Muslim participants showed no significant activation in response to Quranic content. | Quranic recitation activates an interconnected network of empathic, mentalizing, and episodic memory regions in Muslim participants, consistent with the "social brain" framework. Engagement of the anterior cingulate and insula is consistent with religious conviction and affective resonance during spiritual exposure. |
| 25 | (Baykara et al., 2023) | 2023 | To evaluate corpus callosum morphology in habitual Islamic prayer practitioners using statistical shape analysis, and to compare callosal shape parameters between prayer practitioners and non-praying healthy controls. | 27 | 41 | 33.74 | 6.89 | MRI | Islam | Prayer |  | corpus callosum | No statistically significant difference was identified in total corpus callosum cross-sectional area between groups. However, statistical shape analysis revealed significant morphological differences (T² = 1447.596, p < 0.001), with maximum deformation in the body (superior-anterior edge), inferior splenium, and rostrum; regions connecting tactile, motor, auditory, and cognitive processing areas. Age-related centroid size trajectories diverged between groups, decreasing in practitioners and increasing in controls. | Regular Islamic prayer practice is associated with region-specific alterations in corpus callosum morphology, particularly in sub-regions connecting cortical areas engaged during prayer (motor, somatosensory, auditory, cognitive). These structural differences may reflect use-dependent white matter remodeling from repeated, coordinated bi-hemispheric activity. |
| 26 | (Perez-Diaz et al., 2023) | 2023 | To determine the neural activity associated with prayer practices within Sahaja Yoga Meditation (SYM), using formalized (Lord's Prayer) and improvised (prayer to the Divine Mother) forms of prayer compared to equivalent secular speech tasks, in order to explore effects beyond simple repetitive speech (the "mantra effect") and test whether SYM prayer recruits social cognition brain regions similar to those seen in Christian prayer. | 16 | 62.5 | 48.2 | 10.3 | fMRI | Mixed (Sahaja Yoga Meditation practitioners, a practice that draws on multiple religious traditions (Christianity, Judaism, Islam, and Hinduism) rather than belonging to a single organized religion; all participants reported believing in God) | Recitation of prayers drawn from multiple traditions, including the Lord's Prayer (Christianity), used within SYM as preparatory practices for meditation | Sahaja Yoga Meditation | Bilateral thalamus and left dorsolateral prefrontal cortex (dlPFC), left ventrolateral prefrontal cortex (vlPFC), and left supplementary motor area (SMA) or dorsal anterior cingulate cortex (dACC) | Both formalized and improvised prayer showed reduced activation in the bilateral thalamus and in frontal regions (left dlPFC, left vlPFC, left SMA and dACC) compared to their respective secular speech equivalents, with no brain regions showing greater activation during prayer than during secular speech in either direction. The medial prefrontal cortex, a region associated with social cognition and theory of mind, showed reduced activation during both prayer conditions and during formalized secular speech, but increased activation during improvised secular speech, suggesting a specific socioemotional regulation effect tied to religious content and repetition rather than general improvisation. | In contrast to findings in Christian practitioners, praying within Sahaja Yoga Meditation was associated with widespread deactivation of the thalamus and frontal regions rather than increased activation of social cognition networks, suggesting that SYM prayer may be neurophenomenologically distinct from prayer forms that rely more heavily on active social cognitive processes such as theory of mind and mentalizing about a divine other. |
| 27 | (Saraei et al., 2023) | 2023 | To compare patterns of brain activation during three Islamic spiritual states;Dhikr (repetitive supplicatory recitation), body-scan meditation, and contemplative thought about God (TAG);relative to resting state, and to determine whether observed neural activation patterns reflect the cultural and religious belief system or the specific ritual form. | 31 | 52 | 30.56 | 6.78 | fMRI | Islam | Dhikr | Body-Scan Meditation | Frontal pole & Anterior cingulate cortex, Caudate, Temporal pole, Supramarginal gyrus, Precuneus, Intracalcarine cortex & Cerebellum, Default Mode Network (DMN), Inferior lateral occipital cortex | All three spiritual conditions produced highly similar neural activation patterns that were primarily differentiated from resting state rather than from each other. Shared activations included the intracalcarine cortex, cerebellum, caudate, anterior cingulate cortex, temporal pole, and supramarginal gyrus. Meditation produced higher precuneus and supramarginal gyrus activation than Dhikr (attributable to body-focused attention), whereas TAG elicited greater inferior lateral occipital activation suggestive of visualization. Contrary to directed meditation paradigms, all three conditions were associated with increased default mode network activity relative to rest. | Islamic spiritual practices; including ritual recitation, contemplative meditation, and theological reflection; engage overlapping attentional, reward, and self-referential networks. Increased caudate and anterior cingulate activation suggests affective engagement and attentional regulation, while DMN upregulation indicates internally-directed, self-referential spiritual processing. |
| 28 | (Chung et al., 2022) | 2022 | To identify patterns of regional brain volume changes in Catholic nuns relative to age-matched lay controls, using deep learning-based automated MRI segmentation. | 193 | 100 | 57.61 | 10.42 | MRI | Christianity | Prayer And Contemplative Practice | Meditation, Spiritual Discipline, And Prosocial Engagement | Lingual cortex, Isthmus-cingulate, Posterior-cingulate, Rostral-middle-frontal cortex, Superior-frontal cortex, Supramarginal cortex, Temporal-pole cortex, Pars-triangularis cortex, Entorhinal cortex, Inferior-temporal cortex, Middle-temporal cortex, Transverse-temporal cortex, Bankssts cortex, Frontal-pole cortex, Pars-orbitalis cortex, Precentral cortex, Superior-parietal cortex, Cerebellum-white-matter, Corpus callosum, Thalamus, Accumbens, Caudate, Putamen, Pallidum, Ventraldc | Nuns demonstrated increased grey matter volume in the right lingual cortex, left isthmus-cingulate, posterior cingulate, rostral middle frontal, superior frontal, supramarginal, temporal pole cortices, and bilateral pars triangularis. Conversely, reduced grey matter volume was observed in temporal and parietal regions, including left entorhinal, inferior temporal, middle temporal, and right superior parietal cortices. Volumetric increases in frontal and cingulate regions are consistent with lifelong engagement in structured religious and contemplative practices. | Sustained religious life characterized by prayer, meditation, prosocial engagement, and spiritual commitment is associated with increased cortical thickness in prefrontal and cingulate regions governing higher-order cognition, self-monitoring, and executive function, alongside structural changes in temporal and parietal areas. These neuroanatomical profiles are associated with lower dementia risk and reduced rates of depression. |
| 29 | (Ismail et al., 2022) | 2022 | To investigate the neural mechanism underlying the psychoacoustic and cognitive enhancement effects of listening to Quranic recitation, specifically examining occipital lobe beta rhythms during listening to the Fatihah Chapter compared to Arabic news and rest. | 28 | 50 |  |  | EEG | Islam | Listening to Quranic recitation |  | Left occipital lobe (electrode O1) | The left occipital electrode (O1) showed a significant reduction in beta band (14-30 Hz) power spectrum while listening to the Fatihah Chapter compared to rest (p = 0.019). No significant change was found in the occipital lobe during listening to Arabic news compared to rest (p = 0.741). Discriminant Analysis supported these findings, showing the Fatihah Chapter condition was well discriminated from rest and Arabic news, with an area under the ROC curve of 0.778 for the Fatihah Chapter (versus 0.737 for Arabic news), indicating higher sensitivity and specificity for the Fatihah Chapter effect. | Listening to the Fatihah Chapter activates an oscillatory neural network in the left occipital lobe associated with visual mental imagery, likely due to the rhythmic qualities of Quranic recitation (in contrast to the less rhythmic Arabic news). |
| 30 | (Kim et al., 2022) | 2022 | To characterise brainwave changes during Juingong meditation using EEG, specifically examining whether the theta-to-alpha ratio in the Alpha-Theta protocol is modulated during this practice relative to instructed mind wandering. | 23 | 78 | 55.3 | 14.01 | EEG | Buddhism | Meditation | Juingong Meditation | Temporoparietal lobes (TP9 and TP10) Anterior frontal regions (AF7 and AF8) were also measured but showed no significant differences. | During Juingong meditation, the theta-to-alpha ratio was significantly lower in temporoparietal channels (TP9, TP10) compared to instructed mind wandering (p < 0.05), reflecting relatively greater alpha power during meditation. This differential was evident from the onset of measurement. No significant differences were found in anterior frontal channels (AF7, AF8). The theta-to-alpha ratio in temporoparietal electrodes correlated with frequency of participation in intensive Juingong training sessions. | Juingong meditation produces an immediate and sustained shift toward alpha dominance in temporoparietal regions, reflecting internal attentional orientation and contemplative absorption. The absence of frontal effects differentiates this practice from focused attention paradigms, suggesting a primarily receptive, rather than executive, attentional mode. |
| 31 | (Majeed et al., 2022) | 2022 | To explore the psychophysiological modulation of diabetic distress in response to Surah Al-Rehman Quranic recitation, examining neurocardiological and electrophysiological parameters including EEG, ECG, heart rate variability, and heartbeat-evoked potentials. | 10 | 40 | 50.7 | 2.2 | EEG; ECG; HRV | Islam | Quranic Recitation | Spiritual Recitation Therapy | Prefrontal Cortex (PEC) Fp1, Fp2, F3, F4 | Surah Al-Rehman recitation produced significant reductions in systolic blood pressure, increases in low-frequency HRV power, and decreases in stress index. Significant modulation of heartbeat-evoked potentials was observed at Fp1, Fp2, and F4 electrode sites. Absolute alpha power increased significantly at Fp2 (right prefrontal cortex). Overall power in theta, alpha, beta, and gamma bands increased in the right prefrontal cortex. | Quranic recitation modulates prefrontal neural activity and heart-brain coherence, as evidenced by increased right prefrontal alpha power and altered heartbeat-evoked potential morphology. These findings indicate enhanced internal attentional focus and improved autonomic-cortical integration, with potential therapeutic implications for stress-related complications in medical conditions. |
| 32 | (Samhani et al., 2022) | 2022 | To investigate the synchronous effects of listening to the Quranic Fatihah Chapter on beta rhythm oscillations and cognition, using EEG combined with multivariate analysis (Repeated Measures ANOVA and Agglomerative Hierarchical Clustering), compared to listening to Arabic News and Rest. | 28 | 50 | 31.12 |  | EEG | Islam | Listening to Quranic recitation (Fatihah Chapter, the first chapter of the Quran, recited with tajweed) |  | Widespread fronto-temporo-parieto-occipital network; bilateral inferior frontal cortex, bilateral motor/central regions (C3, C4), right middle temporal region (T8), right parietal region (P4, P8), left occipital region (O1), frontal midline (Fz, Fp1, Fp2, F3, F4) | Listening to the Fatihah Chapter significantly reduced (desynchronized) beta spectral power across 13 electrode channels spanning frontal, temporal, parietal, and occipital lobes, more extensively than listening to Arabic News (10 channels, frontal and parietal only), indicating that Quranic recitation activated a broader neural ensemble. Clustering analysis identified a highly activated cluster (bilateral inferior frontal, motor areas, right middle temporal, right parietal, left occipital) linked to verbal fluency, inhibitory control, movement planning, self-motivation, and memory trace reactivation; a medially activated cluster (frontal regions) linked to working memory, language processing, and decision making; and a low activated cluster linked to tune recognition and visual mental imagery. | Listening to the Fatihah Chapter engages a broad, rhythm-entrained beta oscillatory network across frontal, temporal, parietal, and occipital regions, supporting cognitive functions including verbal fluency, memory retrieval, inhibitory control, movement planning, and visual mental imagery. These findings suggest that engagement with sacred Quranic recitation produces measurable, spatially distributed neural synchronization effects beyond those elicited by comparable non-sacred Arabic speech, supporting the premise that religious acoustic ritual can shape distinct patterns of brain oscillatory activity relevant to neurotheological inquiry. |
| 33 | (Walter & Koenig, 2022) | 2022 | To investigate large-scale brain network activation patterns during religious worship experiences using EEG microstate analysis, specifically examining the contributions of multisensory integration, default mode, visual, auditory, and salience networks to the subjective experience of divine presence. | 57 | 55 | 27 | 4.2 | EEG | Christianity | Worship | Musical Worship And Sensing Divine Presence | MS1: Left temporal lobe, primary auditory cortex, Wernicke's area, left insula MS2: Left & right occipital cortices MS3: Precuneus, Posterior Cingulate Cortex (PCC), left angular gyrus MS4: Right inferior parietal lobe, right frontal gyri MS5: Dorsal anterior cingulate cortex (ACC), bilateral middle frontal gyrus, bilateral insula | The salience network (microstate 5: dorsal anterior cingulate cortex, bilateral middle frontal gyrus, bilateral insula) showed the strongest negative association with religious experience; the most robust predictor. The default mode network (microstate 3: precuneus, posterior cingulate cortex, left angular gyrus) also showed a significant negative association. The auditory network (microstate 1: left temporal lobe, primary auditory cortex, Wernicke's area, left insula) demonstrated a positive association with religious experience. The multisensory integration and visual networks were not significantly associated. | Religious experience during worship is characterized by downregulation of salience and default mode networks alongside upregulation of the auditory network. Suppression of the salience network; which monitors self-relevant environmental stimuli; during intense worship may underlie the phenomenological quality of self-transcendence and absorption characteristic of religious states. |
| 34 | (Miller et al., 2021) | 2021 | To investigate whether specific spiritual phenotypes;altruism, love of neighbour, interconnectedness, contemplative practice, and commitment to religion/spirituality;are associated with cortical thickness in the Ventral Frontotemporal Network (VFTN), and whether this structural metric confers neuroprotection against major depressive disorder in individuals at high familial risk. | 72 | 61 | 41.43 | 12.67 | MRI | Mixed / Multiple Religions |  | Altruism, Contemplative Practice, And Spiritual Commitment | Ventral Frontotemporal Network (VFTN) | Altruism and Love of Neighbor as Self were significantly associated with greater cortical thickness across the VFTN. Greater VFTN cortical thickness was selectively protective against depression in high-familial-risk participants. Contemplative practice and commitment to religion/spirituality alone did not produce significant VFTN associations. | Altruistic and prosocially-oriented spiritual phenotypes are associated with increased cortical thickness in the ventral frontotemporal network, a region implicated in affective regulation, social cognition, and reward processing. This structural augmentation appears to confer neurobiological resilience against the emergence of depressive disorder in genetically vulnerable individuals. |
| 35 | (Razak et al., 2021) | 2021 | To investigate the response of EEG signals during brain relaxation meditation using different types of zikr (Islamic remembrance of God), and to develop a framework using EEG signals to compare the effects of different zikr types. | 8 | 50 |  |  | EEG | Islam | Dhikr |  | Frontal lobe | Delta wave amplitude was consistently the highest among all recorded brainwave types (Alpha, Beta, Delta) for both Dhikr conditions, across all eight participants, regardless of gender. This pattern held for both Dhikr types tested. A p-value of 0.498 for delta wave activity between the two Dhikr conditions showed no significant difference, indicating both types of Dhikr produced a similarly calming effect. | Reciting or listening to Dhikr, regardless of the specific phrase used, increases Delta wave activity, which is associated with deep relaxation and calmness. This suggests Dhikr practice has potential as a tool to help reduce stress and anxiety, though the authors note further research is needed before practical application. |
| 36 | (Wintering et al., 2021) | 2021 | To determine whether a seven-day Ignatian spiritual retreat produces measurable changes in brain functional connectivity, as assessed by resting-state fMRI, and whether such changes are associated with psychological or spiritual improvements. | 14 | 43 | 54 | 13 | fMRI | Christianity | Spiritual Retreat | Ignatian Spiritual Exercises | Posterior cingulate cortex, Superior frontal gyrus, Pallidum (basal ganglia/dopamine area), Cerebellum, Hippocampus/parahippocampus (limbic system), Superior parietal lobe, Superior and inferior temporal gyri | The retreat produced increased functional connectivity between the posterior cingulate cortex and right superior frontal gyrus, and between the posterior cingulate and left pallidum. Decreased connectivity was observed between the cerebellum and hippocampus/parahippocampus, vermis and cuneus, and superior parietal and temporal areas. Participants showed significant reductions in tension and fatigue alongside increases in religiousness, spirituality, and self-transcendence. | An intensive Ignatian spiritual retreat produces specific resting-state connectivity changes in networks subserving self-awareness (posterior cingulate cortex-frontal connectivity) and dopaminergic-limbic function (pallidum connectivity). Concurrent reductions in cerebellar-hippocampal and parieto-temporal connectivity suggest reorganized emotional-spatial self-processing following sustained contemplative engagement. |
| 37 | (Young et al., 2021) | 2021 | To identify electrophysiological correlates of meditation practice by comparing EEG oscillatory activity (power spectra) and entropy (Lempel-Ziv complexity) across six different meditation styles (shamatha, vipassana, zazen, dzogchen, tonglen, and visualization) during meditation versus a mind-wandering control task, and to test whether meditative states differ neurophysiologically regardless of technique. | 28 | 50 | 52.32 | 15.74 | EEG | Buddhism | Meditation | Six meditation styles were studied: shamatha (calm abiding, focused attention on breath), vipassana (insight meditation, body scanning), zazen (seated Zen meditation, shikantaza), dzogchen (Tibetan "great perfection" non-dual awareness practice), tonglen (compassion-based visualization practice), and deity visualization (Vajrayana-style visualization of oneself as a deity) | Widespread scalp regions using 16 electrodes (Fp1, Fp2, F3, F4, F7, F8, C3, C4, T3, T4, T5, T6, P3, P4, O1, O2); exploratory analyses noted the largest alpha power increases in tonglen and zazen groups across most electrodes and regions | Entropy (Lempel-Ziv complexity) was significantly lower during meditation than during mind-wandering across all six practices combined, suggesting that meditation, regardless of specific technique, reduces neural complexity. In contrast, there were no significant differences in power spectra between meditation and mind-wandering when averaged across all frequency bands and practices, likely due to the heterogeneity of practices studied. Exploratory analyses of individual meditation styles found that tonglen and zazen participants showed the largest significant increases in alpha power across many electrode sites, while other practices showed more variable, non-significant patterns. | Meditation reduces neural complexity (entropy) compared to mind-wandering regardless of the specific style of meditation practiced, indicating that reduced entropy may be a general marker of meditative states. However, power spectra findings were less consistent, likely reflecting genuine neurophysiological differences between distinct meditation practices rather than a single unified "meditation" signature. |
| 38 | (Avvenuti et al., 2020) | 2020 | To investigate, using a longitudinal design combining psychometric questionnaires with structural and resting state functional MRI, whether short term Transcendental Meditation (TM) practice in naive subjects produces measurable brain structural and functional modifications, particularly within the default mode network (DMN), that underlie its psychological effects on stress and anxiety. | 34 (19 meditators, 15 controls) | 52.9 | 29 (meditators), 32 (controls) | 9 (meditators), 11 (controls) | MRI | Not Specified |  | Transcendental Meditation | Posterior cingulate cortex (PCC), precuneus, left superior parietal lobule, right insula | After 3 months of TM practice, meditators (but not controls) showed a significant reduction in a composite score reflecting depression, anxiety, and stress. This psychological improvement was negatively correlated with increased functional connectivity between the PCC and both the precuneus and left superior parietal lobule, regions within the posterior DMN. Additionally, TM practice was associated with a significant increase in functional connectivity between the PCC and right insula, though this was not correlated with psychological changes and may instead reflect shifts in interoceptive awareness. No significant structural (cortical thickness) changes were found in either group. | Short term Transcendental Meditation practice is associated with functional (but not structural) reorganization within the posterior default mode network and between the DMN and interoceptive regions such as the insula, and these connectivity changes are linked to reductions in perceived stress and anxiety. This suggests that even a brief period of mantra based meditation can produce measurable neuroplastic changes supporting emotional well-being, adding to the neurotheological evidence that contemplative and spiritually rooted practices can modulate large scale brain networks involved in self-referential processing and interoceptive awareness. |
| 39 | (Barcelona et al., 2020) | 2020 | To explore whether contemplative prayer is associated with EEG brain activity patterns consistent with established meditation signatures, and to examine frontal alpha asymmetry as a function of age in practising Catholic sisters. | 33 | 100 | 80.1 | 8.3 | EEG | Christianity | Contemplative Prayer | Contemplative / Meditative Prayer | Posterior-occipital region O1, O2, Oz, P4, P8, PO3, PO4 Frontal region (prefrontal cortex) - specifically F3 (left frontal) and F4 (right frontal) electrodes for Frontal Alpha Asymmetry (FAA) analysis The left frontal-central activation | Greater alpha power was detected in posterior-occipital regions during prayer relative to resting state. Frontal alpha asymmetry values became increasingly positive with age in both prayer and rest conditions (r ≈ 0.43, p < 0.02), indicating enhanced approach-related emotional processing in older participants. Ninety percent of participants exhibited positive frontal alpha asymmetry values, reflecting left-frontal predominance. No significant difference in frontal alpha asymmetry was found between praying and resting conditions. | Contemplative prayer is associated with posterior-occipital alpha augmentation consistent with relaxed, selective attentional states reported in mindfulness-based meditation research. Age-related increases in approach-oriented frontal alpha asymmetry during prayer suggest that sustained contemplative practice contributes to a neurophysiological trait associated with psychological wellbeing and positive affect. |
| 40 | (Dobrakowski et al., 2020) | 2020 | To investigate whether Buddhist meditation and Christian prayer differentially impact alpha and theta electrophysiological activity, and to determine whether these effects are moderated by individual levels of spiritual transcendence. | 36 | 47 |  |  | EEG | Mixed / Multiple Religions | Prayer | Meditation | Cz (Central area, above central sulcus), Pz (Parietal area, medial), Frontal areas (Fp1, Fp2, Fz) No significant differences , O1, O2 (Occipital)No significant differences. | Buddhist meditators exhibited significantly higher theta amplitude at the central scalp site (Cz) during meditation relative to relaxation (d = 0.57, p = 0.045). Spiritual openness (a subscale of spiritual transcendence) was associated with greater theta amplitude at the parietal site (Pz) during relaxation in Buddhist meditators. A significant interaction effect was identified for group × measurement level × spiritual openness on parietal theta (F(2,17) = 6.61, p = 0.008, η² = 0.44). No significant between-group or main-effect differences were found in frontal regions. | Buddhist meditation produces verifiable theta augmentation in central cortical areas, with parietal theta amplitude modulated by trait-level spiritual openness. The interaction between group, practice condition, and spiritual disposition underscores the importance of individual differences in contemplative predisposition on the neurophysiological outcomes of meditative practice. |
| 41 | (Gao et al., 2020) | 2020 | To investigate the neural mechanisms by which repetitive religious chanting regulates negative emotional responses to fear-eliciting stimuli, using a multi-modal MRI paradigm combining functional neuroimaging and structural morphometry. | 21 | 52 |  |  | fMRI; MRI | Buddhism | Chanting | Devotional Chanting | amygdala, thalamus, pons, prefrontal cortex, hippocampus, parahippocamous, cerebellum, fusiform gyrus | Religious chanting produced significantly stronger brain activation than non-religious chanting and no-chanting conditions when viewing fearful images. The left amygdala exhibited significantly higher activation than the right amygdala during religious chanting. Activated regions included the fusiform gyrus, prefrontal cortex, thalamus, amygdala, midbrain, and cerebellum. Long-term practitioners demonstrated rightward structural asymmetry in the thalamus, putamen, hippocampus, and amygdala. | Religious chanting activates a distributed network sustaining positive emotional schemas that functionally counteract fear processing through amygdala engagement, prefrontal modulation, and thalamo-cerebellar coordination. Long-term practice produces lateralized structural plasticity in subcortical emotion-regulation structures, consistent with experience-dependent neuromorphological adaptation. |
| 42 | (Imperatori et al., 2020) | 2020 | To investigate the electroencephalographic (EEG) correlates of positive religious coping with psychological stress using quantitative spectral EEG analysis, specifically examining whether religiosity-linked coping is reflected in distinct neurophysiological signatures. | 96 | 55 | 22.3 | 2.48 | EEG | Christianity | Prayer | Meditation | Right hemisphere frontal and temporal areas: Superior temporal gyrus (BA 38), Inferior frontal gyrus (BA 47), Middle temporal gyrus (BA 21) | Religious coping scores were significantly positively correlated with theta power in right frontal and temporal regions, specifically the superior temporal gyrus (BA 38), inferior frontal gyrus (BA 47), and middle temporal gyrus (BA 21). This association remained significant after controlling for sex, age, active coping, and positive reframing strategies. No significant associations were identified in other frequency bands (delta, alpha, beta, gamma). | Positive religious coping is electrophysiologically characterized by elevated right-hemisphere theta activity in frontal and temporal regions. As decreased frontal-temporal activity constitutes a pathophysiological feature of depression and post-traumatic stress disorder, the observed theta augmentation may represent a neurophysiological mechanism underlying the established protective effects of religious coping on mental health outcomes. |
| 43 | (Gao et al., 2019) | 2019 | To use multi modal electrophysiological (EEG) and neuroimaging (fMRI) methods to investigate the neural correlates of religious chanting (repetitive chanting of the name of Amitabha Buddha), comparing it against both a resting state and an active, matched non-religious chanting control condition. | 23 [22 for the EEG/ECG study (21 usable EEG datasets after one exclusion), plus 1 additional participant for the separate fMRI case study] | 54.5 for the EEG sample (12 of 22); the additional fMRI participant was also female | 46.5 (EEG sample); the fMRI participant was 43 | 2.6 (EEG sample) | EEG; fMRI | Buddhist | Chanting |  | Posterior cingulate cortex (PCC), identified independently by both fMRI (largest decrease in eigenvector centrality) and EEG independent component clustering (localized delta band source) | During religious chanting compared to non-religious chanting (chanting "Santa Claus" as a linguistically matched control), the PCC showed the largest decrease in eigenvector centrality on fMRI in a highly trained meditator, and EEG data from an independent sample of practitioners showed a corresponding cluster in the PCC with significantly increased delta band (1 to 4 Hz) power during religious chanting relative to non-religious chanting. No significant differences appeared in theta, alpha, beta, or gamma bands. Compared to the no chanting resting state, religious chanting was associated with lower HRV total power, lower high frequency HRV power, and lower very low frequency HRV power, indicating increased cardiac stability; these autonomic effects did not differ between religious and non-religious chanting, ruling out cardiac confounds for the delta band finding. No significant differences in respiration were found across conditions. | Religious chanting of Amitabha Buddha increases endogenous delta band oscillations concentrated in the posterior cingulate cortex, a region central to the default mode network and to self-referential processing, and this effect appears distinct from mechanisms reported for both mindfulness meditation and other forms of religious prayer, which have more typically been linked to increased alpha and theta activity. |
| 44 | (Li et al., 2019) | 2019 | To examine brain microstructural differences in white matter associated with familial risk for major depressive disorder, and to test whether high importance of religion and spirituality normalises these structural connectivity differences in at-risk offspring. | 99 | 54 | 33.45 | 13.48 | DTI | Mixed / Multiple Religions |  | Intrinsic Religiosity And Spirituality | Precuneus, Superior parietal lobule, Superior frontal gyrus, Middle frontal gyrus, Bilateral insula, Supplementary motor area, Postcentral gyrus, Thalamus (bilateral), Superior temporal gyrus, Inferior temporal gyrus, Middle temporal gyrus, Inferior frontal gyrus, Middle occipital gyrus, Paracentral lobule, Precentral gyrus, Cuneus, Medial frontal gyrus | In individuals for whom religion/spirituality was of low importance, high-risk individuals showed significantly decreased ellipsoidal area ratio (EAR) in white matter neighboring the precuneus, superior parietal lobe, superior and middle frontal gyrus, bilateral insula, supplementary motor area, and postcentral gyrus. In those for whom religion/spirituality was of high importance, risk-associated microstructural differences in the precuneus, frontal lobe, and temporal lobe were substantially diminished or non-significant. These findings suggest that religion/spirituality importance partially normalizes the microstructural white matter signature of depressive familial risk. | High importance of religion and spirituality beliefs is associated with a neurobiologically protective pattern of white matter integrity, attenuating the microstructural connectivity differences that otherwise characterize familial vulnerability to major depressive disorder. These findings implicate spirituality as a moderator of structural neural resilience in depression-prone populations. |
| 45 | (McClintock et al., 2019) | 2019 | To use spatial independent component analysis (sICA) on fMRI data from a personalized guided imagery task to identify functional brain networks specifically linked to spiritual experiences (compared with stressful and neutral relaxing conditions), building on prior region-based findings from the same dataset. | 27 | 44.4 | 22.4 |  | fMRI | Mixed |  | Personally meaningful spiritual experiences | A ventral frontotemporal network including the middle and inferior frontal cortices, superior, middle, and inferior temporal cortices, insula, frontal opercula, striatum (caudate, putamen, pallidum), thalamus, brainstem, and cerebellum, with concurrent negative signals in the middle and posterior cingulate cortex and inferior parietal lobule (IPL) | Spatial independent component analysis (sICA) identified a ventral frontotemporal network that was engaged during spiritual imagery in a pattern distinct from stressful or neutral relaxing imagery. This network showed a significant main effect of imagery condition (F(1.4, 37.4) = 7.69, p = .004) and a block by condition interaction (F(6, 156) = 3.59, p = .002), with greater engagement during spiritual imagery relative to both stress (p = .016) and neutral relaxing (p = .004) conditions. The network was positively engaged at the start of all conditions but remained positively engaged through the end only for spiritual imagery, while becoming negative or neutral by the end of stress and neutral relaxing imagery. Change in network engagement correlated with self-reported spirituality ratings during the task (r = .71, p < .001) and with scores on an independent Spirituality Scale (r = .48, p = .018). | Spiritual experiences engage a distributed ventral frontotemporal network resembling the ventral attention network, along with subcortical regions tied to reward and emotion (striatum, thalamus, brainstem) and concurrent deactivation in self-referential regions (posterior cingulate cortex and inferior parietal lobule). This pattern suggests spiritual experiences involve involuntary attentional reorientation, a more expanded or less bounded sense of self, and positive emotional processes related to bonding or attachment. |
| 46 | (Miller et al., 2019) | 2019 | To investigate the neural correlates of personally meaningful spiritual experiences, as compared with stressful and neutral relaxing experiences, using an individualized guided imagery task and fMRI. | 27 | 44.4 | 22.3 |  | fMRI | Mixed |  | Personalized spiritual experiences | Left inferior parietal lobule (IPL), medial thalamus, caudate, and precuneus | Participants reported significantly higher spiritual connection following the spiritual scripts compared to both stress and neutral relaxing scripts (F(2,52) = 23.45, p < 0.05). Anxiety was significantly higher following stress scripts compared to spiritual or neutral relaxing scripts (F(2,52) = 28.98, p < 0.05). In fMRI results, the spiritual condition showed reduced activity in the left IPL relative to the neutral relaxing condition. Relative to the stress condition, the spiritual condition showed reduced activity in the medial thalamus and striatum. The stress condition, compared to neutral relaxing, showed greater activity in the bilateral thalamus and caudate, alongside reduced activity in the right temporal lobe, ventral striatum extending into orbitofrontal cortex, and bilateral precuneus extending into the IPL. | Personally meaningful spiritual experiences are associated with reduced activity in the left IPL, a region tied to perceptual processing and self-other representation, supporting the idea that spiritual experiences may involve a perceived encounter with a presence or entity external to oneself. Reduced activity in the medial thalamus and striatum during spiritual (versus stress) conditions suggests that both spiritual and relaxing states differ from stress in how these subcortical regions engage. |
| 47 | (Chen et al., 2018) | 2018 | To clarify how long-term meditation in Sant Mat practitioners alters amygdala activity during explicit and implicit emotional processing (fear and happiness), whether this modulation depends on positive or negative emotional valence, and how amygdala reactivity relates to self-reported anxiety and functional connectivity with other brain regions. | 41 (21 meditators and 20 novices) | 61% overall (14 of 21 meditators were female, 12 of 20 novices were female) | 52.43 years (meditators), 49.7 years (novices) | 7.85 (meditators), 8.2 (novices) | fMRI | Sant Mat (a spiritual movement described as reflecting the teachings of saints and mystics, sometimes associated with Sikh-related or Radhasoami traditions) |  | Sant Mat meditation | Amygdala (bilateral, with primary focus on the right amygdala), ventrolateral prefrontal cortex, medial orbitofrontal cortex, insula, superior temporal gyrus, thalamus/midbrain, anterior cingulate cortex, lingual gyrus, precuneus, parahippocampus, hippocampus, cuneus, and medial prefrontal cortex | Meditators reported significantly lower state and trait anxiety scores (STAI) than novices, and years of meditation experience were negatively correlated with both state and trait anxiety. During explicit emotional processing, meditators showed increased amygdala reactivity to happiness compared to fear, while novices showed the opposite pattern. During implicit (masked) emotional processing, meditators showed significantly weaker amygdala reactivity than novices regardless of fear or happiness. Meditators also showed stronger positive functional connectivity between the amygdala and ventrolateral prefrontal cortex during explicit happiness, and more negative connectivity between the amygdala and both the medial orbitofrontal cortex and bilateral insula during explicit fear. Mediation analysis showed that amygdala reactivity during explicit fear processing mediated the relationship between years of meditation experience and reduced trait anxiety. | Long-term Sant Mat meditation, which combines loving-kindness meditation with a vegetarian, alcohol-restricted lifestyle, is associated with reduced anxiety and altered amygdala reactivity and functional connectivity during both explicit and implicit emotional processing. The amygdala serves as a neural mediator linking meditation experience to reduced trait anxiety, supporting the idea that long-term meditation practice is functionally coupled with emotional processing circuits in ways that may enhance well-being. |
| 48 | (Ferguson et al., 2018) | 2018 | To identify the neural substrates of subjective religious and spiritual experience ("feeling the Spirit") in devout Mormons, testing whether reward-related brain circuits (particularly the nucleus accumbens) are reproducibly activated during self-identified spiritual feelings across multiple experimental paradigms. | 19 | 36.8 | 27.42 | 3.63 | fMRI | Christianity | Prayer, scripture reading (Book of Mormon passages), listening to quotations from LDS and non-LDS religious authorities, viewing religiously themed audiovisual content | Self-identified experience of "feeling the Spirit" | Bilateral nucleus accumbens (ventral striatum), ventromedial prefrontal cortex, anterior cingulate cortex, supplementary motor area, and right frontal eye field | Self-reported peak spiritual feelings were consistently associated with activation in the nucleus accumbens across four separate task acquisitions, with nucleus accumbens activity peaking 1 to 3 seconds before participants indicated peak spiritual feeling by button press. Frontal attentional regions (anterior cingulate cortex, supplementary motor area, frontal eye field) showed activation that was more pronounced in the right hemisphere. Ventromedial prefrontal cortex activation was observed across all three quotation and scripture tasks. Subjective ratings of "feeling the Spirit" correlated positively with heart rate and negatively with respiratory rate. | Religious and spiritual experience in devout Mormons recruits classical reward circuitry (nucleus accumbens) in coordination with frontal attentional networks and regions implicated in valuation and meaning-making (ventromedial prefrontal cortex), suggesting that spiritual feelings are supported by domain-general neural systems for reward, salience, and attention rather than a religion-specific brain mechanism, and that this reward-attention coupling may underlie the motivational power of religious doctrine and adherence to religious authority. |
| 49 | (Gupta et al., 2018) | 2018 | To compare functional imaging (resting state fMRI and SPECT brain perfusion) and neuropsychological changes in healthy individuals following a specific spiritual practice intervention versus a physical exercise control intervention, in order to evaluate the neural and cognitive correlates of spiritual practice induced transformation. | 14 | 0 |  |  | MRI; SPECT | Not specified |  | ShaktiPravaah (guided energy transfer practice) | Left precuneus and posterior cingulate cortex, right cuneus, bilateral frontal lobes, left anterolateral frontal lobe, parietal lobes | The spiritual practice group showed increased resting state functional connectivity of the left precuneus within the default mode network and decreased connectivity of the right cuneus, along with significant improvement in speed of processing (Digit Symbol Test) and significantly increased frontal lobe perfusion on SPECT, with lesser change in parietal perfusion. The exercise control group showed increased connectivity in the left anterolateral frontal lobe, significant improvement in focused attention (Trail Making A), and increased parietal lobe perfusion with relatively less frontal change. Only the spiritual practice group reported significant subjective improvements in mood, general well being, feelings of calm and reduced anxiety, and interpersonal and occupational functioning, while the exercise group reported improved attention and general health without mood changes. No significant changes were found on the Daily Spiritual Experience Scale in either group. | A structured spiritual practice involving guided energy transfer and daily meditation produces distinct patterns of brain network connectivity and perfusion, centered on the precuneus and posterior cingulate cortex within the default mode network and on frontal lobe activation, alongside improvements in processing speed and subjective mood and well being, that are dissociable from the brain and cognitive changes produced by physical exercise alone (which instead engaged frontal executive networks and parietal perfusion tied to focused attention). |
| 50 | (Harne & Hiwale, 2018) | 2018 | To investigate the temporal dynamics of EEG oscillatory changes before and after 30 minutes of loud OM mantra chanting in naive meditators, testing whether OM chanting produces specific frequency band changes similar to those reported in other meditation traditions. | 23 | 60.9 | 20.99 | 0.99 | EEG | Hinduism | Chanting |  | Ten scalp regions across frontal, temporal, occipital, parietal, and central areas bilaterally (left and right hemispheres), based on 16 electrode EEG recording; no single region was implicated over others given the whole brain, band level nature of the theta effect | A single 30 minute session of loud OM chanting produced a significant overall increase in relative theta power averaged across all brain regions after chanting compared to before, with no significant condition main effect in delta, alpha, beta, or gamma bands. The theta increase occurred broadly across all ten scalp regions without a specific regional or hemispheric localization pattern reaching significance in post hoc tests. No band by condition interaction was found, indicating no single band, other than theta as a main effect, was specifically tied to the chanting intervention. | Even a brief, single session of loud OM chanting by completely naive practitioners is sufficient to produce a measurable increase in theta band EEG activity across the brain, a pattern that mirrors findings from more experienced practitioners of other meditation traditions and is generally interpreted as reflecting reduced cortical arousal and a state of relaxation. This suggests that OM mantra chanting can rapidly induce neurophysiological changes associated with relaxation even without prior training, supporting its potential as an accessible introductory practice. |
| 51 | (Khanam et al., 2018) | 2018 | To investigate human brain activities related to EEG signals during psychophysiological activities in Salat (Muslim prayer), comparing alpha relative power (RP) during Salat with two relaxed conditions (eyes open and eyes closed). | 5 | 0 | 23 | 6 | EEG | Islam | Prayer (Salat) |  | Frontal lobe (Fz, F3, F4), central lobe (Cz, C3, C4), parietal-occipital region (POz, P3, P4) | Alpha relative power was significantly higher (p < 0.05) during Salat compared to both eyes-open and eyes-closed relaxed states in six of nine channels, specifically the frontal (Fz, F3, F4) and parietal (POz, P3, P4) regions. Alpha RP also increased progressively over the course of the Salat performance. A comparison with a related study using a different protocol (before, during, and after Salat) showed a similar pattern of increased alpha RP during Salat in most electrode sites, with the central lobe as an exception attributed to protocol differences. | Salat produces a greater relaxed mental state than ordinary resting conditions (eyes open or eyes closed), as indicated by higher alpha activity, particularly in the frontal and parietal regions. This alpha increase reflects heightened parasympathetic activity and reduced sympathetic activity, supporting the conclusion that regular practice of Salat may help reduce anxiety and provide psychological relief. |
| 52 | (Newberg et al., 2018) | 2018 | To determine whether a seven-day Ignatian spiritual retreat produces measurable changes in dopamine transporter (DAT) and serotonin transporter (SERT) binding as assessed by DaTscan SPECT, and to correlate neurochemical changes with validated psychological and spiritual outcome measures. | 14 | 43 | 54 | 13 | SPECT | Christianity | Spiritual Retreat, meditation, prayer |  | basal ganglia, midbrain | The retreat produced significant decreases in dopamine transporter binding in the basal ganglia (caudate and putamen) and significant decreases in serotonin transporter binding in the midbrain. Participants demonstrated significant improvements in tension, fatigue, physical health perception, self-transcendence, religiosity, and spirituality measures. Increased self-transcendence scores correlated significantly with decreased DAT binding in the right caudate and right putamen. | An intensive week-long spiritual retreat involving Ignatian contemplative exercises produces neurochemical modifications in basal ganglia dopaminergic and midbrain serotonergic systems. These changes; particularly the correlation between self-transcendence and striatal DAT reductions; suggest that structured spiritual retreats modulate reward and affective neuromodulatory systems in a manner analogous to evidence-based mindfulness interventions. |
| 53 | (Rao et al., 2018) | 2018 | To investigate the directional (effective) brain connectivity underlying deactivations observed during OM chanting, by re-analyzing fMRI data using multivariate autoregressive (MVAR) modeling based Granger causality, to determine which deactivated regions drive communication with limbic structures such as the amygdala. | 12 | 25 | 28 | 6 | fMRI | Not Specified | Chanting |  | Insula (bilateral), anterior cingulate cortex, orbitofrontal cortex, amygdala (right), parahippocampal gyri, hippocampi, thalami | During OM chanting, directional connectivity (outputs) from the insula, anterior cingulate cortex, and orbitofrontal cortex was significantly reduced compared to both rest and the SSSS control condition, with no paths showing significantly increased connectivity. Notably, output specifically from these regions to the amygdala, a key limbic structure, was reduced. No significant connectivity differences were found between the SSSS condition and rest, suggesting the observed reductions were specific to OM chanting rather than general vocalization or repetition effects. Deactivation during OM chanting was not accompanied by increased activation elsewhere, unlike patterns seen in typical attention tasks. | Chanting is associated with reduced directional communication from insular, anterior cingulate, and orbitofrontal regions to limbic areas including the amygdala, suggesting that this traditional yogic vocal practice modulates neural circuits involved in emotion processing and self-referential processing. Given that these same circuits show aberrant connectivity in major depressive disorder, the findings point to a potential neurobiological mechanism by which sustained spiritual chanting practices could exert therapeutic effects on mood regulation, though the small sample and heterogeneous meditation experience among participants limit generalizability. |
| 54 | (Sulianti et al., 2018) | 2018 | To determine the influence of dhikr (Islamic devotional remembrance practice) on epileptiform activity in a teenager with comorbid depressive disorder, using EEG and clinical observation. | 1 | 100 | 16 |  | EEG | Islam | Dhikr (remembrance of Allah) |  | Whole scalp EEG recording | Following four weeks of routine dhikr practice, the subject's depressive disorder score on the HAM-D 17 dropped sharply from 28 (very severe depression) to 11 (mild depression). Epileptic seizure frequency decreased over the observation period, while seizure endurance (resistance to seizure triggers such as puddles, blood, and stress) increased. By day 20, the subject no longer had seizures when exposed to a rainwater puddle trigger, and by day 25, she did not seize even when exposed to blood, a previously reliable trigger. The EEG record on day 25 showed no artifact signs of seizure activity even when the subject was given a stressor (a mathematics task), which had previously provoked seizure related EEG changes. | Dhikr intervention was associated with a major reduction in depressive disorder severity and epileptic seizure frequency in a teenage subject with epilepsy and severe comorbid depression, along with increased resistance to seizure triggers. |
| 55 | (Travis et al., 2018) | 2018 | To test whether the Transcendental Meditation (TM) program could increase EEG brain integration and positive affect, and decrease psychological distress, in government employees, using a randomized controlled design. | 96 (88 completed pretest and posttest POMS; 79 in the EEG subgroup, with 69 completing both pretest and posttest EEG) | 82% (control group), 80% (TM group) | 45.5 (control), 45.8 (TM) for the POMS sample; 46.6 (control), 45.5 (TM) for the EEG subgroup | 9.5 (control), 11.2 (TM) for the POMS sample; 9.8 (control), 10.7 (TM) for the EEG subgroup | EEG | Not Specified |  | Transcendental Meditation (TM) | Frontal cortex (F3, F4), central region (Cz, C3, C4), parietal region; medial frontal and anterior cingulate cortices (referenced from related MEG research) | Compared to wait-list controls, TM participants significantly increased their Brain Integration Scale (BIS) scores (p = .044, effect size d = 0.30). TM participants also significantly decreased on POMS Total Mood Disturbance and the anxiety, anger, depression, fatigue, and confusion subscales (all p < .001), and significantly increased on the vigor subscale (p < .001), with effect sizes ranging from moderate to large (d = 0.58 to 0.99). Compliance with the meditation practice was high (93%). Correlations between BIS changes and POMS subscale changes were in the predicted direction but did not reach statistical significance. | The TM program is a feasible and effective workplace intervention that can improve brain integration and positive affect while reducing psychological distress (mood disturbance, anxiety, anger, depression, fatigue, and confusion) among administrators and staff. The low correlation between BIS and POMS changes suggests brain integration and mood states may reflect different, though related, levels of psychological functioning; brain integration may represent a broader supportive context for more localized emotional processing. |
| 56 | (Elmholdt et al., 2017) | 2017 | To investigate whether religious prayer attenuates pain perception and to identify the underlying neural mechanisms, including whether analgesic effects are opioid-mediated, using a double-blind, naloxone-controlled fMRI paradigm. | 28 | 57 | 24 |  | fMRI | Christianity | Prayer | Personal Prayer | Parietofrontal network | Religious prayer reduced pain intensity ratings by 11% and pain unpleasantness ratings by 26% relative to secular prayer. Analgesic effects were not blocked by naloxone, indicating non-opioid mediation. Neuroimaging revealed reduced BOLD signal in parietofrontal attentional and executive control networks during religious prayer. Expectancy accounted for 70–89% of variance in subjective pain ratings. | Religious prayer produced non-opioid analgesia associated with decreased activation of parietal and frontal regions implicated in attentional salience processing and pain-related cognitive evaluation. These findings suggest that devotional prayer modulates the affective-motivational dimension of pain experience through expectancy-driven top-down mechanisms. |
| 57 | (Galanter et al., 2017) | 2017 | To delineate the experiential and neural correlates of diminished alcohol craving following Alcoholics Anonymous (AA) prayer in members with long-term abstinence, using functional magnetic resonance imaging. | 18 | 44 | 52.39 | 13.75 | fMRI | Christianity | Prayer |  | Left-anterior middle frontal gyrus (BA 10), left superior parietal lobule (BA 7), bilateral precuneus (BA 7), bilateral posterior middle temporal gyrus (BA 39), right intraparietal sulcus (BA 7), bilateral lingual gyrus (BA 18), left caudate nucleus, right insula (BA 13), right inferior prefrontal gyrus (BA 47), right supramarginal gyrus (BA 40), bilateral posterior cingulate gyrus (BA 23), bilateral supramarginal gyrus (BA 39), right anterior middle prefrontal gyrus (BA 10), right medial frontal gyrus (BA 9). | Prayer was associated with significant reductions in self-reported craving and increased BOLD activation in the left anterior middle frontal gyrus (BA 10), left superior parietal lobule (BA 7), bilateral precuneus (BA 7), and bilateral posterior middle temporal gyrus (BA 39). Craving ratings following prayer were inversely correlated with activation in areas subserving self-referential processing and default mode network function. Prayer selectively engaged both top-down (voluntary attention, goal maintenance) and bottom-up (salience detection, interoception) control systems in an integrative manner. | Increased activation within attentional and cognitive control networks, with concurrent suppression of default mode network activity. The inverse relationship between prayer-related neural activation and craving suggests that engagement of self-referential and executive processing circuitry mediates craving reduction. |
| 58 | (Gao et al., 2017) | 2017 | To investigate the neural mechanisms underlying the effects of religious chanting (specifically chanting Amitābha Buddha) on the brain's response to fear- and stress-provoking pictures, and to examine whether religious practice modulates early-stage (perceptual) versus late-stage (emotional/cognitive) processing of negative stimuli. | 21 | 48 |  |  | EEG; ECG; GSR | Buddhism | Chanting | Devotional Buddha Chanting | Parietal lobe (specifically central-parietal regions) | Viewing negative pictures increased N1 amplitude (early perceptual processing) in ALL chanting conditions, showing no effect of chanting on early-stage processing Increased Late Positive Potential (LPP) was observed when viewing negative pictures under no-chanting and Santa Claus conditions, but NOT when chanting Amitābha Source analysis confirmed increased LPP originated mainly from central-parietal regions Heart rates decreased significantly when viewing negative pictures in the Santa Claus condition, but showed no significant difference between negative and neutral pictures when chanting Amitābha Chanting Amitābha modulates late-stage emotional/cognitive processing but not early perceptual processing of negative stimuli | Chanting Amitābha reduced the Late Positive Potential (LPP) amplitude when viewing negative pictures, indicating reduced late-stage emotional/cognitive processing of negative stimuli. Source analysis showed reduced activation in the parietal lobe during emotional processing. However, chanting did not affect the early N1 component (early perceptual processing remained intact). |
| 59 | (Kober et al., 2017) | 2017 | To investigate whether prayer frequency is associated with superior volitional control over one's own brain activity during EEG neurofeedback training, and to examine structural brain differences that may underlie this capacity. | 40 | 55 | 23.93 | 1.07 | fMRI; EEG | Christianity | Prayer |  | Right insula & inferior frontal gyrus, Left postcentral gyrus, Left medial orbitofrontal cortex | High-frequency prayer participants (HF) demonstrated significantly better neurofeedback performance than low-frequency prayer participants (LF). HF participants exhibited more effective suppression of stimulus-independent thoughts and task-irrelevant information. Neuroimaging data indicated that HF participants relied on the medial orbitofrontal cortex for distractor suppression (effortless strategy), whereas LF participants recruited the insula and inferior frontal gyrus (effortful strategy). | Regular prayer practice is associated with enhanced volitional neural self-regulation, as indexed by superior EEG neurofeedback performance. Habitual prayer appears to strengthen the medial orbitofrontal cortex as a substrate for effortless attentional gating, paralleling the neural efficiency profile observed in experienced meditators. |
| 60 | (Viol et al., 2017) | 2017 | To test the entropic brain hypothesis in the context of ayahuasca by measuring Shannon entropy of brain functional complex networks from resting-state fMRI data obtained before and after ritualistic ayahuasca consumption. | 10 |  | 31.3 |  | fMRI | Santo Daime | Ayahuasca Ritual | Ayahuasca-Induced Altered State Of Consciousness | 104 brain regions from Harvard-Oxford atlas covering whole brain | Ayahuasca ingestion produced increased Shannon entropy of the degree distribution, increased local network integration, and decreased global network integration. Degree distributions became less peaked and wider, indicating a more heterogeneous functional connectivity architecture. These findings are consistent with a shift from an adult, trained, constrained mode of consciousness toward more primary, unconstrained information processing. | Ayahuasca disrupts the hierarchical constraints of ordinary waking network organization, increasing the entropy and local integration of functional connectivity while reducing global network efficiency. This neurodynamic reorganization; consistent with the entropic brain hypothesis; may underlie the expanded and undirected quality of psychedelic and spiritual conscious states. |
| 61 | (Baldwin et al., 2016) | 2016 | To examine the neural correlates of a healing prayer intervention (Psycho-Spiritual Healing Forgiveness; PSFH) on major depressive disorder, using fMRI to identify brain activity changes associated with processing traumatic memories before and after the prayer intervention. | 14 | 79 | 45.6 | 13 | fMRI | Christianity | Prayer | Psycho-Spiritual Healing Forgiveness | Medial Prefrontal Cortex (mPFC), Left Inferior Frontal Gyrus (IFG), Precuneus | Hamilton Depression Rating Scale scores declined significantly from 21.6 to 4.0 post-intervention, with sustained improvement (3.7) at 12-month follow-up. Neuroimaging revealed increased activity in the medial prefrontal cortex when focusing on traumatic memories following the prayer intervention. Increased left inferior frontal gyrus activation correlated with degree of depressive symptom improvement. Decreased precuneus activation was observed when focusing on negative affect post-intervention. | Healing prayer intervention modifies the neural processing of traumatic memories through increased medial prefrontal engagement; consistent with reappraisal and emotion regulation; alongside precuneus deactivation, reflecting reduced affective and self-referential engagement with negative memory content. Left inferior frontal gyrus engagement appears to mediate the relationship between prayerful processing and symptomatic improvement in depression. |
| 62 | (Doufesh et al., 2016) | 2016 | To examine differences in EEG gamma activity between actual Salat (Islamic prayer incorporating recitation and focused concentration) and mimic Salat (physical movements without recitation or concentration) across all prayer stages. | 20 | 0 | 26 | 5 | EEG | Islam | Prayer |  | Frontal region (prefrontal cortex),Parietal region, Left hemisphere, and Central-parietal region | Gamma power during actual Salat was significantly higher than during mimic Salat in frontal and parietal regions across all prayer stages. Left-hemisphere gamma power was significantly greater across all cerebral regions and stages, with the exception of the central-parietal region during sitting and the frontal area during bowing. Prostrating and bowing postures produced the greatest gamma power elevations. These findings are consistent with Salat functioning as a focused attention meditation. | Actual Salat, incorporating verbal recitation and deliberate concentration, produces enhanced gamma-band activity in frontal and parietal regions consistent with heightened attentional processing and working memory engagement. Left-hemispheric predominance of gamma activity suggests particular involvement of language and semantic networks during recitation-integrated prayer. |
| 63 | (Svob et al., 2016) | 2016 | To examine whether the personal importance of religion and spirituality moderates the relationship between default mode network (DMN) connectivity and familial risk for major depressive disorder. | 104 |  |  |  | fMRI |  |  | Intrinsic Religiosity And Spirituality | Left lateral parietal lobe (inferior portion) | Greater importance of religion/spirituality was associated with decreased DMN connectivity in the left lateral parietal lobe; an effect that was specific to high-familial-risk individuals. This protective moderation was not observed in low-risk participants and was not replicated for DMN-central executive network inverse connectivity, indicating specificity to intra-DMN functional architecture. | Religion and spirituality importance is associated with a normalizing reduction of left lateral parietal DMN connectivity in individuals at elevated familial risk for depression. This pattern may represent a neurobiological mechanism through which spiritual engagement confers resilience against the pathological DMN hyperconnectivity associated with depressive vulnerability. |
| 64 | (Newberg et al., 2015) | 2015 | To measure changes in cerebral blood flow using SPECT imaging during two types of intense Islamic prayer practices (Dhikr and salat), testing the hypothesis that surrender based prayer practices centered on connectedness with God would show decreased activity in the prefrontal cortex, parietal lobes, and sensory regions, distinct from the increased frontal activity typically seen in concentrative meditation practices. | 3 (case series; 2 American Sufi practitioners of Dhikr, 1 Muslim practitioner of salat) | 33.3 |  |  | SPECT | Islam | Prayer | Dhikr | Prefrontal cortex, superior and inferior frontal lobes, orbitofrontal cortex, superior parietal lobe, sensory strip, visual cortex, and temporal lobe regions (decreased activity); anterior cingulate gyrus, caudate nucleus, insula, thalamus, dorsal medial cortex, and globus pallidus (increased activity, primarily during the more intense salat prayer) | Both Dhikr and salat prayer practices were associated with generally decreased cerebral blood flow in frontal, parietal, and sensory or visual cortical regions relative to baseline or automatic prayer, consistent with a surrender based rather than concentrative pattern of brain activity. The more intense form of salat additionally showed increased activity in the anterior cingulate gyrus, caudate nucleus, insula, thalamus, and globus pallidus, regions associated with reward, emotional regulation, and interoception, whereas these same regions showed decreased activity during Dhikr. All participants subjectively reported intense experiences of surrender and connectedness to God, with the deliberately intensified salat producing a greater subjective sense of surrender than the automatic version. Default mode network structures, including temporal lobe, prefrontal cortex, posterior cingulate cortex, precuneus, and inferior parietal cortex, generally showed decreased activity during the prayer practices. | Intense Islamic prayer practices characterized by surrender to God, whether performed as Sufi Dhikr or as deeply focused salat, are associated with decreased prefrontal, parietal, and sensory cortical activity, a pattern distinct from the increased frontal activation typically observed during concentrative meditation practices that emphasize willful focus on an object or phrase. The additional recruitment of reward, emotion regulation, and interoceptive regions (caudate, insula, thalamus, anterior cingulate) during the more intense, deliberately surrendered prayer state suggests that the subjective depth of religious surrender and connectedness to the divine has a distinguishable neurophysiological signature, supporting the broader neurotheological view that different religious and spiritual practices, even within the same tradition, engage partially distinct brain networks depending on their specific devotional goals and phenomenological content. |
| 65 | (Amihai & Kozhevnikov, 2014) | 2014 | To compare the neurophysiological (EEG, EKG) and cognitive correlates of meditative practices from the Theravada tradition (Shamatha and Vipassana) and the Vajrayana tradition (Deity and Rig-pa), in order to test whether meditation is better classified by relaxation versus arousal responses rather than by the focused versus distributed attention framework. | Study 1: 19 (10 Theravada, 9 Vajrayana); Study 2: 50 (12 Kasina, 14 Vipassana, 9 Rig-pa, 15 Deity, with some overlap between Study 1 and Study 2 participants) | Study 1: Theravada 20% (2 of 10), Vajrayana 11% (1 of 9). Study 2: Kasina group 17% (2 of 12), Vipassana group 29% (4 of 14), Rig-pa group 11% (1 of 9), Deity group 33% (5 of 15) | Theravada (Study 1): 41.4 years; Vajrayana (Study 1): 47.5 years; Kasina group: 43.2 years; Vipassana group: 43.2 years; Rig-pa group: 47.5 years; Deity group: 42 years |  | EEG | Buddhism | Theravada Buddhist meditation practice | Shamatha (Kasina), Vipassana, Deity visualization (Kyerim), and Rig-pa meditation | Frontal, central, and posterior scalp regions (POz, Pz, Fz, C3, C4, F3, F4, P3, P4); frontal and fronto-central coherence particularly implicated | Theravada meditations (Shamatha and Vipassana) produced increased parasympathetic activation (increased high-frequency power, decreased low-to-high frequency ratio, and decreased alpha power), consistent with a relaxation response. Vajrayana meditations (Deity and Rig-pa) produced decreased high-frequency power, indicating sympathetic activation and an arousal response, though without significant changes in the low-to-high frequency ratio. Beta power decreased in both traditions but was more pronounced during Vajrayana practices. Gamma power increased during Vipassana but decreased during both Vajrayana practices. Only Vajrayana meditators (Deity and Rig-pa) showed a significant, immediate improvement in cognitive task performance (mental rotation and visual memory efficiency) after meditation, indicating enhanced phasic alertness; Theravada practitioners showed no such improvement. | Theravada and Vajrayana meditative traditions rely on distinct neurophysiological mechanisms: Theravada practices (Shamatha and Vipassana) are associated with a relaxation response marked by parasympathetic activation, while Vajrayana practices (Deity and Rig-pa) are associated with an arousal response marked by sympathetic activation and enhanced phasic alertness. These findings did not support the traditional focused attention versus open monitoring classification, since practices within the same tradition (regardless of attentional style) showed highly similar patterns, while practices across traditions differed substantially. |
| 66 | (Doufesh et al., 2014) | 2014 | To evaluate the possible correlations between the spectral power of the alpha (α) band frequency of EEG and HF or LF bands of HRV during salat (Muslim prayer) and to elucidate the physiologic mechanisms between salat and the CNS (Central Nervous System) and ANS (Autonomic Nervous System). | 30 | 0 |  |  | EEG; ECG | Islam | Prayer |  | Occipital and parietal regions of both brain hemispheres (electrodes O1, O2, P3, P4 showed the most significant changes) | During salat, alpha relative power (RPα) significantly increased in the occipital and parietal regions of the brain Parasympathetic activity increased Sympathetic activity decreased Significant positive correlations were found between RPα in occipital/parietal regions and nuHF (parasympathetic index) Significant negative correlations were found between RPα and nuLF/LF/HF (sympathetic indices) | Increased alpha (α) wave activity in the occipital and parietal regions during salat These changes are associated with: Increased relaxation Reduced tension Sustained focus Balanced condition of mind and body Positive changes in brain function and human well-being |
| 67 | (Kalamangalam, 2014) | 2014 | To identify structural brain correlates of exceptional long-term auditory-verbal memory training in Hindu Vedic priests, testing the hypothesis that cortical thickness would be altered in regions involved in verbal semantic and prosodic encoding, storage, and retrieval, given the priests' extraordinary oral memorization of Vedic scripture. | 22 (11 Vedic priests, 11 matched controls) | 0 | 33.3 (priests); 32.6 (controls) |  | MRI | Hinduism | Vedic priesthood, involving oral recitation and memorization of Vedic scriptural hymns in classical Sanskrit, learned through 8 to 12 years of full-time apprenticeship (starting between ages 5 and 18) and sustained through active priestly practice (median 11 years since training completion) | Lifelong Vedic scriptural recitation and oral transmission, involving memorization of over 10,000 stanzas set to characteristic cadence and melodic contour, recalled spontaneously during ongoing spiritual and ritual practice | Left orbitofrontal cortex (medial, ventral, and polar regions, including gyrus rectus and medial orbital gyrus; Brodmann areas 10, 11, 14) and right inferior and middle temporal gyrus (straddling the inferior temporal sulcus; Brodmann areas 20, 21) | Vedic priests showed significantly greater cortical thickness than matched controls in two discrete clusters: the left orbitofrontal cortex and the right inferior and middle temporal gyrus. There were no significant group differences in total cortical gray matter volume or in overall mean cortical thickness of either hemisphere, and no regions of significant cortical thinning were found in priests. No hippocampal volume differences were observed, consistent with the semantic (rather than episodic or spatial) nature of the memorized material. | Sustained, lifelong religious practice involving intensive oral memorization of scripture is associated with focal, training-related cortical thickening in brain regions supporting verbal semantic encoding and retrieval (left prefrontal cortex) and prosodic or tonal auditory processing (right temporal cortex), rather than global structural change or hippocampal adaptation. These findings suggest that prolonged religious-textual memorization practice can produce localized, expertise-driven cortical plasticity, supporting the broader view that intensive spiritual and devotional training leaves durable, function-specific structural signatures in the human brain. |
| 68 | (Taha Alshaikhli et al., 2014) | 2014 | To study and compare the effects on EEG and ECG signals while listening to Quran recitation versus listening to hard music, in order to determine which condition produces greater relaxation. | 3 |  |  |  | EEG | Islam | Listening to Quran recitation |  | Frontal and central regions (F3, F4, C3, C4) | Subjects showed higher gamma wave activity while listening to Quran recitation compared to music. Subject one's emotional trajectory stayed in the "happy" quadrant with high arousal during Quran recitation, but shifted to a "sad" quadrant during music. Subject two, who was sleepy, showed reduced sleepiness during Quran recitation compared to music. ECG signals were smoother during Quran recitation, suggesting a calmer physiological state, although there were no major differences in overall heart rate (BPM) between conditions. | The EEG results indicated that subjects were more relaxed while listening to Quran recitation than while listening to hard music. The ECG results showed smoother signals during Quran recitation, which the authors interpreted as reflecting greater calmness. The authors recommend that future studies use larger samples and explore the correlation between EEG and ECG signals in more depth. |
| 69 | (Taren et al., 2014) | 2014 | To examine whether self-reported perceived stress is associated with amygdala–subgenual anterior cingulate cortex (sgACC) resting-state functional connectivity (rsFC), and to determine whether a three-day mindfulness meditation training programme differentially reduces stress-related amygdala–sgACC rsFC relative to a matched relaxation control intervention. | 165 | 45 | 40 | 7.16 | fMRI | Mixed |  | Mindfulness Meditation | Subgenual Anterior Cingulate Cortex (sgACC), amygdala | Elevated perceived stress was significantly associated with greater bilateral amygdala–sgACC rsFC in community adults. A three-day mindfulness-based training programme significantly reduced right amygdala–sgACC rsFC compared to the relaxation control. Pre-to-post reductions in right amygdala–sgACC rsFC were inversely associated with hair cortisone levels at four-month follow-up (r = −0.39, p = 0.049), suggesting attenuated cumulative hypothalamic-pituitary-adrenal axis activation. | Mindfulness meditation training produced neuroplastic decoupling of the amygdala–sgACC circuit, a pathway implicated in stress reactivity and affective regulation. Reduced functional connectivity between these regions corresponded with measurable reductions in neuroendocrine stress markers at extended follow-up. |
| 70 | (Thomas et al., 2014) | 2014 | To use eLORETA source analysis to compare cortical source activity between intermediate and advanced Satyananda Yoga meditators during a body steadiness meditation, a mantra meditation, and a non meditation mental calculation condition, testing whether meditation proficiency is marked by a developmental shift from low frequency (theta, alpha) to high frequency (gamma) EEG activity, consistent with traditional stages of Yoga meditative absorption. | 12 (6 Satyananda Yoga Teachers, 6 Satyananda Yoga Students) | 50 | 54 (teacher group); 42 (student group) | 6.5 (teacher group); 8.0 (student group) | EEG | Hinduism |  | Yoga | Right precentral gyrus, right primary somatosensory cortex, right superior frontal gyrus, right parietal and occipital lobes (alpha1 band, greater in students); right insula, right inferior frontal gyrus, right anterior temporal lobe including fusiform and middle temporal gyri (gamma band, greater in teachers); core right lateralized networks that expanded to homologous left hemisphere regions with increased meditation depth and practice | Intermediate meditators (students) showed significantly greater low frequency (theta, alpha1, alpha2) cortical source activity than advanced meditators (teachers), concentrated in right somatosensory, motor, premotor, parietal, and occipital regions, across both meditation conditions and, to a lesser extent, the non meditation calculation condition. Advanced meditators (teachers) showed significantly greater high frequency (beta, gamma) activity than students, concentrated in the right insula, inferior frontal gyrus, and anterior temporal lobe, with the number of significant voxels roughly doubling during meditation compared to the non meditation condition. Both alpha1 and gamma differences were predominantly right lateralized, with practice related expansion into homologous left hemisphere regions during meditation. Contrary to prediction, midline default mode network hubs were not the principal locus of proficiency related differences. | Sustained Yoga meditation practice, particularly repetitive mantra recitation and body steadiness practice within the Satyananda tradition, is associated with a progressive, experience dependent shift in right lateralized cortical network activity: intermediate practitioners show relatively greater low frequency activity consistent with early stage sensory withdrawal (pratyahara), while decades long practitioners show markedly greater high frequency gamma activity consistent with later stage concentrative absorption (dharana and dhyana). |
| 71 | (Dor-Ziderman et al., 2013) | 2013 | To map the neural correlates of three hierarchical states of Buddhist selfless awareness;Narrative Self (NS), Minimal Self (MS), and Selfless (SL) state;using magnetoencephalography within a neurophenomenological framework, specifically examining frequency-band-specific contributions to self-representation and ego dissolution. | 12 | 25 | 45.2 | 11.3 | MEG | Buddhism | Meditation |  | Medial Prefrontal Cortex (mPFC), Anterior Cingulate Cortex (ACC) / Subgenual ACC, Anterior Cingulate Cortex (ACC) / Subgenual ACC, Thalamus (bilateral), Right & Left Inferior Parietal Lobule (IPL), Posterior Cingulate Cortex (PCC) / Precuneus, Left Insula, Left Dorsomedial Thalamus, Paracentral Lobule | Transitioning from NS to MS was associated with significant decreases in high-gamma (60–80 Hz) oscillatory power predominantly in frontal and medial prefrontal cortex regions. Transitioning from MS to SL involved significant decreases in beta-band (13–25 Hz) power in the ventral mPFC, medial posterior regions, bilateral inferior parietal lobule, thalamus, and posterior cingulate cortex/precuneus. The most experienced meditators who reported a Lack-of-Ownership SL experience showed additional beta suppression specifically in the right inferior parietal lobule and left dorsomedial thalamus. | Buddhist meditation-induced progressive selflessness is neurophysiologically characterized by hierarchical, frequency-specific oscillatory suppression: high-gamma reduction in mPFC during narrative disengagement, and beta-band suppression in a distributed self-model network during full ego dissolution. The association between beta attenuation in the inferior parietal lobule and reported loss of bodily ownership constitutes a novel electrophysiological signature of advanced contemplative experience. |
| 72 | (Garrison et al., 2013) | 2013 | To investigate the subjective experiential states corresponding to real-time posterior cingulate cortex (PCC) activation and deactivation in adept meditators during mindfulness practice, using fMRI neurofeedback. | 10 | 30 | 49.2 | 12.5 | fMRI | Mixed | Meditation |  | Posterior Cingulate Cortex (PCC) | States of undistracted awareness (concentration, sensory observation) and effortless doing (non-efforting, contentment) corresponded with PCC deactivation. Distracted awareness (distraction, interpretive thinking) and controlling effort (efforting, discontentment) corresponded with PCC activation. Effortful attempts to meditate produced PCC activation, whereas effortless natural awareness produced PCC deactivation. Mind wandering did not invariably activate the PCC; the quality of engagement (caught up in versus observing thoughts) determined the direction of PCC activity. | The posterior cingulate cortex serves as a real-time index of metacognitive engagement quality during meditation. Effortless, undistracted awareness; regardless of phenomenal content; produces PCC deactivation, while effortful control and attentional capture activate this default mode network hub. This dissociation refines understanding of how contemplative expertise modulates self-referential neural dynamics. |
| 73 | (Doufesh et al., 2012) | 2012 | To investigate the electrophysiological proposition of physiological relaxation during Muslim prayer (Salat) by measuring alpha brain activity across frontal, central, parietal, and occipital scalp regions during the Dhuha prayer and a matched acted condition. | 9 | 33 |  |  | EEG | Islam | Prayer |  | Parietal region (P3, P4) Occipital region (O1, O2) Frontal region (F3, F4) Central region (C3, C4) | No significant differences in mean alpha relative power were identified between the actual Dhuha prayer and the acted condition (physical movements without recitation) across eight electrode positions. Higher alpha amplitude was observed during the prostration posture in both conditions at parietal and occipital regions relative to resting state. These findings indicate that the physical prostration position itself, independent of recitation, is sufficient to generate increased alpha activity. | The prostration posture inherent to Salat produces reliable parieto-occipital alpha augmentation, indicative of cortical deactivation and parasympathetic nervous system activation. The absence of differential alpha enhancement for actual versus acted prayer suggests that the biomechanical component of prayer posture, rather than concurrent recitation, principally drives EEG relaxation responses. |
| 74 | (Kozasa et al., 2012) | 2012 | To evaluate whether regular meditation practitioners demonstrate greater neural efficiency during a cognitive conflict task (fMRI-adapted Stroop Word-Colour Task);defined as equivalent behavioural performance with reduced neural activation;relative to non-meditators. | 39 | 56 | 45.13 | 9.3 | fMRI | Mixed |  | Meditation | Right medial frontal gyrus (BA 9/10), right middle temporal gyrus (BA 21), right lentiform nucleus, right precentral gyrus (BA 4/6), right postcentral gyrus (BA 2), and right middle frontal gyrus (BA 6). | Non-meditators exhibited significantly greater BOLD activation than meditators in the right medial frontal gyrus, right middle temporal gyrus, right lentiform nucleus, right precentral gyrus, and right postcentral gyrus during incongruent Stroop conditions, despite equivalent behavioral performance. No regions were more activated in meditators than non-meditators. | Meditation training is associated with increased neural efficiency in attentional and motor control circuits. Regular meditators accomplish equivalent cognitive performance with significantly reduced neural resource expenditure in frontal, temporal, and subcortical regions, consistent with expertise-driven economy of neural processing. |
| 75 | (Peres et al., 2012) | 2012 | To investigate whether dissociative trance states during psychographic mediumship (automatic writing) are associated with specific alterations in regional cerebral blood flow compared to non-trance writing, and to examine the relationship between neural activity and the linguistic complexity of produced content. | 10 | 60 | 48.3 | 28.29 | SPECT | Spiritism |  | Psychography (Automatic Writing), mediumship | Left culmen, left hippocampus, left inferior occipital gyrus, left anterior cingulate cortex, right superior temporal gyrus, right precentral gyrus. | Experienced mediums exhibited decreased rCBF in the left culmen, left hippocampus, left inferior occipital gyrus, left anterior cingulate cortex, right superior temporal gyrus, and right precentral gyrus during psychography relative to control writing. Less experienced mediums showed increased activation in these same regions during psychography. Psychographically-produced texts exhibited greater complexity than control writing, and increases in textual complexity were inversely associated with rCBF. | Experienced mediums display hypoperfusion in regions subserving cognitive monitoring, attention, memory encoding, language production, and voluntary motor control during dissociative trance states. The inverse relationship between complexity of output and neural activation in experienced practitioners may reflect automatized or non-consciously mediated information processing, analogous to expertise-driven neural efficiency documented in other cognitive domains. |
| 76 | (Zulkurnaini et al., 2012) | 2012 | To investigate and compare the effects of listening to Al-Quran recitation and classical music on the human brain's alpha band using EEG, focusing on brainwave balancing (left-right correlation) before, during, and after listening. | 28 |  |  |  | EEG | Islam | Listening to Surah Yasin recitation of the Al-Quran (compared against listening to Pachelbel's Canon D major as a secular control condition) |  | Left and right hemispheres (general, not region-specific; measured via left-right brainwave correlation and alpha amplitude) | Listening to Al-Quran produced a 12.67% increase in left-right brainwave correlation, compared to a 9.96% increase for classical music, indicating Al-Quran had a greater effect on alpha band brainwave balancing. Mean alpha values decreased from before to after listening in both conditions, but individual cases (e.g., sample 21) showed alpha increases up to 24% during Al-Quran listening. Overall, correlation values remained high for both conditions (above 0.82), with classical music achieving the single highest correlation value (0.927) and Al-Quran producing the greater percentage improvement. | Listening to Al-Quran recitation increases alpha band brainwave balancing more than listening to classical music, suggesting it can produce a more relaxing and alert state. The findings support the idea that Al-Quran recitation may serve as a method to promote relaxation, release tension, and reduce stress, consistent with the concept of religious psychotherapy providing lasting physiological benefits. |
| 77 | (Brewer et al., 2011) | 2011 | To investigate brain activity in experienced mindfulness meditators versus controls during different meditation types (Concentration, Loving-Kindness, Choiceless Awareness); specifically to test whether the default mode network (DMN) shows reduced activation and altered connectivity in meditators. | 24 |  |  |  | fMRI | Mixed |  | Mindfulness Meditation | Default Mode Network (medial prefrontal cortex, posterior cingulate cortex/precuneus); dorsal anterior cingulate cortex; dorsolateral prefrontal cortex; insula; amygdala; hippocampus; temporal gyri; angular gyrus | Experienced meditators showed: (1) relatively reduced activation in DMN nodes (medial prefrontal cortex and posterior cingulate cortex) during all meditation types; (2) stronger functional connectivity between posterior cingulate cortex and dorsal anterior cingulate/dorsolateral prefrontal cortices (self-monitoring and cognitive control regions) at both baseline and during meditation; (3) significantly less self-reported mind-wandering during meditation. | Reduced DMN activation (deactivation in mPFC and PCC); increased functional connectivity between PCC and cognitive control regions (dACC, dlPFC); altered connectivity patterns suggesting more present-centered default mode even at rest; potential coupling of DMN with self-monitoring regions that may dampen mind-wandering |
| 78 | (Hölzel et al., 2011) | 2011 | To investigate pre-to-post changes in regional brain grey matter concentration attributable to participation in an eight-week Mindfulness-Based Stress Reduction (MBSR) programme, using voxel-based morphometry, relative to a waitlist control group. | 33 | 49 | 38.52 | 7.2 | MRI | Buddhism | Meditation |  | Left hippocampus; posterior cingulate cortex (PCC); left temporo-parietal junction (TPJ); cerebellum (vermis/brainstem and lateral lobule VIII/X). | Eight weeks of MBSR training produced significant increases in grey matter density in the left hippocampus, posterior cingulate cortex, left temporo-parietal junction, and cerebellum. These structural changes were not observed in the waitlist control group. Increases in hippocampal grey matter density are particularly notable given that major depressive disorder and post-traumatic stress disorder are associated with hippocampal volume reduction. | Mindfulness practice induces measurable structural neuroplasticity in regions subserving learning and memory (hippocampus), self-referential processing (posterior cingulate), perspective-taking and empathy (temporo-parietal junction), and procedural learning (cerebellum). These morphological changes may represent a biological basis for clinically documented benefits of MBSR in affective and anxiety disorders. |
| 79 | (Kalyani et al., 2011) | 2011 | To examine the neurohemodynamic correlates of audible OM chanting using fMRI, specifically testing the hypothesis that OM chanting produces limbic system deactivation via vagal nerve stimulation mediated by vibration sensation in the auricular region. | 12 | 25 | 28 | 6 | fMRI | Hinduism | Chanting |  | Amygdala, Hippocampus, Parahippocampal gyrus , Anterior cingulate gyrus/cortex , Orbitofrontal cortex , Insula, Thalamus | Audible OM chanting produced significant bilateral deactivation in the orbitofrontal cortex, anterior cingulate gyrus, parahippocampal gyri, thalami, hippocampi, and right amygdala. No regions showed significant activation during OM chanting. The control condition ('ssss' pronunciation) produced neither activation nor deactivation in these limbic structures. | OM chanting selectively deactivates limbic and paralimbic structures implicated in emotional reactivity, memory consolidation, and stress response. This deactivation pattern is neurobiologically analogous to that produced by vagus nerve stimulation;a clinically established treatment for depression and epilepsy; suggesting that OM chanting may exert therapeutic effects via auricular vagal afferent activation. |
| 80 | (Schjoedt et al., 2011) | 2011 | To examine how perceived charismatic authority modulates BOLD responses in Christian versus secular participants during intercessory prayer reception, specifically testing whether attribution of healing power to a speaker downregulates prefrontal executive control networks. | 36 | 60 | 24.7 | 3.92 | fMRI | Christianity, secular | Prayer |  | Medial prefrontal cortex; dorsolateral prefrontal cortex (bilateral); anterior cingulate cortex; temporoparietal junction (bilateral); inferior temporal cortex; temporopolar/ventrolateral orbitofrontal region; precuneus; amygdaloid region; insula; cerebellum; medulla. | Christian participants exhibited significant deactivation of the frontal executive network; encompassing bilateral medial and dorsolateral prefrontal cortex and anterior cingulate cortex; when listening to speakers believed to possess charismatic healing abilities. Magnitude of neural deactivation was inversely correlated with post-scan charisma ratings and subjective experience of divine presence. Secular participants showed no equivalent modulation. Findings parallel neural mechanisms reported in hypnotic susceptibility. | Selective deactivation of prefrontal executive and social cognitive networks in Christian participants in response to perceived charismatic authority, suggesting voluntary suspension of critical evaluation. Increased activation was observed in response to non-Christian speakers, indicating a belief-congruent modulation of executive function. |
| 81 | (Zeidan et al., 2011) | 2011 | To investigate the neural mechanisms by which mindfulness meditation modulates pain perception in healthy adults, using arterial spin labelling functional MRI to quantify changes in regional cerebral blood flow during noxious stimulation before and after a four-day meditation training programme. | 15 | 60 | 26 |  | fMRI | Mixed |  | Mindfulness Meditation | Primary somatosensory cortex (SI) Anterior cingulate cortex (ACC) Anterior insula (AI) Orbitofrontal cortex (OFC) Thalamus Posterior insula/secondary somatosensory cortex (SII) Medial prefrontal cortex (mPFC) Posterior cingulate cortex Ventral striatum Amygdala | Four days of mindfulness meditation training reduced pain unpleasantness by 57% and pain intensity by 40%. Meditation reduced pain-related activation in the contralateral primary somatosensory cortex (SI). Pain intensity reductions correlated with increased anterior cingulate cortex and anterior insula activation. Pain unpleasantness reductions correlated with orbitofrontal cortex activation and thalamic deactivation. | Mindfulness meditation modulates pain processing through multiple dissociable neural mechanisms: sensory-discriminative processing is attenuated via SI deactivation; cognitive reappraisal of pain is engaged via anterior cingulate and insular activation; contextual evaluation is restructured via orbitofrontal cortex recruitment; and thalamic gating of ascending nociceptive signals is enhanced. These data indicate that mindfulness engages active neural mechanisms rather than passive disengagement from pain. |
| 82 | (Bærentsen et al., 2010) | 2010 | To investigate meditation with fMRI in order to identify and characterize both the "neural switch" mechanism used in the voluntary shift from normal consciousness to meditation, and the "threshold regulation mechanism" sustaining the meditative state. | 31 (onset of meditation subset); 21 (continuous meditation subset) | 45% in the onset subset (14 females, 17 males); 52% in the continuous meditation subset (11 females, 10 males) | 45 years (onset subset); 44.5 years (continuous meditation subset) |  | fMRI | Buddhism |  | Meditation: involving respiration counting (Z group) or imaginative mantras tied to the respiratory cycle (M and T groups), studied both during the onset of meditation (45 second on/off blocks) and during 14.5 minutes of sustained meditation | Bilateral putamen, supplementary motor cortex, precentral gyrus, inferior parietal lobe, and right insula (activations during onset); right precuneus, posterior cingulate, and parieto-temporal junction (deactivations during onset); head of the caudate nucleus (activation during sustained meditation); widespread white matter areas in the right hemisphere including frontal, parietal, temporal, and occipital regions, insula, claustrum, and putamen (deactivations during sustained meditation); and a distributed component (identified through ICA) spanning prefrontal cortex, anterior and posterior cingulate, thalamus, basal ganglia, insula, and parahippocampal areas | During meditation onset, activations were found bilaterally in the putamen and supplementary motor cortex, while deactivations occurred predominantly in the right hemisphere, precuneus, posterior cingulum, and parieto-temporal area. During sustained meditation, SPM analysis revealed activation in the head of the caudate nucleus, along with extensive deactivations in white matter, mainly in the posterior occipito-parieto-temporal area and frontal lobes of the right hemisphere. ICA identified 38 components, twelve of which corresponded to known baseline resting state networks; one component, closely overlapping with the SPM-identified activated area, extended into frontal, temporal, parietal, and limbic areas and appeared related to attention regulation networks. Correlational analysis among components revealed a complex and variable pattern of relationships rather than a single stable mechanism. | The patterns of activation and deactivation during the shift from resting to meditation support the notion that meditation is a distinct state of mind, different from both resting baseline and externally directed cognitive tasks. These findings are consistent with the hypothesis of a "neural switch" involving a cortico-striato-thalamo-cortical loop that redirects attention from external matters to the subject's own state of mind and body. During continuous meditation, attention regulation appears to be mediated by functional networks distributed across dorsolateral and ventrolateral prefrontal areas, the cingulate gyrus, inferior parietal lobe, temporal and occipital lobes, thalamus, and cerebellum. |
| 83 | (Cahn et al., 2010) | 2010 | To characterise state effects of Vipassana meditation on spontaneous EEG activity in long-term practitioners relative to a mind-wandering control condition, examining spectral power across delta, theta, alpha, beta, and gamma frequency bands. | 16 | 31 | 45.5 | 9.8 | EEG | Buddhism | Meditation | Vipassana Meditation | Parieto-occipital regions (O1, O2, P7, P8);showed increased gamma power Frontal regions (F3, F4, F7, F8);showed decreased delta power and relative theta increases Bilateral frontal areas;associated with attentional control and reduced cognitive elaboration | Significantly increased parieto-occipital gamma (35–45 Hz) power during meditation constituted the most robust finding, with effects strongest in practitioners with ten or more years of daily practice. Frontal delta (1–4 Hz) power decreased during meditation, particularly in non-drowsy participants. A relative increase in frontal theta (4–8 Hz) power at the Fz electrode was observed. Alpha and beta bands showed no significant state effects. | Vipassana meditation produces a characteristic electrophysiological profile of parieto-occipital gamma augmentation and frontal delta suppression, reflecting a state of heightened sensory awareness combined with reduced attentional engagement with distracting cognitive content. Greater baseline occipital gamma power in long-term practitioners constitutes a trait-level neurophysiological marker of contemplative expertise. |
| 84 | (Manna et al., 2010) | 2010 | To characterize and contrast the neural correlates of focused attention (FA) and open monitoring (OM) meditation using fMRI in experienced Theravada Buddhist monks and novice practitioners, examining whether these practices enhance or reduce activations in frontal executive areas. | 16 | 0 | 34.95 | 6.98 | fMRI; EEG; ECG | Buddhism | Meditation | Focused Attention (Samatha) And Open Monitoring (Vipassana) Meditation | Left fronto-parietal areas, insula, dorsal anterior cingulate cortex, dorsolateral prefrontal cortex, medial anterior prefrontal cortex, precuneus, superior parietal lobule, superior temporal gyrus, middle frontal gyrus, and inferior frontal gyrus | Expert meditators demonstrated significant state-dependent self-regulation of left-hemisphere fronto-parietal and insular areas. FA meditation enhanced right medial frontal activation while reducing left lateral prefrontal engagement; OM meditation increased left medial frontal activation. The anterior cingulate and dorsolateral prefrontal cortices exhibited antagonistic roles in executive attention control. Meditation-related functional reorganization of prefrontal and insular activity was interpreted as evidence of neuroplasticity. | FA and OM meditation produce dissociable patterns of prefrontal and insular self-regulation reflecting distinct attentional strategies. Long-term practice induces functional reorganization of executive control networks, with the anterior cingulate and dorsolateral prefrontal cortex serving opposing regulatory roles across meditation styles. |
| 85 | (Beauregard et al., 2009) | 2009 | To measure brain activity during a meditative state in near-death experiencers (NDErs) when they mentally re-engaged with the "being of light" from their near-death experience, using combined fMRI and EEG methodologies. | 15 | 40 | 47 | 12 | fMRI; EEG | Theism | Meditation | Near-Death Experience Meditation | rightbrainstem,rightlateralorbitofrontal cortex, right medial prefrontal cortex, right superior parietal lobule, left superior occipital gyrus, left anterior temporal pole, left inferior temporal gyrus, left anterior insula, left parahippocampal gyrus and left substantia nigra | fMRI data revealed significant activation in the right brainstem, right lateral orbitofrontal cortex, right medial prefrontal cortex, right superior parietal lobule, left superior occipital gyrus, left anterior temporal pole, left inferior temporal gyrus, left anterior insula, left parahippocampal gyrus, and left substantia nigra during meditation. EEG data showed significantly greater theta power at widespread scalp sites, along with increased alpha power at frontal and temporal sites and increased gamma power at right prefrontal and temporal electrodes during the meditative condition. | Meditative re-engagement with near-death imagery produces a distinctive neurobiological profile encompassing activation of networks subserving positive affect and reward (orbitofrontal cortex), self-referential processing (medial prefrontal cortex), visual imagery (occipital cortex), autobiographical memory (parahippocampal gyrus), and spiritual experience-related regulation (insula). Concurrent multiband EEG augmentation reflects a state of heightened, integrated neural processing. |
| 86 | (Ge et al., 2009) | 2009 | To test whether Christian belief and practice produce a semantic trait summary representation of the religious leader (Jesus) in believers, resulting in reduced episodic memory retrieval during trait judgment, as evidenced by behavioural priming and functional connectivity analyses. | 60 | 50 | 23.45 | 4.24 | fMRI | Christianity | Prayer | Prayer And Bible Reading | Medial Prefrontal Cortex (MPFC) - specifically: VMPFC DMPFC  Posterior Parietal Cortex (PPC)/Precuneus | Behavioral data indicated that episodic recall facilitated Jesus trait judgments in non-religious participants but not in Christians, suggesting that Christian belief consolidates a semantic trait summary that bypasses episodic retrieval. Neuroimaging revealed that non-religious participants showed enhanced functional connectivity between medial prefrontal cortex (MPFC) and posterior parietal cortex/precuneus during both government and religious leader trait judgment, whereas Christians exhibited this enhanced connectivity only for government leaders; not for Jesus. MPFC-PPC/precuneus connectivity strength during Jesus judgment correlated negatively with degree of religious conviction. Linear SVM classification achieved 71.4% accuracy in distinguishing Christians from non-religious participants based on connectivity patterns. | Christian religious belief and practice fundamentally alter the neurocognitive architecture of religious leader representation, replacing episodic-retrieval-dependent processing (MPFC-PPC connectivity) with direct semantic access analogous to self-processing. The strength of this neural modification scales with depth of religious conviction, indicating practice-dependent neural reorganization. |
| 87 | (Huang & Lo, 2009) | 2009 | To explore central nervous system dynamics under Zen meditation using a nonlinear complexity index (average complexity index, denoted delta) combined with spectral power analysis, comparing experienced Zen meditation practitioners with matched controls. | 46 (23 Zen meditation practitioners, 23 controls) | 32.6% overall (7 of 23 in the meditation group, 8 of 23 in the control group) | 31.5 (meditation group), 29.5 (control group) | 5.7 (meditation group), 3.9 (control group) | EEG | Buddhism | Meditation |  | Frontal region, occipital region, temporal region, central and parietal midline regions (Fz, FCz, Cz, CPz, Pz, Oz) | Meditators showed a significant increase in frontal alpha-1 power and occipital beta power compared to controls, along with an overall increase in the average complexity index, particularly at occipital, temporal, and anterior sites. Controls instead showed an increase in theta power during rest. Complexity index correlated positively with relative beta power (r = 0.84) and alpha power (r = 0.21), and negatively with delta and theta power. The distinction between meditators and controls became more pronounced during the last five minutes of the session, with meditators exhibiting high-complexity, beta-dominated EEG activity concentrated over occipital and parietal regions in later stages. | Long-term Zen Buddhist meditation training induces measurable changes in electro-cortical activity, characterized by increased EEG complexity and beta power over the occipital cortex, alongside frontal alpha-1 increases. These findings support the idea that Zen meditation shifts practitioners from a normal relaxed state toward a state of heightened, inner mindful attention, and the emergence of beta rhythm may reflect deeper meditative states such as samadhi. The authors also suggest a possible role for serotonergic activity in producing this high-frequency, high-complexity EEG pattern, though this requires further neurochemical verification. |
| 88 | (Lutz et al., 2009) | 2009 | To examine how meditation expertise modulates the coupling between cardiac function and BOLD signal in the right middle insula during compassion meditation, comparing novice practitioners and long-term Buddhist meditators. | 23 |  | 39 | 9.4 | fMRI | Buddhism | Meditation |  | right middle insula, left middle/posterior insula, dorsal anterior cingulate cortex, somatosensory cortices and the right inferior parietal lobule | BOLD signal in the right middle insula showed significant positive association with heart rate across states and groups. This neurovisceral coupling was significantly stronger in the left middle/posterior insula among expert meditators relative to novices. Dorsal anterior cingulate cortex demonstrated a positive state-dependent coupling with heart rate during compassion meditation that was more pronounced in experts, particularly in somatosensory cortices and right inferior parietal lobule. | Long-term compassion meditation expertise enhances the integration of cardiac interoceptive signals with somatosensory and insular brain networks, reflecting a neuroplastic refinement of neurovisceral coherence. Expertise-dependent modulation of the anterior cingulate and somatosensory cortex indicates that advanced practice strengthens bodily resonance with others' emotional states. |
| 89 | (Schjøedt et al., 2009) | 2009 | To characterise the differential neural correlates of formalized versus improvised prayer in Danish Christians using fMRI, testing whether personal, intersubjective prayer engages social-cognitive networks comparably to direct interpersonal communication. | 20 | 70 | 25.4 | 2.75 | fMRI | Christianity | Prayer |  | Temporoparietal junction (TPJ), Temporopolar region, Medial prefrontal cortex (MPFC), Precuneus, Dorsolateral prefrontal cortex, Caudate nucleus | Improvised personal prayer activated regions constituting the social cognition (Theory of Mind) network, including the temporoparietal junction, temporopolar region, medial prefrontal cortex, and precuneus. Formalized recitation (Lord's Prayer) recruited dorsolateral prefrontal cortex, parietal cortex, and cerebellum; regions associated with verbal rehearsal and episodic retrieval. No significant main effects of religious speech acts relative to secular controls were identified in whole-brain analysis. | Improvised prayer to God recruits intersubjective social-cognitive circuitry equivalent to real interpersonal interaction, indicating that sincere prayerful address is neurologically processed as authentic dialogic communication with a perceived agent. Formalized prayer engages executive and memory-retrieval networks consistent with learned procedural recitation. |
| 90 | (Pagnoni et al., 2008) | 2008 | To investigate the neural correlates of conceptual processing during Zen meditation in regular practitioners and matched controls, examining whether meditation training modulates default network responses to semantic stimuli. | 24 | 21 | 36.3 | 36.3 | fMRI | Buddhism | Meditation |  | Left angular gyrus, left superior frontal gyrus, left middle cingulate cortex, left posterior cingulate cortex, left rostral anterior cingulate cortex, left inferior temporal gyrus | Zen practitioners demonstrated significantly reduced duration of BOLD response to semantic processing in default network regions relative to controls, with faster return to baseline. In the left angular gyrus and related regions, the BOLD signal dropped below baseline levels following conceptual stimulation, indicating active suppression of conceptual elaboration. No significant between-group differences in behavioral performance were observed. | Zen meditation is associated with expedited neural disengagement from conceptual and self-referential processing in default mode network nodes. The below-baseline BOLD response in experienced practitioners suggests active inhibitory modulation of semantic elaboration consistent with the Zen principle of non-conceptual awareness. |
| 91 | (Han et al., 2008) | 2008 | To determine whether Christian religious belief is associated with distinct neural correlates of self-referential processing, testing the hypotheses that Christian belief attenuates ventral medial prefrontal cortex (VMPFC) activity during self-evaluation while enhancing dorsal medial prefrontal cortex (DMPFC) engagement reflecting a God's-perspective orientation. | 28 | 50 | 23.69 | 4.44 | fMRI | Christianity | Prayer | Self-Transcendence And God-Perspective Reflection | Ventral medial prefrontal cortex (VMPFC, BA 10); Dorsal medial prefrontal cortex (DMPFC, BA 9/32); Anterior cingulate cortex (ACC); Posterior visual cortex/calcarine sulcus (BA 18); Right inferior parietal cortex (BA 39/40); Precuneus (BA 31); Left inferior frontal gyrus (BA 45/47); Superior medial frontal cortex (BA 9); Left middle temporal gyrus (BA 21/37); Cerebellum | Self-referential processing in non-religious participants was associated with increased VMPFC activity, consistent with stimulus self-relatedness coding. Christian participants instead demonstrated elevated DMPFC activity, reflecting evaluative self-referential processing from a transcendent perspective. Christians exhibited no VMPFC differentiation between self and others, indicating attenuation of self-relevance coding. DMPFC activation in Christians was positively correlated with the rated importance of divine judgment in personality evaluation. Deactivation of right inferior parietal cortex and precuneus was also observed in Christians during self-judgment. | Religious belief restructures the neural architecture of self-referential processing, shifting activity from VMPFC (personal self-relevance) to DMPFC (evaluative, other-perspective-based processing). Concurrent parietal suppression suggests reduced engagement of episodic self-reflection in favor of a theologically-framed self-concept. |
| 92 | (Brefczynski-Lewis et al., 2007) | 2007 | to examine the neural basis of "one-pointed concentration" meditation to understand how long-term meditation practice affects attentional expertise. | 41 | 0 | 43.14 | 8.72 | fMRI | Buddhism | Meditation |  | Dorsolateral Prefrontal Cortex (DLPFC), Intraparietal Sulcus (IPS), Frontal Eye Fields (FEF), Anterior Insula, Anterior Cingulate Cortex (ACC), Posterior Cingulate/Precuneus, Amygdala, Thalamus, Basal Ganglia (Caudate, Putamen), Cerebellum, Visual Cortex (Occipital regions), Medial Frontal Gyrus, Inferior Frontal Gyrus (IFG) | 1) Expert meditators with ~19,000 hours of practice showed greater activation in attention-related brain regions than novices, but experts with ~44,000 hours showed less activation, suggesting increased processing efficiency with very long-term practice. 2) Differential response to distractions: When exposed to distracting sounds during meditation, expert meditators showed: a)Less activation in default-mode network regions (posterior cingulate, medial frontal gyrus/anterior cingulate) associated with mind-wandering and self-referential thoughts, b) Less activation in emotion-processing regions (amygdala), especially to negative sounds, c) More activation in regions related to response inhibition and attention (dorsal intraparietal sulcus, inferior frontal gyrus), 3) Time course differences: Highly experienced meditators showed brief, initial activation in attention regions that quickly returned to baseline, while less experienced meditators and novices showed sustained activation throughout the meditation block., 4) Negative correlation with practice hours: Many brain regions showed decreased activation with increasing hours of practice, suggesting neural efficiency. | 1)Highly experienced meditators (44,000+ hours) showed decreased activation in attention networks compared to moderately experienced meditators, suggesting the brain requires less effort to maintain focus, 2) Experts showed less activation in brain regions associated with mind-wandering and self-referential thoughts (posterior cingulate, medial frontal cortex), indicating less intrusive discursive thinking during meditation, 3) Experts showed decreased amygdala activation to emotional stimuli, particularly negative sounds, suggesting reduced emotional interference during meditation, 4) Greater activation in regions associated with inhibiting habitual responses (IFG, basal ganglia, subthalamic nuclei), suggesting improved ability to prevent automatic reactions to distractions, 5) Highly experienced practitioners showed rapid initial activation followed by quick return to baseline, suggesting they could enter a "settled" state of minimal effort more quickly. |
| 93 | (Farb et al., 2007) | 2007 | To examine the neural distinction between narrative self-focus (monitoring enduring traits) and experiential self-focus (attending to momentary experience) in novice participants and individuals trained in an eight-week mindfulness meditation programme. | 36 | 75 | 43.97 | 11.75 | fMRI | Buddhism |  | Mindfulness-Based Stress Reduction (MBSR) | mPFC, VMPFC, DMPFC, PCC, inferolateral PFC, middle temporal gyrus, hippocampus, inferior frontal gyrus, caudate, dorsolateral PFC, ventrolateral PFC, posterior parietal cortex, LPFC, insula, SII, IPL | In novices, experiential focus produced only focal reductions in medial prefrontal cortex (mPFC) relative to narrative focus. In trained participants, experiential focus elicited more extensive mPFC reductions alongside increased recruitment of a right-lateralised network encompassing lateral prefrontal cortex, insula, secondary somatosensory cortex, and inferior parietal lobule. Notably, the strong coupling between right insula and mPFC observed in novices (r = 0.61) was absent in trained participants, indicating a training-induced decoupling of interoceptive and narrative self-processing. | Mindfulness training produces a fundamental reorganization of self-referential processing, dissociating narrative autobiographical processing (mPFC) from present-moment somatic and interoceptive awareness (insula, secondary somatosensory cortex, inferior parietal lobule). The decoupling of insular-prefrontal connectivity suggests that meditation training enables transient self-experiences to be processed as phenomenal events rather than core self-defining constructs. |
| 94 | (Slagter et al., 2007) | 2007 | To investigate whether three months of intensive Vipassana meditation alters the allocation of limited attentional resources, as measured by attentional-blink performance and event-related EEG brain potentials. | 40 | 40 | 40.54 | 13.91 | EEG | Buddhism | Meditation | Vipassana Meditation | Parietal and frontal regions (P3b component sources); specifically electrode sites Pz, CP3, P3 (posterior parietal); implicates fronto-parietal-temporal network. | Three months of intensive meditation reduced the attentional blink and decreased brain-resource allocation to the first target (T1), reflected in a smaller T1-elicited P3b amplitude. Individuals demonstrating the greatest reduction in T1 P3b amplitude showed the largest corresponding decrease in attentional-blink magnitude, confirming that altered neural resource allocation mediates improved sequential target detection. | Intensive Vipassana meditation reduces the amplitude of the T1-evoked P3b component; an electrophysiological index of neural resource commitment; indicating that sustained practice facilitates more economical and flexible distribution of attentional resources across temporal targets. This neural efficiency enables more veridical perception of rapidly successive events. |
| 95 | (Beauregard & Paquette, 2006) | 2006 | To identify the neural correlates of mystical experience in Carmelite nuns by contrasting brain activation during recalled mystical union with God against a control condition of recalled intense interpersonal closeness with another person. | 15 | 100 | 49.93 | 11.27 | fMRI | Christianity | Mystical Experience |  | right medial orbitofrontal cortex, right middle temporal cortex, right inferior and superior parietal lobules, right caudate, left medial prefrontal cortex, left anterior cingulate cortex, left inferior parietal lobule, left insula, left caudate, left brainstem, and extra-striate visual cortex. | Mystical experience was associated with significant BOLD signal increases across a distributed network including the right medial orbitofrontal cortex, right middle temporal cortex, right inferior and superior parietal lobules, right caudate, left medial prefrontal cortex, left anterior cingulate cortex, left inferior parietal lobule, left insula, left caudate, left brainstem, and extrastriate visual cortex. These activation patterns were distinct from those elicited by the interpersonal closeness condition. | Mystical union engages neural systems subserving emotion (orbitofrontal cortex, caudate), self-representation (medial prefrontal cortex), body schema (parietal lobules), positive affect and reward (caudate), somatovisceral integration (insula), and visual imagery (extrastriate cortex). The convergence of these systems is consistent with the subjective phenomenology of unitive and blissful spiritual states. |
| 96 | (Newberg et al., 2006) | 2006 | To determine whether glossolalia (speaking in tongues) is associated with specific changes in regional cerebral activity using SPECT neuroimaging, specifically examining hypotheses regarding frontal lobe activity, superior parietal lobe activity, thalamic activity, and limbic structure function. | 5 | 100 | 45 | 5.24 | SPECT | Christianity | Glossolalia | Speaking In Tongues | Prefrontal cortices (bilateral dorsolateral prefrontal cortex, left dorsomedial cortex) Left caudate Left temporal pole Left superior parietal lobe Right amygdala Thalamus | Glossolalia was associated with significant decreases in rCBF in bilateral prefrontal cortices, left caudate, and left temporal pole, alongside a significant increase in left superior parietal lobe activity, relative to religious singing. A trend toward increased right amygdala activity was observed, and a significant negative correlation was found between singing and glossolalia thalamic laterality indices. Reduced frontal activity was consistent with participants' subjective experience of diminished intentional control. | Glossolalia is characterized by a distinctive neural signature of prefrontal hypoactivation and superior parietal augmentation, reflecting a dissociated state in which the perceived locus of verbal production is experienced as external rather than self-initiated. The pattern diverges from voluntary meditation-induced frontal activation, indicating a qualitatively distinct form of altered religious experience. |
| 97 | (Orme-Johnson et al., 2006) | 2006 | o use functional magnetic resonance imaging (fMRI) to examine how long-term practice of the Transcendental Meditation (TM) technique affects the brain's response to acute thermal pain, using a partial crossover design comparing long-term meditators to healthy controls before and after the controls learned TM. | 24 (12 long-term meditators, 12 healthy controls) | 50% (12 of 24 total; 5 men and 7 women among long-term meditators, 7 men and 5 women among healthy controls) | 56.3 (long-term meditators), 57.8 (healthy controls) | 5.8 (long-term meditators), 3.3 (healthy controls) | fMRI | Not specified |  | Transcendental Meditation (TM) | Thalamus, prefrontal cortex (Brodmann areas 9, 10, 11, 12), anterior cingulate cortex, total brain | At baseline, long-term TM meditators showed 40 to 50 percent fewer voxels responding to pain in the thalamus and total brain compared to healthy controls, with a similar trend in the prefrontal cortex. After the healthy controls learned and practiced TM for five months, their brain response decreased by 40 to 50 percent in the thalamus, prefrontal cortex, and total brain (with a marginal decrease in the anterior cingulate cortex), while the long-term meditators showed no significant further change. At posttest, the two groups no longer differed significantly on any brain measure. Despite these neural differences, both groups reported similar subjective pain intensity ratings at all time points, and both showed a comparable 25 percent decrease in pain ratings from pretest to posttest. | Long-term practice of the Transcendental Meditation technique appears to longitudinally reduce brain reactivity in the affective and motivational dimension of the pain response (thalamus, prefrontal cortex, and total brain), even though it does not change the subjective sensory experience of pain intensity. This dissociation suggests that TM practice reduces distress associated with pain rather than the perceived intensity of the pain itself, likely through mechanisms involving reduced anticipatory anxiety and general stress reactivity. |
| 98 | (Yamamoto et al., 2006) | 2006 | To identify the cortical generators of Transcendental Meditation (TM)-induced frontal and central alpha activity using simultaneous magnetoencephalography (MEG) and EEG, employing multiple source localisation methods. | 16 | 50 | 38.8 | 11.35 | MEG; EEG | hinduism | Meditation | Transcendental Meditation | Medial Prefrontal Cortex (mPFC) , Anterior Cingulate Cortex (ACC) | TM produced significant increases in frontal alpha power and a significant decrease in occipital alpha mean frequency. Using MUSIC, equivalent current dipole, and BESA analyses, alpha dipole sources were consistently localized to the medial prefrontal cortex and anterior cingulate cortex. Six of eight TM participants exhibited two distinct dipole clusters in both regions; the remainder showed a single cluster. Control participants showed no significant changes during the sham task. | TM-induced alpha augmentation originates from the medial prefrontal cortex and anterior cingulate cortex, networks implicated in self-regulation, interoception, and affective monitoring. Continuous generation of high-amplitude diffuse alpha from these regions throughout TM practice is consistent with a state of alert, effortless internal awareness. |
| 99 | (Lutz et al., 2004) | 2004 | To investigate whether long-term Buddhist meditation training induces the self-generation of sustained high-amplitude gamma-band oscillations and large-scale neural phase-synchrony during practice, and to examine whether such training produces lasting baseline neural modifications. | 18 | 0 | 33.44 | 16.63 | EEG | Buddhism | Meditation |  | Medial frontoparietal electrodes, Lateral frontoparietal electrodes, Bilateral parietotemporal electrodes, Midfrontal electrodes, Frontolateral and posterior electrodes, Anterior electrodes | Long-term Buddhist practitioners generated significantly higher-amplitude gamma-band oscillations (25–42 Hz) and greater long-distance phase-synchrony during meditation relative to control participants. Gamma-to-slow-oscillation ratios were substantially elevated in practitioners, including at resting baseline before meditation, and this ratio correlated positively with cumulative hours of practice (r = 0.79, p < 0.02). Gamma amplitude remained elevated post-meditation, indicating carry-over neuroplastic effects. Gamma activity in practitioners was approximately thirty-fold greater than in controls. | Long-term meditation produces both state-dependent and trait-level enhancement of high-amplitude gamma oscillations and long-distance neural synchrony; the highest reported amplitudes in a non-pathological context at the time of publication. These neurophysiological signatures reflect enhanced large-scale temporal integration and suggest durable neuroplasticity from sustained contemplative training. |
| 100 | (Newberg et al., 2003) | 2003 | To quantify changes in regional cerebral blood flow (rCBF) during centering prayer;a verbally-mediated contemplative practice;in Franciscan nuns, and to compare neuroimaging profiles with previously reported data from Tibetan Buddhist meditators employing visualization techniques, thereby demonstrating the feasibility of SPECT neuroimaging across distinct religious traditions. | 11 | 64 |  |  | SPECT | Christianity | Prayer | Prayer | Prefrontal cortex Inferior parietal lobes Inferior frontal lobes Superior parietal lobe Thalamus (right) Dorsolateral prefrontal cortex Orbitofrontal cortex Dorsal medial cortex Cingulate gyrus | Centering prayer was associated with significant increases in rCBF in the prefrontal cortex (7.1%), inferior parietal lobes (6.8%), and inferior frontal lobes (9.0%). A robust inverse correlation was identified between prefrontal cortex and ipsilateral superior parietal lobe perfusion changes (p < .01), and a significant positive correlation was observed between right prefrontal cortex and right thalamic blood flow. No consistent hemodynamic changes were detected in the midbrain, sensorimotor cortices, or temporal lobe. | Increased rCBF in prefrontal, inferior parietal, and inferior frontal regions during meditative prayer, indicative of enhanced neural activity in attentional and language-processing networks. The inverse relationship between prefrontal and superior parietal perfusion suggests prayerful absorption is accompanied by altered spatial self-representation. |
| 101 | (Lo et al., 2003) | 2003 | To investigate the electrophysiological correlates of perception of inner light during Zen Buddhist meditation and to demonstrate the relationship between spiritual experience and measurable EEG alpha dynamics. | 211 | 50 | 43.63 | 7.23 | EEG | Buddhism | Meditation | Zen Meditation | Zen Chakra – located inside the third ventricle Dharma Eye Chakra (Third Eye Chakra) – located at the hypophysis (pituitary gland) Wisdom Chakra – located at the corpora quadrigemina Pineal gland – through which qi energy penetrates The energy passage bridging cerebellum and cerebrum These regions are noted to be located near the visual perceptive pathway. | A significant correlation was identified between perception of inner light and EEG alpha blocking across all six meditating participants. Alpha blocking invariably accompanied participants' signaling of light perception during meditation, and significant alpha suppression was observed during blessing periods. Non-meditating control subjects exhibited no significant EEG changes during the same conditions. EEG patterns transformed from alpha-dominated signals to low-amplitude beta or flattened signals during reported spiritual experiences. | Perception of spiritual inner light during Zen meditation is reliably indexed by alpha suppression and a transition to beta-dominant or flat EEG patterns, consistent with a state of heightened cortical arousal and perceptual clarity. These electrophysiological signatures are specific to experienced meditators, indicating that contemplative expertise is a prerequisite for generating the neurophysiological state underlying this spiritual phenomenon. |
| 102 | (Kjaer et al., 2002) | 2002 | To test whether endogenous dopamine release in the ventral striatum increases during Yoga Nidra meditation, as indexed by reduced 11C-raclopride binding to D2 receptors using positron emission tomography. | 8 | 0 | 40.5 | 6.91 | PET; MRI; EEG | hinduism |  | Yoga Nidra Meditation | Ventral striatum (specifically nucleus accumbens region); also referenced: prefrontal cortex (dorsolateral, anterior cingulate, orbital frontal), cerebellum, thalamus, pallidum, subthalamic nucleus, substantia nigra (in discussion of frontal-subcortical circuits). | A 7.9% decrease in 11C-raclopride binding potential was observed in the ventral striatum during meditation, corresponding to an estimated 65% increase in endogenous dopamine release. This reduction correlated significantly with increased EEG theta activity. Participants concurrently reported decreased readiness for action and heightened sensory imagery, consistent with a state of relaxed, non-directed awareness. | Yoga Nidra meditation produces significant increases in endogenous striatal dopamine release, particularly within the nucleus accumbens region, accompanied by EEG theta augmentation. These findings implicate dopaminergic reward pathways in the subjective phenomenology of contemplative states involving reduced executive engagement. |
| 103 | (Azari et al., 2001) | 2001 | To investigate the neural correlates of religious experience using PET neuroimaging, testing whether religious experience constitutes a cognitive-attributional process mediated by frontal-parietal reasoning networks rather than a pre-conceptual emotional event mediated by limbic structures. | 12 | 33 | 28.5 | 3 | PET | Christianity | Recitation |  | Right dorsolateral prefrontal cortex (BA 9), left dorsolateral prefrontal cortex (BA 9), dorsomedial frontal cortex/pre-SMA (BA 6), right medial parietal cortex/precuneus (BA 7), left cerebellum | During religious recitation of Psalm 23, religiously-practicing participants demonstrated significant activation of a frontal-parietal circuit, including the right and left dorsolateral prefrontal cortex (BA 9), dorsomedial frontal cortex/pre-SMA (BA 6), and right medial parietal cortex/precuneus (BA 7). No limbic area activation was observed. Religious participants successfully induced and maintained a distinct religious state throughout the paradigm. | Religious experience, as elicited by personalized scriptural recitation, is neurally mediated by prefrontal and medial parietal circuits underlying cognitive evaluation, working memory, and voluntary self-regulation rather than by limbic-emotional substrates. These findings support a cognitive-attributional model of religious experience. |
| 104 | (Travis & Wallace, 1999) | 1999 | To compare autonomic and EEG variables during eyes-closed rest versus Transcendental Meditation (TM) practice, and to propose a neural model explaining the physiological mechanisms underlying TM practice. | 20 | 35 | 27.8 |  | EEG | Not Specified |  | Meditation | Frontal cortex, central (sensorimotor) region, parietal cortex, thalamus (specific and non-specific nuclei), nucleus reticularis thalami, basal ganglia, midbrain reticular activating system | Meditation sessions showed significantly lower breath rates, lower skin conductance levels, higher respiratory sinus arrhythmia, and higher frontal and anterior-posterior EEG alpha coherence compared to eyes-closed rest. Alpha power itself did not differ significantly between conditions. These changes appeared within the first minute of TM practice and were sustained throughout the 10-minute session. Heart rate, EEG laterality, and alpha percentage change scores showed no significant differences between conditions. | Meditation practice produces a physiological state distinct from ordinary eyes-closed rest, marked by a rapid (within one minute) cascade of autonomic and central nervous system changes that are maintained through a "restfully alert" state. |
| 105 | (Don et al., 1998) | 1998 | To examine the EEG effects of ayahuasca ingestion on brain electrical activity in humans, with particular focus on changes in the 36–44 Hz ("40 Hz") frequency band and its relationship to conscious experience and visual imagery. | 11 | 45 | 35.8 |  | EEG | Santo Daime | Ayahuasca Ritual | Ayahuasca Ritual Ingestion | Left occipital cortex (O1) – primary visual cortex  Left posterior temporal cortex (T5)  Parietal cortex (P3, Pz, P4)  Central cortex (C3, Cz)  Right posterior temporal lobe (T6)  Right occipital lobe (O2) The study also discusses the thalamocortical system, specifically: Thalamic reticular nucleus Nonspecific thalamic nuclei (intralaminar complex) Specific thalamic (sensory) nuclei | Ayahuasca produced significant increases in 40 Hz (36–44 Hz) power in left occipital-temporal-parietal scalp electrodes under eyes-closed conditions, extending to most of the posterior scalp under eyes-open conditions. Tendencies toward decreased slow-rhythm (theta and alpha) power and increased beta activity (14–30 Hz) were consistent with prior hallucinogen research. Enhanced 40 Hz activity was interpreted as reflecting activation of the visual cortex and thalamocortical synchronization amplifying endogenous imagery. | Ayahuasca produces systematic augmentation of gamma-band (40 Hz) oscillations in posterior cortical regions, reflecting enhanced thalamocortical synchronization and amplification of internally-generated visual processing. The concurrent desynchronization of slow rhythms indicates a state of heightened arousal in which the boundaries between internally-generated imagery and external perception are blurred. |

Notes: Abbreviations used in the Instruments column= DTI: Diffusion Tensor Imaging; ECG: Electrocardiography; EEG: Electroencephalography; ERP: Event-Related Potential; fMRI: Functional Magnetic Resonance Imaging; GSR: Galvanic Skin Response; HRV: Heart Rate Variability; MEG: Magnetoencephalography; LC/MS/MS: Liquid Chromatography with tandem Mass Spectrometer; MRI: Magnetic Resonance Imaging (Structural); N/A: No Neurological Instrument Reported; PET: Positron Emission Tomography; qPCR: Quantitative Polymerase Chain Reaction (also called real-time PCR); SPECT: Single-Photon Emission Computed Tomography; sLORETA: standardized low-resolution electromagnetic tomography
