# Supplementary material for: The Neuroscience of Religious and Spiritual Practices: A Systematic Review of Neurotheological Evidence
Source: Brain Behav. 2026 Aug 30;16(9):e71733. doi: 10.1002/brb3.71733 (PMC13527413; doi:10.1002/brb3.71733)
Supplement: Supplementary file 3 [file BRB3-16-e71733-s003.docx]

*Supplementary Material 1: PRISMA Checklist*

| **Item** | **PRISMA Requirement** | **Reported** | **Location** |
| --- | --- | --- | --- |
| 1 | Title: Identify report as systematic review | Yes | Title |
| 2 | Abstract: Structured summary with background, objectives, methods, results, conclusions | Yes | Abstract |
| 3 | Rationale: Describe rationale for review | Yes | Section 1.4 |
| 4 | Objectives: Explicit statement of objectives | Yes | Section 1.4 and Abstract |
| 5 | Eligibility criteria: Specify inclusion and exclusion criteria | Yes | Section 2.3 |
| 6 | Information sources: Databases, registers, and other sources searched | Yes | Section 2.4 |
| 7 | Search strategy: Full search strings for all databases | Yes | Section 2.4 |
| 8 | Selection process: Method of screening and selecting studies | Yes | Section 2.6 |
| 9 | Data collection process: Method of extracting data | Yes | Section 2.7 |
| 10 | Data items: List of all variables extracted | Yes | Section 2.7 |
| 11 | Study risk of bias: Methods for assessing risk of bias | Yes | Section 2.10 |
| 12 | Effect measures: Specify effect measures used | Not applicable |  |
| 13 | Synthesis methods: Describe synthesis approach | Yes | Section 2.9 |
| 14 | Reporting bias assessment: Methods for assessing reporting bias | Partial | Section 4.4 |
| 15 | Certainty assessment: Methods for assessing certainty of evidence | Yes | Section 2.4 |
| 16 | Study selection results: Numbers at each stage with reasons for exclusion | Yes | Section 2.6 |
| 17 | Study characteristics: Present characteristics of included studies | Yes | Section 3.1 |
| 18 | Risk of bias in studies: Present risk of bias assessments | Yes | Section 2.10 |
| 19 | Results of individual studies: Report results for each study | Yes | Sections 3.2 to 3.6 |
| 20 | Results of syntheses: Present synthesis results | Yes | Section 3.7 |
| 21 | Reporting biases: Report results of investigations into reporting bias | Partial | Section 4.4 |
| 22 | Certainty of evidence: Present assessments of certainty | Yes | Sections 2.10 and 4.4 |
| 23 | Discussion: Provide general interpretation of results | Yes | Section 4.1 |
| 24 | Limitations: Discuss limitations of review | Yes | Section 4.4 |
| 25 | Conclusions: Provide general conclusions | Yes | Section 5 |
| 26 | Registration: Provide registration number and name of register | Partial | Section 2.1 |
| 27 | Competing interests and funding: Declare competing interests and funding sources | Yes | Author disclosure page |
